# Programming permanent and transient molecular protection *via* mechanical stoppering†
†Electronic supplementary information (ESI) available. See DOI: 10.1039/c9sc03744f


**DOI:** 10.1039/c9sc03744f

**Published:** 2019-10-04

**Authors:** Miguel A. Soto, Francesco Lelj, Mark J. MacLachlan

**Affiliations:** a Department of Chemistry , University of British Columbia , 2036 Main Mall , Vancouver , BC , V6T 1Z1 Canada . Email: mmaclach@chem.ubc.ca; b La.M.I. and LaSCAMM INSTM Sezione Basilicata , Dipartimento di Chimica , Università della Basilicata , via dell'Ateneo Lucano 10 , Potenza , 85100 Italy; c Quantum Matter Institute , University of British Columbia , 2355 East Mall , Vancouver , BC , V6T 1Z4 Canada; d WPI Nano Life Science Institute , Kanazawa University , Kanazawa , 920-1192 Japan

## Abstract

Chemical protection is an essential tool in synthetic chemistry, which involves blocking reactive sites on a molecule through covalent bonds. Physical approaches, such as encapsulation and host-mediated protection, have emerged as interesting alternatives that use steric bulk to inhibit reactivity. Here, we report the protection of a redox-active viologen through its incorporation into mechanically interlocked molecules (MIMs), namely hetero[4]rotaxanes. The viologen was confined inside a host cavity and flanked by two mechanical stoppers, which allowed for permanent and transient protection. Deprotection occurred on-demand *via* an unstoppering process, triggered by a proton transfer, polarity effect, or a thermal stimulus. We anticipate that permanent and transient mechanical stoppering could be incorporated into devices to function as molecular probes, transport/delivery systems, or stimuli-controlled degradable materials.

## Abstract 2

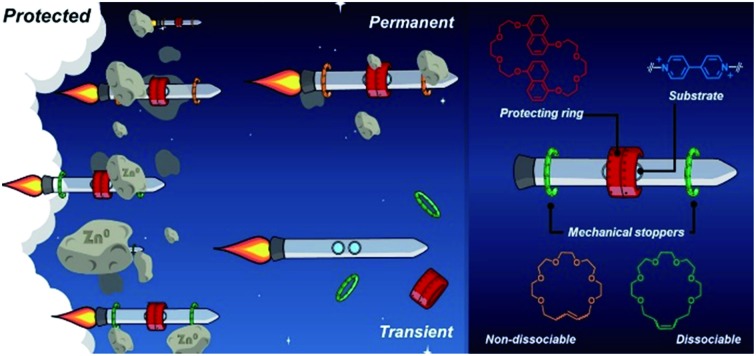
A macrocycle (permanently or transiently) protects a viologen from heterogenous reduction, all thanks to bespoke mechanical stoppering.

## Introduction

Since the introduction of the mechanical bond, numerous mechanically interlocked molecules (MIMs) have been synthesized. [2]Rotaxanes and [2]catenanes are the “simplest” and most documented MIMs,^1^–^5^ though many other exotic species have been accessed, *e.g.* hetero[*n*]rotaxanes,^6^–^9^ rotacatenanes,^10^,^11^ knot-capped rotaxanes,^12^ and main-chain poly[*n*]catenanes.^13^ Currently, research on MIMs involves the synthesis of even more intricate assemblies,^14^ while also focusing on the operation of correlated dynamic processes,^15^ and the quest for function, *i.e.*, MIMs performing as catalysts,^16^ sensors,^17^,^18^ drug carriers,^19^ molecular electronics,^20^ and machines.^21^

One intriguing approach to achieve function with MIMs is the concept of mechanical protection,^22^*i.e.* the shielding of an environment-susceptible substrate by its confinement in the interior of a macrocycle. In this methodology the physical and chemical properties of a substrate (*e.g.* melting point, solubility, reactivity or degradation) are dramatically altered, so that it no longer behaves as unprotected.^23^ Despite the attractiveness of this approach for synthetic chemistry, only a few MIMs have been prepared with the intention of using mechanical protection.^24^–^36^ Typically in these examples, the substrate (or a modified form of it) acts as a recognition site for the protecting macrocycle, then the implementation of an appropriate protocol (*e.g.* stoppering or clipping) generates a MIM where the macrocycle is secured and provides permanent protection to the substrate. In some specific systems, the protection can be reversed through the degradation of one of the MIM components, which requires a specific input such as an organometallic catalyst^22^ or an enzyme.^37^

**Fig. 1:**
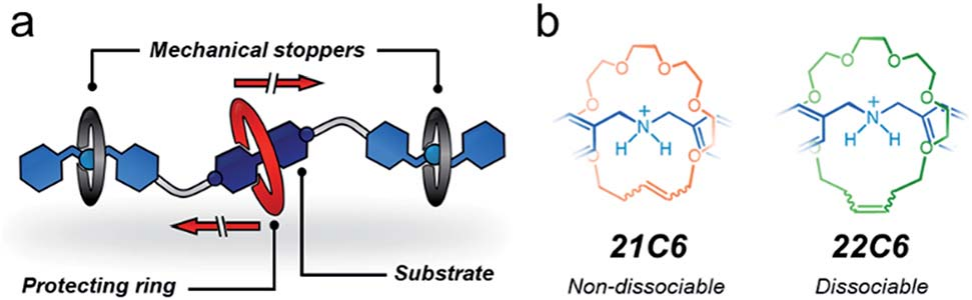
Schematic representation of (a) a hetero[4]rotaxane structure, and (b) permanently interlocked (left) and metastable (right) rotaxanes constructed from a dibenzylammonium unit and crown ether rings.

Overall, both protection approaches, permanent and transient, are equally relevant. A permanent effect is appropriate to preserve or enhance the properties and function of a substrate during a process,^38^,^39^ whereas transient protection permits the controlled release of the active substrate once the adverse environment has been evaded.^37^ Here, we present a strategy to protect a molecule both temporarily and permanently by using bespoke stoppering on MIMs. To pursue our concept, we designed two hetero[4]rotaxanes that are depicted in Fig. 1a. These MIMs contain three macrocycles threaded onto a linear species; the central macrocycle functions as a protective unit while the outer macrocycles prevent it from escaping. The outer rings and the thread end-groups act in tandem as mechanical stoppers^40^ to preserve a protected environment around the substrate.

To program both permanent and transient protection, we relied on positioning metastable and permanently interlocked species as mechanical stoppers. Dasgupta and Wu recently demonstrated that the dibenzylammonium cation can be encircled by [21] and [22]-membered crown ethers (**21C6**, **22C6**) to yield [2]rotaxanes using the clipping method.^41^ The one-atom difference in the macrocyclic structures (21 *vs.* 22) strongly affects the stability of the MIMs (Fig. 1b). A [2]rotaxane containing **21C6** is permanently interlocked, so it does not disassemble without the cleavage of a covalent bond. Conversely, the **22C6**-containing species is isolable but prone to disassemble upon chemical or physical stimulation. Based on these previous observations, we selected the macrocycles **21C6** and **22C6**, and the dibenzylammonium motif for the fabrication of two different mechanical stoppers: a dissociable set and a non-dissociable counterpart.

We chose the 4,4′-bipyridinium unit, a viologen, as the substrate to protect as these redox-active cations are readily accessible, undergo chemical reduction with a macroscopic readout (color change), and can assemble with several classes of macrocycles, such as pillar[*n*]arenes, cucurbit[*n*]urils and crown ethers.^42^ Considering its tight fit with the viologen derivatives and its relative size and aspect ratio, with respect to **21C6** and **22C6** (see Fig. S1†),^43^ we selected the crown ether 1,5-dinaphtho[32]crown-8 (**DN32C8**) as the protective unit.

## Results and discussion

**Scheme 1:**
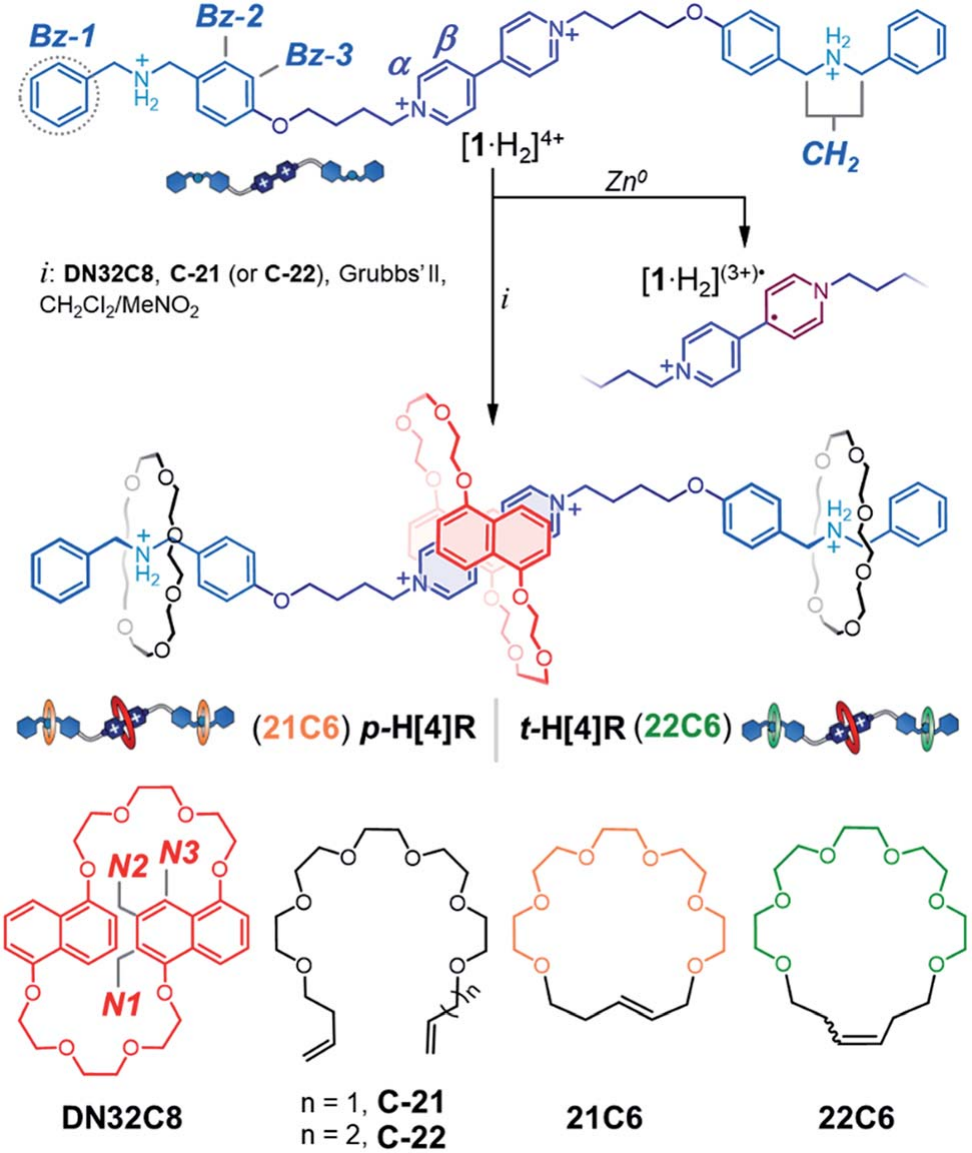
Syntheses of the two designed hetero[4]rotaxane structures and a thread-like radical cation. Chemical structures and proton assignments for the macrocyclic structures and thread unit are also shown. Note that the radical cation is delocalized.

With the targeted components identified, we synthesized [**1**·H_2_]^4+^ (Scheme 1), a linear tetracation composed of a 4,4′-bipyridinium core that is spaced from two dibenzylammonium moieties by oxybutylene chains. [**1**·H_2_][PF_6_]_4_ was prepared in four steps, in 80% overall yield, and characterized by HRMS, NMR, and UV-vis spectroscopy (see ESI†). As with other viologen derivatives, [**1**·H_2_]^4+^ undergoes a one-electron reduction when zinc dust is added to a solution of [**1**·H_2_]^4+^.^44^,^45^ We found that this process is fast (<5 min) and leads to a visible color change. Reduction of [**1**·H_2_]^4+^ in acetonitrile (pale yellow solution) generates the blue radical cation [**1**·H_2_]^(3+)^˙ that features structured bands in the UV-vis spectrum, centered at 375 and 575 nm (Fig. S2†).

To protect [**1**·H_2_]^4+^ from reduction, we first investigated the self-assembly of [**1**·H_2_]^4+^ with the protecting ring **DN32C8**, and tethers **C-21** and **C-22**. The addition of one equiv of **DN32C8** to an acetonitrile solution of [**1**·H_2_][PF_6_]_4_ causes a rapid color change from pale yellow to red. This color change is ascribed to the formation of a charge-transfer (CT) complex between the electron-rich **DN32C8** and the electron-poor core of [**1**·H_2_]^4+^; this complex produces an absorption band at 475 nm in the UV-vis spectrum. Furthermore the ^1^H NMR spectrum showed an upfield shift for the α and β resonances of [**1**·H_2_]^4+^ upon mixing with **DN32C8** (Δ*δ*_α_ = 0.1 ppm and Δ*δ*_β_ = 0.3 ppm), but the dibenzylammonium signals were unaffected (Fig. S3†). These observations suggest that the macrocycle **DN32C8** spontaneously threads onto [**1**·H_2_]^4+^ and sits on the bipyridinium core to yield the pseudorotaxane [**1**·H_2_⊂**DN32C8**]^4+^ [Δ*G*_asso_ = –(12.8 ± 0.2) kJ mol^–1^, CD_3_CN, 25 °C], see Table S1.† These spectroscopic data and complex stability resemble those of other reported [2]pseudorotaxane complexes composed of bipyridinium–**DN32C8** pairs.^46^

**Fig. 2:**
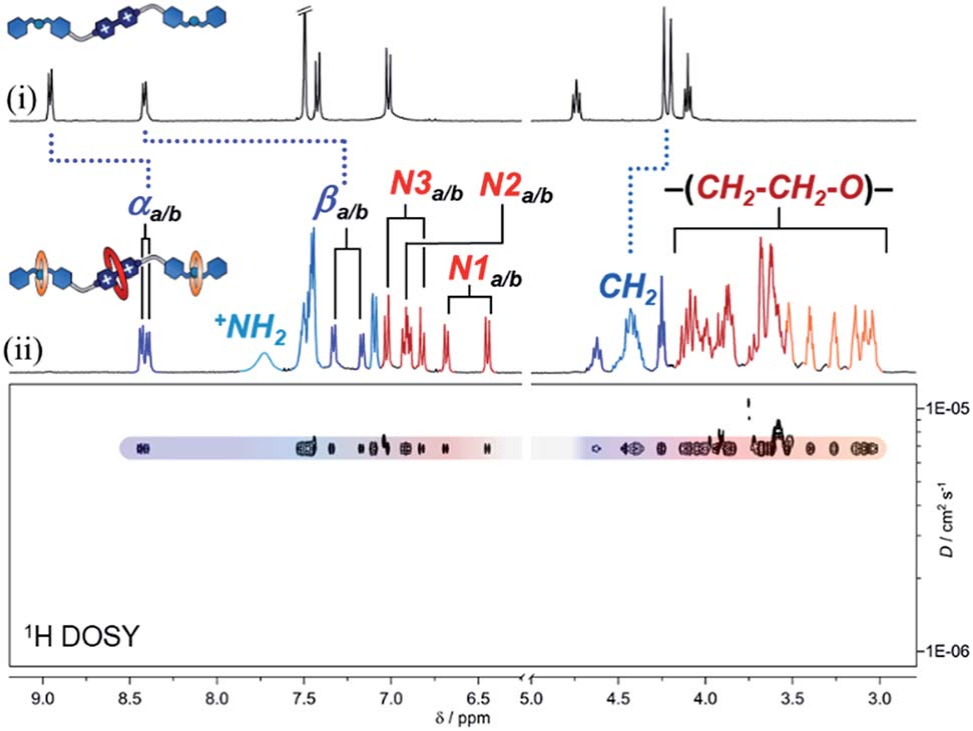
^1^H NMR spectra (400 MHz, CD_3_CN) of (i) compound [**1**·H_2_][PF_6_]_4_ and (ii) hetero[4]rotaxane **p-H[4]R**. ^1^H DOSY NMR spectrum is shown in the bottom portion.

Addition of the precursor **C-21** or **C-22** to a solution containing [**1**·H_2_⊂**DN32C8**]^4+^ did not interfere with pseudorotaxane formation. Indeed, all components self-sort: **DN32C8** encircles the bipyridinium motif, while the glycolic tethers (**C-21** or **C-22**) solely wrap the ammonium stations. This was confirmed by the observation of a downfield shift on the ^+^NH_2_ resonance (Δ*δ*_^+^NH_2__ = 0.2 ppm) in the ^1^H NMR spectrum (Fig. S5†). Addition of second-generation Grubbs' catalyst to any of the mixtures containing **DN32C8**, [**1**·H_2_]^4+^, and **C-21** (or **C-22**), followed by heating, led to ring-closure of the glycolic tethers, securing the **DN32C8** ring. This reaction yielded the transiently (t) and permanently (p) protected hetero[4]rotaxanes **t-H[4]R** and **p-H[4]R** in 30% and 37% yield, respectively (see ESI†).^47^Fig. 2 shows a representative ^1^H NMR spectrum for one of the isolated species, rotaxane **p-H[4]R** (**t-H[4]R** data are in Fig. S6†).

Using 2D NMR spectroscopy (Fig. S7–S9†), we identified four principal regions in the ^1^H NMR spectrum: (i) bipyridinium resonances at 8.43/8.40 ppm (α_a/b_) and 7.3/7.2 ppm (β_a/b_); (ii) naphthalene groups at 7.0/6.8 ppm (N1_a/b_), 6.9 ppm (N2_a/b_), and 6.7/6.5 ppm (N3_a/b_); (iii) glycol chains (for the three crown ether rings) at 3.0–4.1 ppm; and (iv) dibenzylammonium signals at 7.7 (^+^NH_2_), 7.5–7.1 (Bz1–3) and 4.4 ppm (CH_2_). Integration of the peaks in the ^1^H NMR data confirmed the stoichiometry of the assembled MIM to be one thread, two **21C6** rings, and one **DN32C8** crown ether. This was supported by HRMS, where the parent ion [**1** + **DN32C8** + (2 × **21C6**) + 2H]^4+^ was detected at *m*/*z* = 462.7604. Furthermore, we proved by diffusion-ordered NMR spectroscopy (DOSY, Fig. 2) that all observed resonances in the ^1^H data correspond to a single species that diffuses in solution at *ca.* 6.8 × 10^–6^ cm^2^ s^–1^ (CD_3_CN, 25 °C), *i.e.* rotaxane **p-H[4]R**.

The ^1^H NMR spectrum of **p-H[4]R** shows two major differences from that of pure [**1**·H_2_][PF_6_]_4_. First, the bipyridinium resonances α and β show an upfield shift 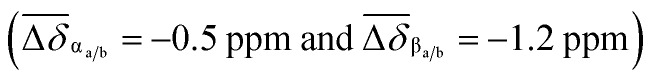, which is attributed to the confinement of the substrate inside the **DN32C8** cavity that leads to shielding from the naphthalene rings. Second, the dibenzylammonium protons (^+^NH_2_, Bz, and CH_2_) moved downfield (Δ*δ*_^+^NH_2__ = 0.3 ppm) because of hydrogen bonding with the **21C6** rings. The relative position of the [21] and [32]-membered macrocycles, with respect to the thread stations, was supported by NOESY NMR spectroscopy, where through-space couplings were identified for each individual host–guest pair, *i.e.* dibenzylammonium–**21C6** and bipyridinium–**DN32C8** (Fig. S8†).

Interestingly, the set of aromatic signals for the bipyridinium–**DN32C8** pair splits into two groups (a and b) in the ^1^H NMR spectrum. According to a 2D EXSY experiment (Fig. S9†), both a and b sets are exchanging at room temperature, indicating the appearance of two isomers that presumably interconvert by an *anti*/*syn* isomerization of **DN32C8** (Scheme S4†). A [3]rotaxane that does not contain this macrocycle features a single set of resonances in the NMR spectrum (see Fig. S10†), thus confirming that both isomers of **p-H[4]R** emerge as a consequence of the dynamics on **DN32C8**.^48^

**Fig. 3:**
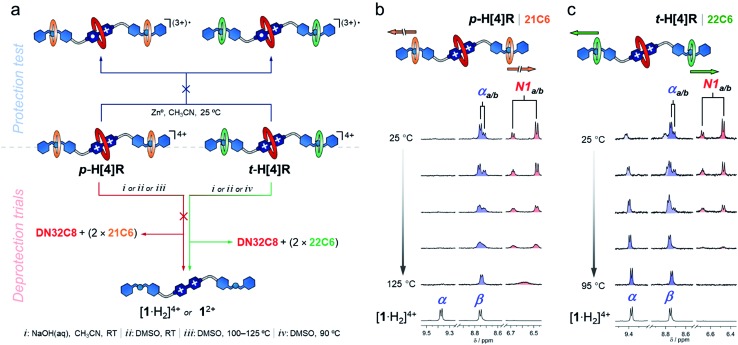
(a) A scheme showing the tests performed on **p-H[4]R** and **t-H[4]R**: deprotection attempt (top) and controlled unstoppering (bottom). (b and c) Partial ^1^H VT-NMR spectra (400 MHz, DMSO-*d*_6_) of (b) **p-H[4]R** and (c) **t-H[4]R**.

After characterizing the hetero[4]rotaxane molecules, we interrogated them against chemical reduction (Fig. 3a). Separate solutions of **p-H[4]R** and **t-H[4]R** in CH_3_CN were loaded with zinc dust and stirred under a N_2_ atmosphere for one day. We did not observe any visible change. UV-vis spectroscopy showed that both solutions preserved their characteristic CT absorption band, at 475 nm, and the radical cation [**1**·H_2_]^(3+)^˙ was not detected (Fig. S11†), confirming that **DN32C8** effectively protects the bipyridinium substrate in **p-H[4]R** and **t-H[4]R**. The lack of reactivity of the viologen within the rotaxanes is in stark contrast to [**1**·H_2_]^4+^, which reacts rapidly (<5 min) with zinc metal under the same conditions.

Next, we attempted to deprotect both hetero[4]rotaxanes using controlled stimuli. The deprotection step should occur *via* disassembly of (at least) one of the mechanical stoppers by the disruption of the intercomponent hydrogen bonding. To disable these interactions, we tried three approaches: (i) deprotonation of the ammonium groups; (ii) use of a high polarity solvent; and (iii) application of a thermal stimulus (Fig. 3a). These triggers were first tested on rotaxane **p-H[4]R** and then on **t-H[4]R**.

An CD_3_CN solution of **p-H[4]R** was treated with one equiv of base (1 M NaOH_(aq)_) at room temperature. According to the recorded NMR experiment (Fig. S12†), the addition of base did not cause proton transfer; the ^+^NH_2_ resonance was still observed at 7.7 ppm and other **p-H[4]R** signals were unaffected. Furthermore, the molecular ion [**1** + **DN32C8** + (2 × **21C6**) + 2H]^4+^ was identified by HRMS (*m*/*z* = 463.0509) while none of the individual components (**1**, **DN32C8** or **21C6**) were detected. Thus, deprotonation of **p-H[4]R** is inaccessible, probably because of the tight fit between the dibenzylammonium unit and the **21C6** macrocycle. Switching from CH_3_CN to DMSO (a highly competitive solvent) did not produce a different result, and only a change of the *anti*/*syn*-**DN32C8** isomers ratio was observed, from 1 : 1 in CD_3_CN to 2 : 1 in DMSO-*d*_6_. Disassembly of **p-H[4]R** was not achieved even after several weeks at room temperature in DMSO (see Fig. S14†).

To create a more severe environment, we heated a solution of **p-H[4]R** in DMSO-*d*_6_ from 25 to 125 °C and analyzed it by ^1^H NMR spectroscopy. Fig. 3b shows the partial aromatic region of the collected spectra (full data in Fig. S15†). Below ∼100 °C, both **p-H[4]R** isomers were clearly identified; holding *T* at 100 °C for one hour did not damage **p-H[4]R**. Elevating the temperature above 115 °C only caused coalescence of the resonances assigned to the isomers, indicating that their interconversion becomes faster at higher temperatures. We calculated an exchange rate of 110 s^–1^ at the coalescence temperature (see ESI†). Furthermore, a **p-H[4]R** rotaxane solution prepared in DMSO, and loaded with zinc dust, did not degrade after storage for six months under a N_2_ atmosphere (Fig. S19†). Together, our observations suggest that **p-H[4]R** is permanently stoppered and therefore the bipyridinium substrate is permanently protected. This protection effect is ascribed to (1) the steric shield provided by **DN32C8**, and (2) the electronic stabilization given by this macrocycle to the viologen substrate; cyclic voltammetry experiments (Fig. S20†) revealed a stabilization of ∼13 kJ mol^–1^ with respect to the unprotected species [**1**·H_2_]^4+^. Noteworthy, there is only one way to deprotect **p-H[4]R**: by breaking covalent bonds. Rotaxane **p-H[4]R** was fully disassembled by controlled ring-opening of the **21C6** macrocycles, which ultimately released the protecting unit (Fig. S21†).

In contrast, we expected that incorporating **22C6** rings into the hetero[4]rotaxane structure would allow transient protection, and breakage of covalent bonds would not be required. We therefore applied the same tested stimuli to **t-H[4]R**. Addition of one equivalent of base (NaOH_(aq)_) to a CH_3_CN solution of **t-H[4]R** produced an immediate change in the color of the solution from red to pale yellow, and the characteristic CT band in the UV-vis spectrum disappeared. This indicated that the proton transfer process occurred rapidly and caused two subsequent events: unstoppering followed by the release of the protecting ring. Although the process was too fast (<1 min) to detect the deprotonated form of **t-H[4]R** by ^1^H NMR spectroscopy, we observed the free thread **1**^2+^ and macrocycles **21C6** and **DN32C8** in solution by both NMR spectroscopy and HRMS (Fig. S22–S23†).

Dissolving **t-H[4]R** in a polar solvent also led to rapid unstoppering. After dissolving **t-H[4]R** in DMSO-*d*_6_ at 25 °C, we observed three new sets of resonances in the ^1^H NMR spectrum (Fig. 3c) that correspond to the free components **22C6**, **DN32C8** and [**1**·H_2_]^4+^. Integration of the ^1^H NMR data suggests that 20% of the complex was rapidly disassembled. Heating the sample up to 95 °C led to full unstoppering, so that the rotaxane **t-H[4]R** resonances were no longer evident, while the bipyridinium signals at 9.4 (α) and 8.7 (β) ppm reached maximum intensity. As well, the free species **DN32C8**, [**1**·H_2_]^4+^ and **22C6**, were all found in solution by HRMS (see Fig. S25†).

After we proved that **t-H[4]R** undergoes controlled unstoppering, we attempted to use this deprotection as a means to control the reactivity of the viologen. A solution of **t-H[4]R** in CH_3_CN was loaded with zinc dust, sparged with N_2_, and left to settle for one hour. Within this period, we did not observe any change, neither macroscopic nor spectroscopic. This implies that the **DN32C8** unit stays in place protecting the viologen, an effect that cannot be achieved in systems that are under dynamic assembly/disassembly.^45^ Conversely, upon addition of an alkaline solution (1 M NaOH_(aq)_, 1.0 equiv.) the mixture rapidly changed color from red to blue. We ascribe this to the unstoppering/deprotection/reduction sequence that gives rise to [**1**·H_2_]^(3+)^˙ in solution (Fig. S26†) along with residual **DN32C8** and **22C6** units.

**Fig. 4:**
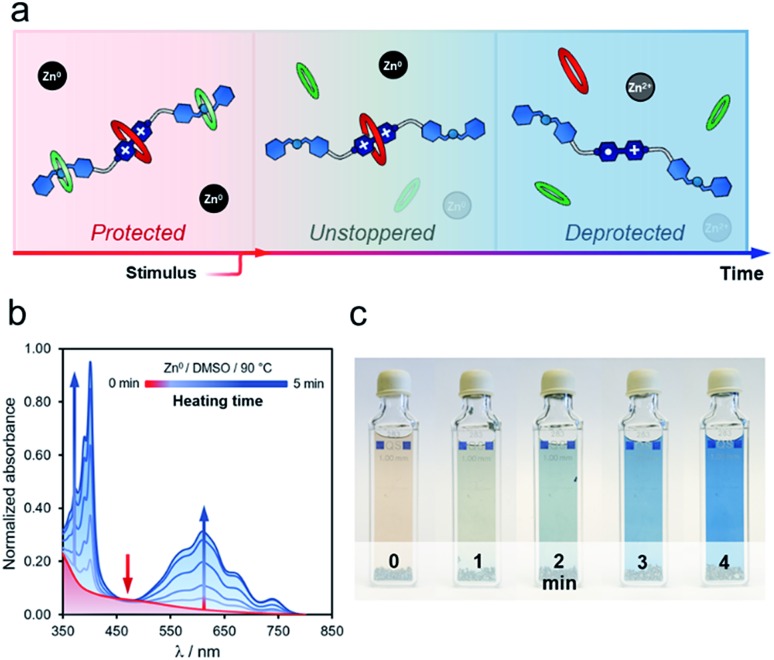
(a) Gradual deprotection/reduction sequence. Experiment conducted on rotaxane **t-H[4]R** (DMSO, 90 °C) and followed at macroscopic scale and by UV-vis spectroscopy (b and c).

Heating **t-H[4]R** in DMSO, in the presence of zinc metal, rendered similar results, although the unstoppering followed by deprotection appeared to be gradual (Fig. 4a). After heating for one minute at 90 °C, we observed a drop in the intensity of the characteristic CT band at 475 nm, and two additional bands assigned to [**1**·H_2_]^(3+)^˙ (375 and 575 nm) were present (Fig. 4b). Continuous heating at the same temperature produced a concomitant increase of these bands. Interestingly, the deprotection/reduction process was detected at the macroscopic scale. The pale red solution that contained **t-H[4]R** gradually transformed into a blue mixture in *ca.* five minutes at 90 °C (Fig. 4c), due to the presence of [**1**·H_2_]^(3+)^˙, *i.e.* once the unstoppering process was completed.

## Conclusions

In summary, we have synthesized two hetero[4]rotaxane MIMs that resist chemical reduction. Both molecules bear two key components that, in synergy, make protection viable: (1) a central ring that offers steric bulk and stabilizes the sensitive substrate, and (2) a couple of outer macrocycles that prevent the protecting unit from escaping, *i.e.* the mechanical stoppers.

By selecting between two stoppers, comprising [21] or [22]-membered rings, we programmed permanent and transient protection on **p-H[4]R** and **t-H[4]R**, respectively. **p-H[4]R** does not disassemble even when exposed to severe environments, whereas **t-H[4]R** dissociates to leave the sensitive substrate exposed, on demand. We consider that mechanical stoppering, as a general concept, could be valuable for the design of, *e.g.*, other protected MIMs bearing relevant substrates, delivery systems, and stimuli-controlled degradable materials.

## Conflicts of interest

There are no conflicts to declare.

## Supplementary Material

Supplementary information
