# Supplementary material for: Programming permanent and transient molecular protection via mechanical stoppering
Source: Chem Sci. 2019 Oct 4;10(44):10422–7. doi: 10.1039/c9sc03744f (PMC6988755; doi:10.1039/c9sc03744f)
Supplement: Supplementary file 1 [file SC-010-C9SC03744F-s001.pdf]

Electronic Supplementary Material (ESI) for Chemical Science.
This journal is © The Royal Society of Chemistry 2019

Supporting information for

Programming Permanent and Transient Molecular Protection via Mechanical Stoppering

Miguel A. Soto,[a] Francesco Lelj[b] and Mark J. MacLachlan*[a,c,d]

[a] *Department of Chemistry, University of British Columbia, 2036 Main Mall, Vancouver, BC, V6T 1Z1 Canada*

[b] *La.M.I. and LaSCAMM INSTM Sezione Basilicata, Dipartimento di Chimica, Università della Basilicata, via dell'Ateneo Lucano 10, Potenza, 85100 Italy*

[c] *Quantum Matter Institute, University of British Columbia, 2355 East Mall, Vancouver, BC, V6T 1Z4 Canada*

[d] *WPI Nano Life Science Institute, Kanazawa University, Kanazawa, 920-1192 Japan*

Table of contents

General S3

Ring-size comparison S4

Viologen reduction S5

Self-assembly of pseudorotaxane [**1**·H_2⊂(**21C6**)$_2$]$^{4+}$ S6

Self-sorting process S8

Selected NMR characterization of ***p*-H[4]R** S10

^{1}H NMR spectrum of a model [3]rotaxane S12

Reduction attempts on the hetero[4]rotaxane structures S13

Unstoppering attempts on ***p*-H[4]R** S14

  Base trigger S14

  Polarity and temperature effect S16

Isomer interconversion process S18

Deprotection attempt on ***p*-H[4]R** S28

Cyclic voltammetry analyses S29

Rotaxane (***p*-H[4]R**) degradation *via* ring-opening metathesis S31

Unstoppering process on ***t*-H[4]R** S32

  Base trigger S32

  Polarity and temperature stimuli S34

Controlled deprotection of ***t*-H[4]R** S36

  Base trigger S36

  Polarity and temperature effect S37

Synthesis S38

  Functionalized benzaldehyde **A** S39

  4,4'-Bipyridinium salt **B**[PF_6]$_2$ S41

  Thread [**1**·H_2][PF_6]$_4$ S43

  [3]Rotaxane [**1**·H_2⊂(**21C6**)$_2$][PF_6]$_4$ S45

  General synthesis of the hetero[4]rotaxane structures S50

References S64

General

All commercially available chemicals were purchased from Sigma-Aldrich and Oakwood Chemicals and used without further purification. Dry dichloromethane (CH_2Cl_2) and tetrahydrofuran (THF) were collected from an Inert PureSolv MD5 purification system, whereas nitromethane ($MeNO_2$) and acetonitrile (MeCN) were dried with freshly activated 5 Å and 3 Å molecular sieves, respectively. Deuterated solvents (CD_3CN, $CDCl_3$, and DMSO-d_6) were purchased from Cambridge Isotope Laboratories and Sigma-Aldrich. Flash column chromatography was carried out using SiliCycle (230–400 mesh) silica gel as the stationary phase. Nuclear magnetic resonance (NMR) experiments were recorded on Bruker AVIII HD 400 MHz and Bruker Avance 400 MHz spectrometers; ^{1}H and ^{13}C NMR chemical shifts (δ) are given in parts per million (ppm) relative to TMS, using the residual solvent signal for calibration. *J* values are reported in Hz, and signal multiplicity is denoted as s (singlet), d (doublet), t (triplet), m (multiplet) and br (broad signal). UV-vis spectra were recorded on a Cary 5000 UV-vis-NIR spectrometer, employing 1 mm pathlength quartz cuvettes. High-resolution mass spectra (HRMS) were recorded on an ESI-TOF Waters Micromass LCT spectrometer. Cyclic voltammograms were acquired using an AFCBP1 potentiostat from Pine Instrument company. Macrocycle **DN32C8**,[1] and precursors **C-21**[2] and **C-22**,[3] were synthesized following reported methodologies and all spectroscopic characterization matched with the published data.

Ring-size comparison

Figure S1 shows the macrocycles *trans*-**21C6**, *trans*-**22C6** and **DN32C8** as found in reported [2]rotaxane structures.[1,2]

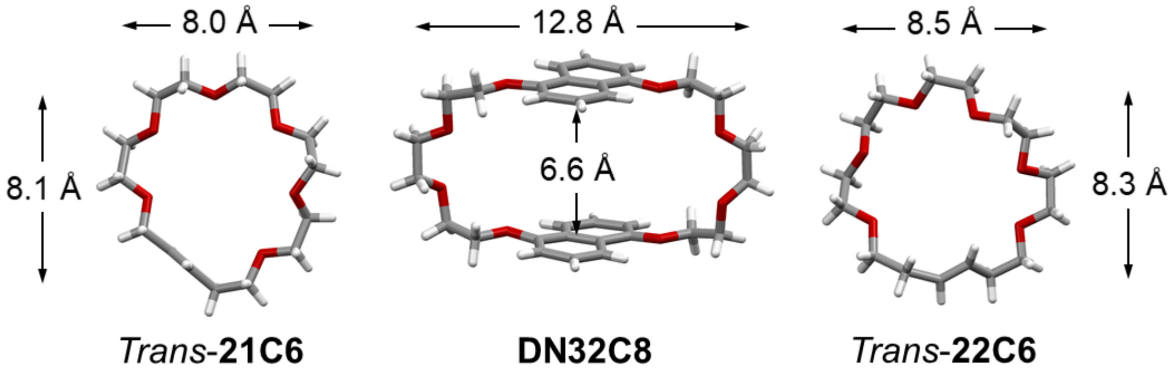

Figure S1. Solid-state structures of the chosen macrocyclic scaffolds. The thread species hosted in their cavities have been omitted for clarity.

Viologen reduction

A deaerated solution of viologen [**1**·H_2][PF_6]$_4$ in MeCN (2×10^{-3} M, 400 μL) was loaded with zinc dust (10 mg) and stirred for 10 min under a N_2 atmosphere, causing the solution to change color from yellow to blue. Figure S2 shows the UV-vis spectra of the viologen before and after its reduction. Radical cation was identified by two structured bands at λ_{max} (ε) of 400 (2560) and 610 nm (720 $mol^{-1}\,dm^{3}\,cm^{-1}$).

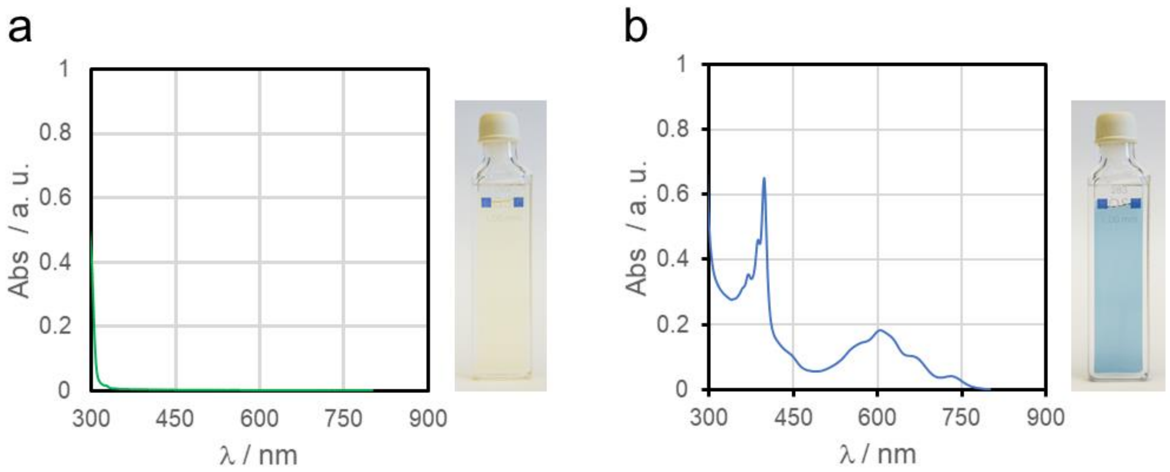

Figure S2.UV-vis spectra (MeCN, 25 ºC) of [**1**·H_2][PF_6]$_4$ a) before and b) after chemical reduction with Zn^0. The photographs show the analyzed samples.

Self-assembly of pseudorotaxane [1·H$_2$⊂(21C6)$_2$]$^{4+}$

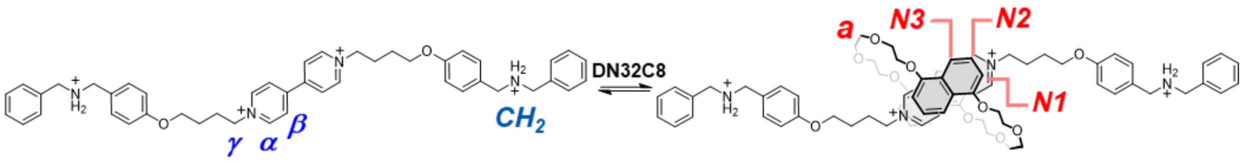

Scheme S1. [2]pseudorotaxane formation and proton assignment.

The thread molecule [**1**·H$_2$][PF$_6$]$_4$ and macrocycle **DN32C8** were mixed in a 1:1 ratio in CD$_3$CN (or MeCN) and analyzed by ^{1}H NMR, UV-vis spectroscopy, and high-resolution mass spectrometry. Changes in the NMR data (Figure S3a), for both the host and guest resonances, suggest the formation of the [2]pseudorotaxane [**1**·H$_2$⊂(**21C6**)$_2$]$^{4+}$, which is stabilized by π-π stacking interactions. UV-vis spectroscopy reveals an absorption band centered at 475 nm (Figure S3b), which is ascribed to the formation of a charge transfer complex within the pseudorotaxane. Additionally, the parent ion [**1** + **DN32C8** + 2H + 2PF$_6$]$^{2+}$ was detected by HRMS at *m/z* = 766.2969 (calc. 766.2971, error = -0.2 ppm).

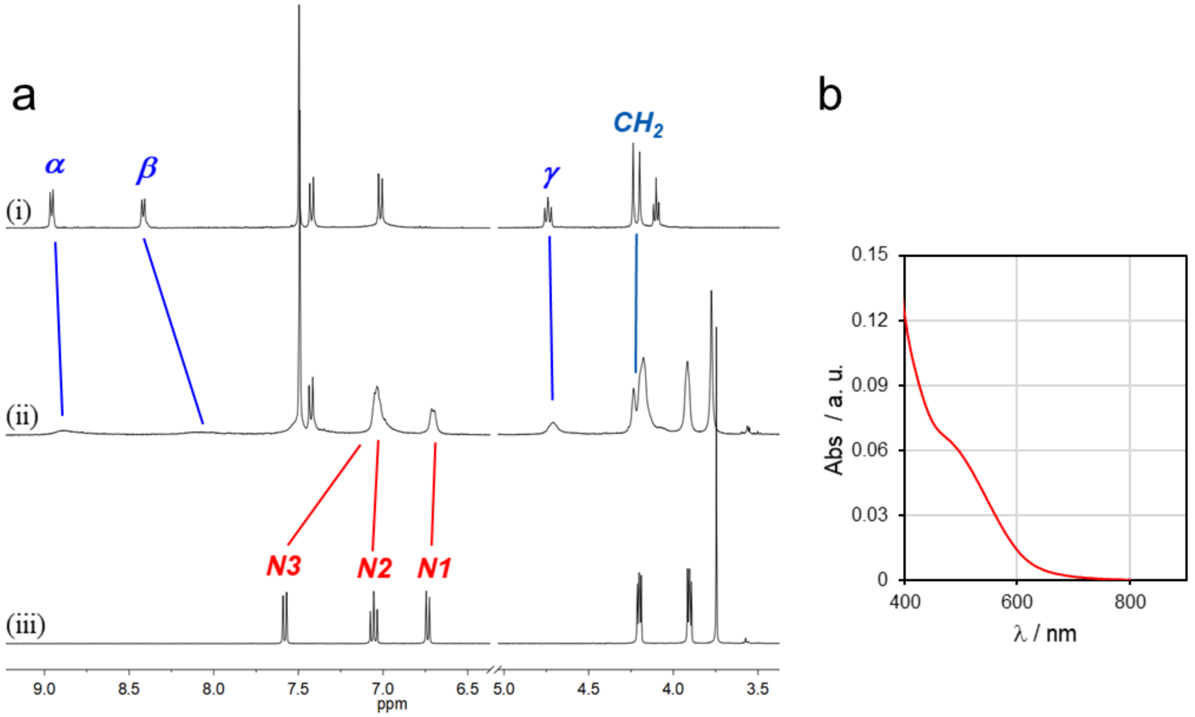

Figure S3. a) Partial ^{1}H NMR spectra (400 MHz, CD$_3$CN) of (i) guest [**1**·H$_2$][PF$_6$]$_4$, (ii) an equimolar mixture of **DN32C8** and [**1**·H$_2$][PF$_6$]$_4$ (2 × 10^{-3} M), and (iii) macrocycle **DN32C8**. b) UV-visible spectrum (MeCN, 25 ºC) of a 1:1 mixture of guest and host (5 × 10^{-3} M).

On the other hand, K_{asso} for pseudorotaxane [**1**·H_2⊂**DN32C8**][PF_6]$_4$ was determined by titration of macrocycle **DN32C8** with thread [**1**·H_2][PF_6]$_4$ in CD_3CN at 298 K, and probed by ^{1}H NMR spectroscopy (Figure S4).

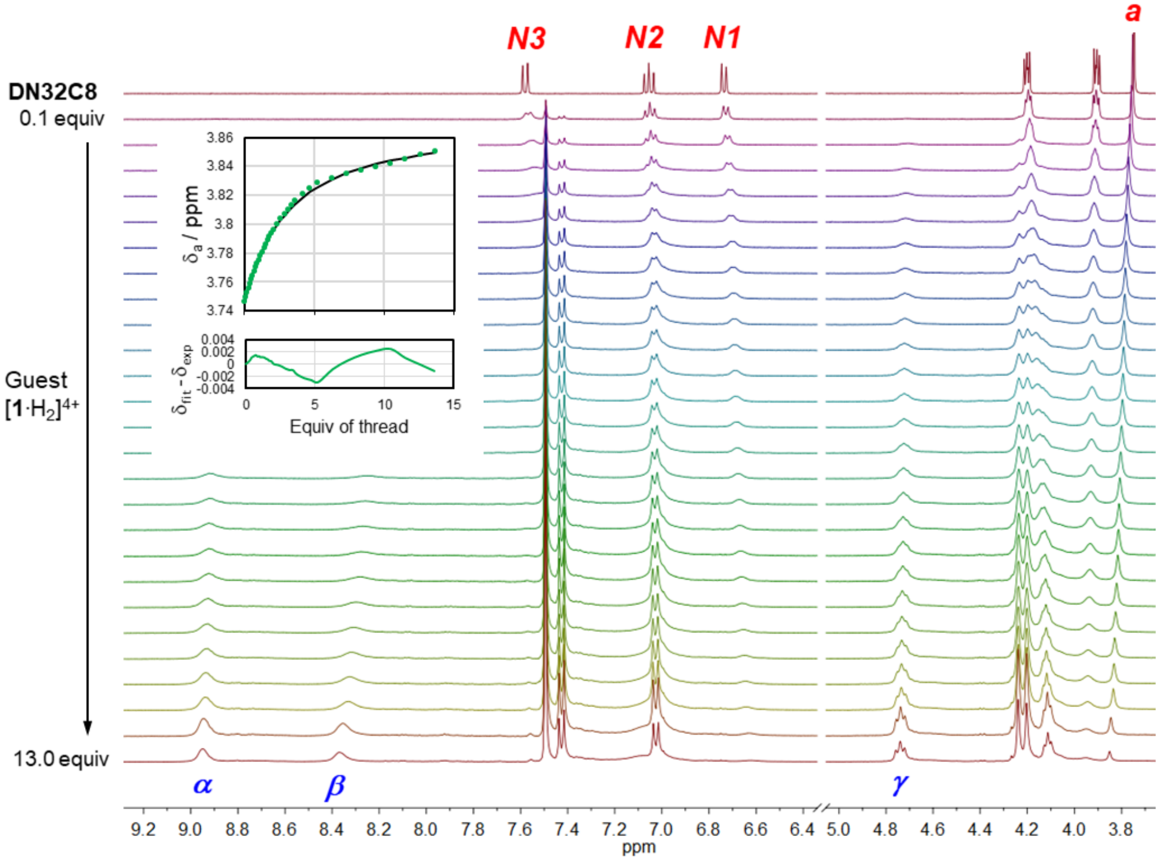

Figure S4. **DN32C8** (2 × 10^{-3} M) titration with thread [**1**·H_2][PF_6]$_4$ in CD_3CN (4 × 10^{-2} M); experiment monitored by ^{1}H NMR spectroscopy (400 MHz, CD_3CN) at 298 K. The insert contains a selected titration isotherm plotted for proton *a* from **DN32C8**; experimental data (green dots), fitted data (black line). The residual plot is also included.

The resulting isotherms were fit by a nonlinear least-squares method using the BindFit platform (supramolecular.org), and using a 1:1 global fitting model (Nelder-Mead method). ΔG_{asso} was determined by applying Eq. 1. Table S1 contains the obtained thermodynamic data.

$$\text{Eq. 1} \quad \Delta G_{asso} = -RTlnK_{asso}$$

Table S1. Thermodynamic parameters corresponding to the [2]pseudorotaxane complex in CD_3CN at 298 K.

Complex	K_{asso} / M^{-1}	ΔG_{asso} / kJ mol^{-1}
[**1**·H_2⊂**DN32C8**]$^{4+}$	(1.72 ± 0.03) × 10^2	-12.8 ± 0.2

*Standard deviations were calculated from three independent experiments

Self-sorting process

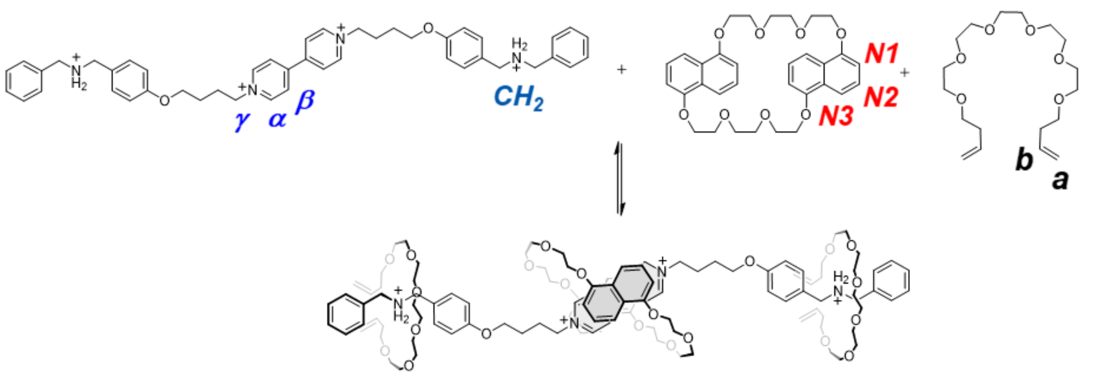

Scheme S2. Self-sorting process of thread, macrocycle and tether **C**-**22**. Proton assignment is included.

The self-sorting process of the thread molecule [**1**·H_2][PF_6]$_4$, macrocycle **DN32C8**, and the glycol precursors **C**-**21** and **C**-**22** was analysed by UV-vis and ^{1}H NMR spectroscopy in acetonitrile solutions. Figure S5 shows a representative example for the system assembled with **C**-**22**.

Mixing [**1**·H_2][PF_6]$_4$ with **DN32C8**, in a 1:1 ratio (5 × 10^{-3} M), resulted in a red solution, a color change ascribed to the formation of a charge-transfer complex with an absorbance band at 475 nm in the UV-vis spectrum (Figure S5c). In the ^{1}H NMR spectrum, only the bipyridinium resonances (α and β) showed significant changes (Figure S5a, ii), *e.g.* α moves to lower frequency and gets broader, indicating that the ring encircles only the central portion of [**1**·H_2]$^{4+}$. In a separate experiment, [**1**·H_2][PF_6]$_4$ (5 × 10^{-3} M) was mixed with two equiv of **C**-**22** and no color change was registered. In the corresponding NMR spectrum (Figure S5a, iv), only the $^+NH_2$ resonance registered a change while the pyridinium proton signals remained unaltered; this suggests that **C**-**22** wraps exclusively around the ammonium stations.

Mixing [**1**·H_2][PF_6]$_4$, **DN32C8**,and **C**-**22** in a 1:1:2 ratio resulted in a self-sorting process, *i.e.* **DN32C8** sits on the central recognition site of [**1**·H_2]$^{4+}$ while **C**-**22** interacts with the ammonium end-groups, this was confirmed by the observed matching of all chemical shifts in the ^{1}H NMR (Figure S5a, iii) with respect to the two previously analyzed systems (*vide supra*). Moreover, the UV-vis band at 475 nm was detected with no detriment (Figure S5b).

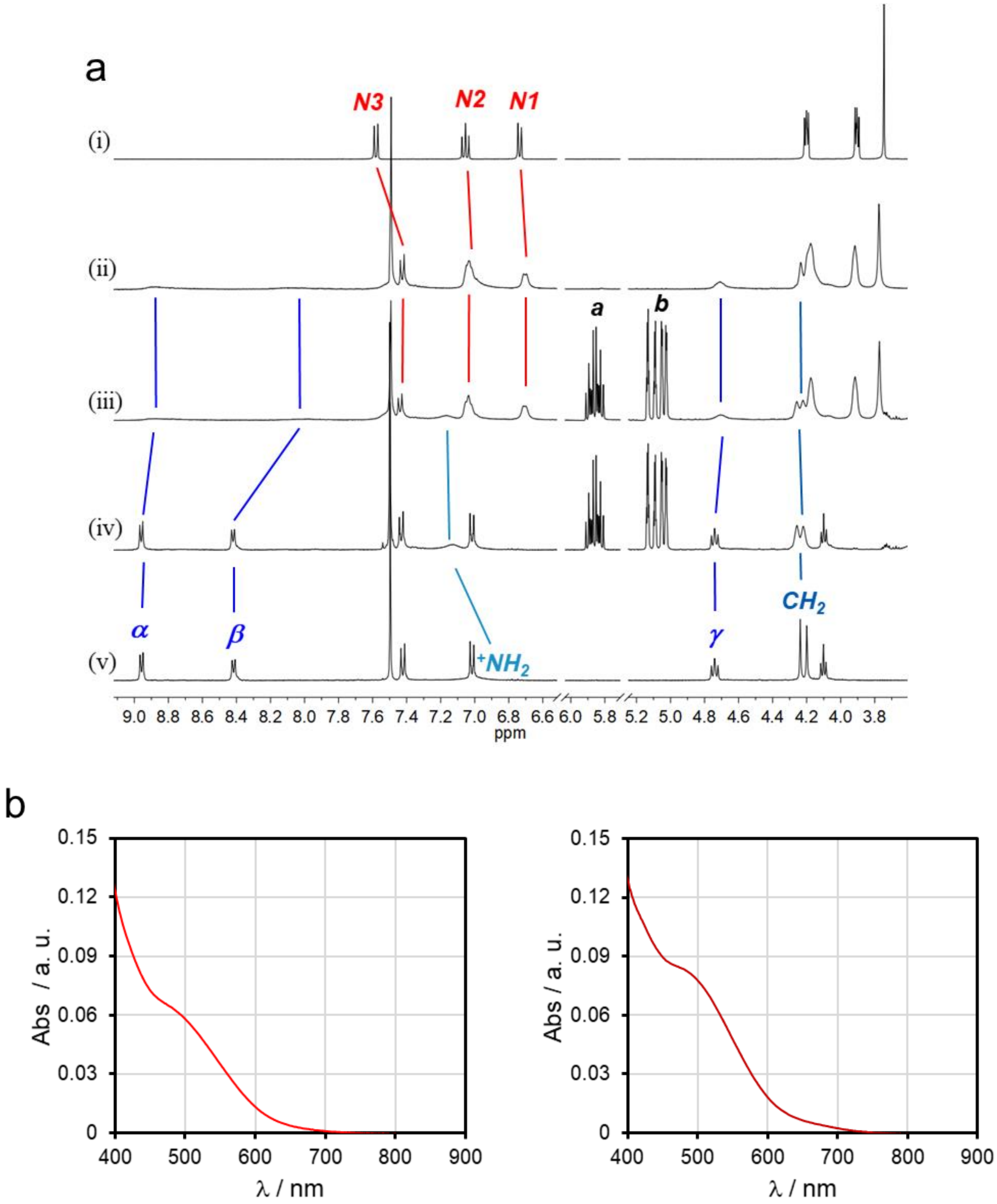

Figure S5. a) Partial ^{1}H NMR spectra (400 MHz, CD_3CN) of (i) macrocycle **DN32C8**, (ii) an equimolar mixture of thread and macrocycle **DN32C8** (5×10^{-3} M), (iii) a solution of **DN32C8**, [**1**·H_2][PF_6]$_4$, and **C-22** in a 1:1:2 ratio; [**DN32C8**] = 5×10^{-3} M, (iv) a 2:1 mixture of **C**-**22** and [**1**·H_2][PF_6]$_4$; [**C**-**22**] = 1×10^{-2} M, and (v) thread [**1**·H_2][PF_6]$_4$. b) UV-vis spectrum of a solution (MeCN) containing pseudorotaxane [**1**·H_2⊂**DN32C8**]$^{4+}$ (5×10^{-3} M) before (left) and after the addition of two equivalents of **C**-**22** (right).

Selected NMR characterization of *p*-H[4]R

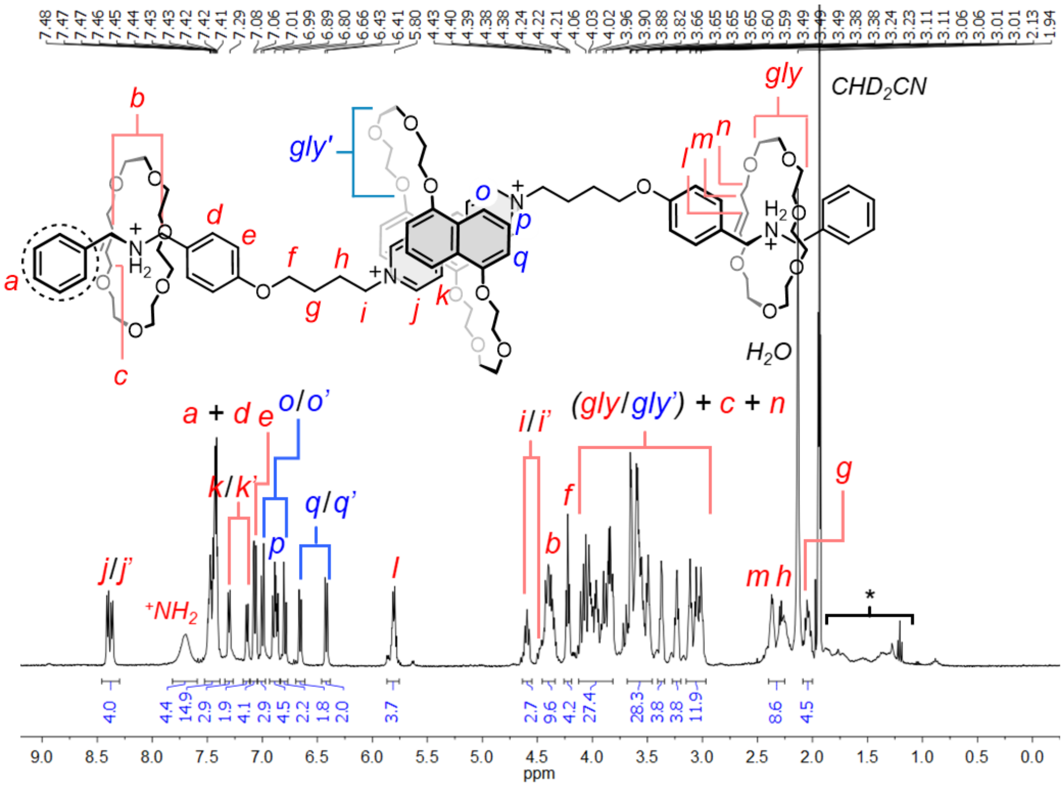

Figure S6. ^{1}H NMR spectrum (400 MHz, CD$_3$CN) of hetero[4]rotaxane ***p*-H[4]R**. * Denotes hexanes and AcOEt.

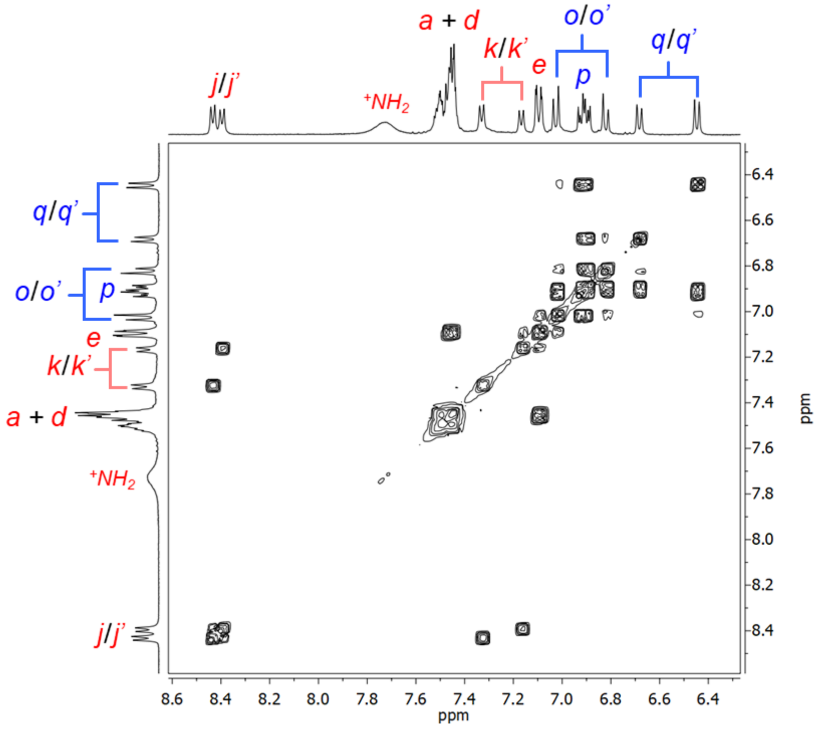

Figure S7. Partial (aromatic region) ^{1}H-^{1}H COSY NMR spectrum (400 MHz, CD$_3$CN) of ***p*-H[4]R**.

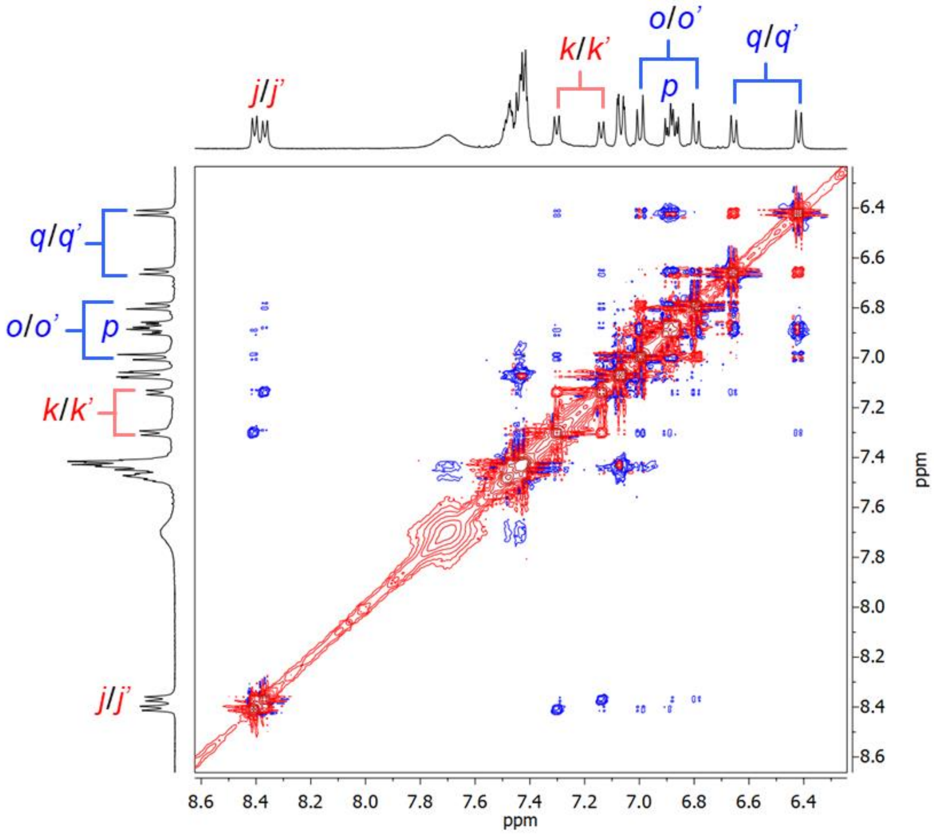

Figure S8. Partial ^{1}H-^{1}H NOESY NMR spectrum of ***p*-H[4]R**.

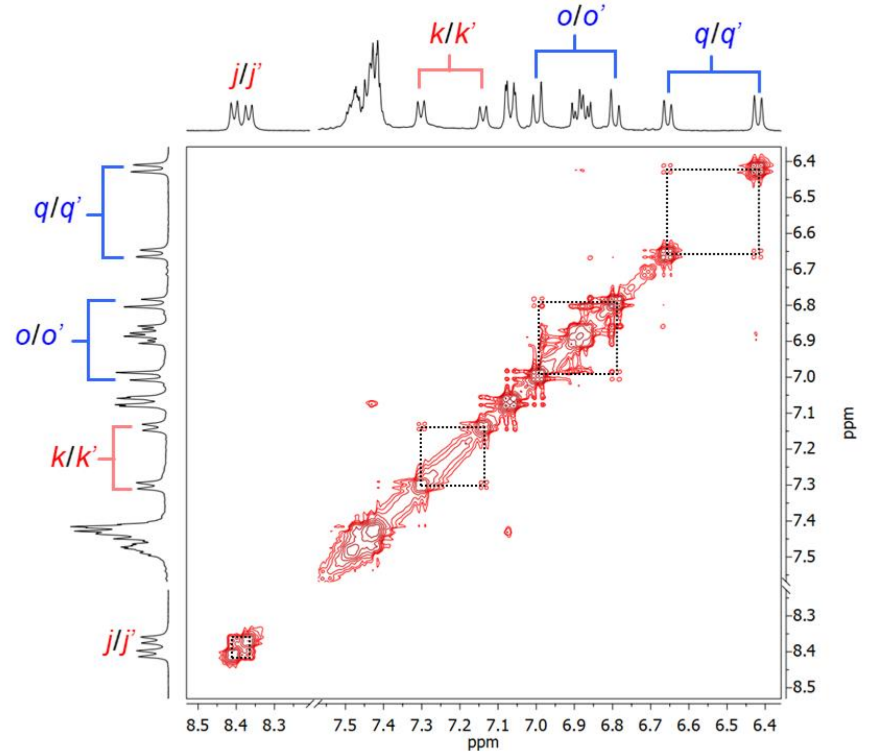

Figure S9. Partial ^{1}H-^{1}H EXSY NMR spectrum (400 MHz, CD_3CN) of ***p*-H[4]R**.

^{1}H NMR spectrum of a model [3]rotaxane

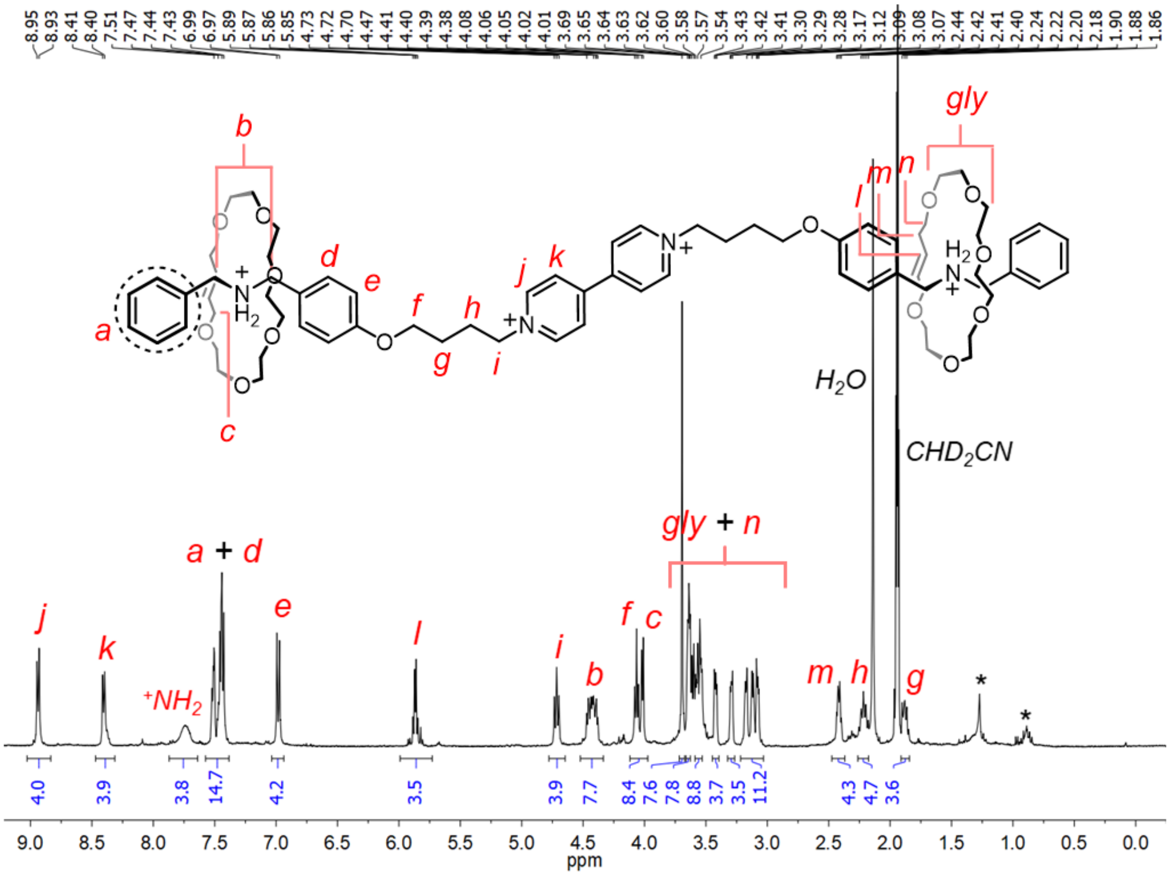

Figure S10. ^{1}H NMR spectrum (400 MHz, CD_3CN) of [3]rotaxane [**1**·H_2⊂(**21C6**)$_2$][PF_6]$_4$. * Denotes grease.

Reduction attempts on the hetero[4]rotaxane structures

Acetonitrile solutions (300 μL, 1 × 10^{-3} M) of ***p*-H[4]R** and ***t*-H[4]R** were sparged with N_2, and loaded with zinc dust (10 mg). The samples were analyzed by UV-vis spectroscopy just after adding the reducing agent and one hour later. See a representative result for ***t*-H[4]R** in Figure S11.

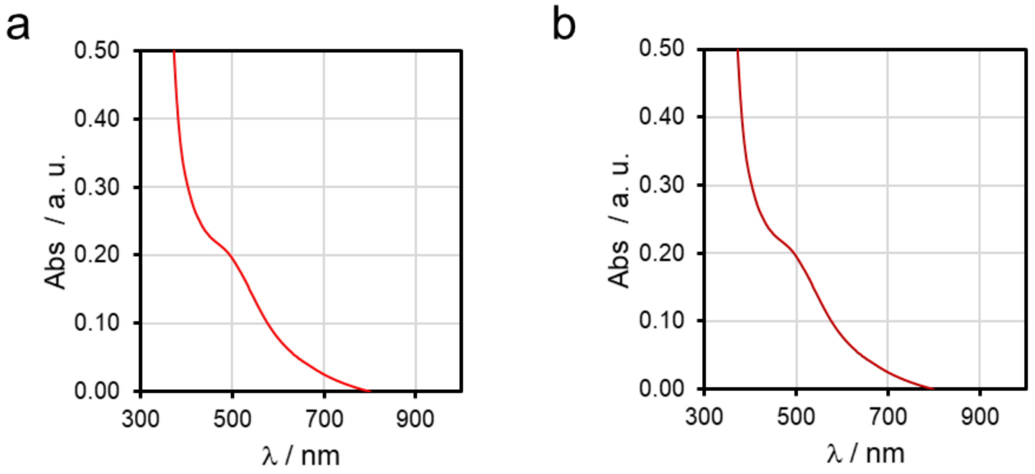

Figure S11. UV-vis spectra (CH_3CN, 25 ºC) for the attempted reduction of ***t*-H[4]R** using Zn^0. (a) Just after adding zinc dust and (b) one hour later.

Unstoppering attempts on *p*-H[4]R

Solutions of rotaxane ***p*-H[4]R** at 1 × 10^{-3} M concentration were analyzed towards three different stimuli: base, polarity, and heat.

Base trigger

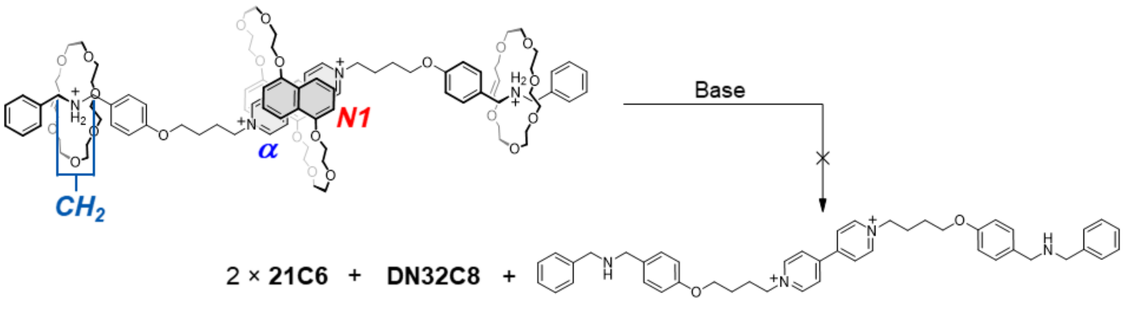

Scheme S3. Attempted unstoppering of ***p*-H[4]R** using base as a trigger.

An alkaline solution (1 M, 1 equiv, $NaOH_{(aq)}$) was added to a CD_3CN solution of ***p*-H[4]R** and stirred for 10 min at room temperature. The ^1H NMR data collected at this point is shown in Figure S12.

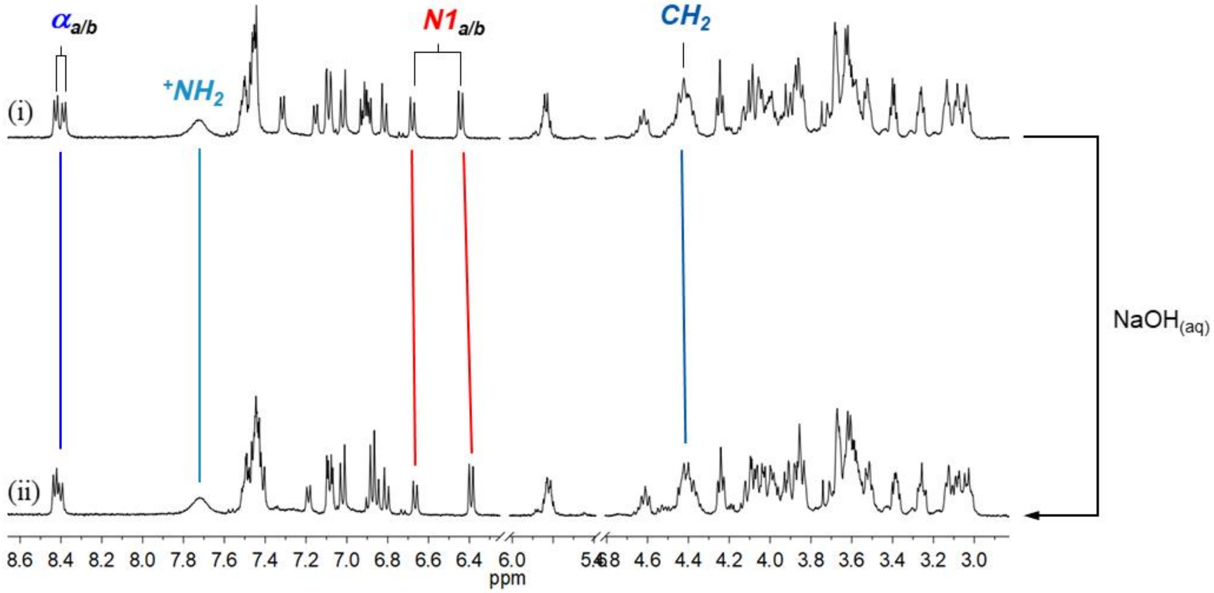

Figure S12. Partial ^1H NMR spectra (400 MHz, CD_3CN) of ***p*-H[4]R** (i) before and (ii) after the addition of one equivalent of base (1 M $NaOH_{(aq)}$). Selected resonances have been labeled.

From the experiment, it is evident that the $^{+}NH_2$ resonance is present after the addition of base. The chemical shifts of the remaining signals suggest that the hetero[4]rotaxane stays (in both isomeric forms) unaltered in solution. Only slight changes were noticed that might be attributed to the presence of water and Na^+ ions coming from the added alkaline solution.

UV-vis spectroscopy (see band at 490 nm) and HRMS (*m/z* = 463.0509, 665.6573, and 1070.7871) further confirmed that ***p*-H[4]R** remains in solution, see Figure S13.

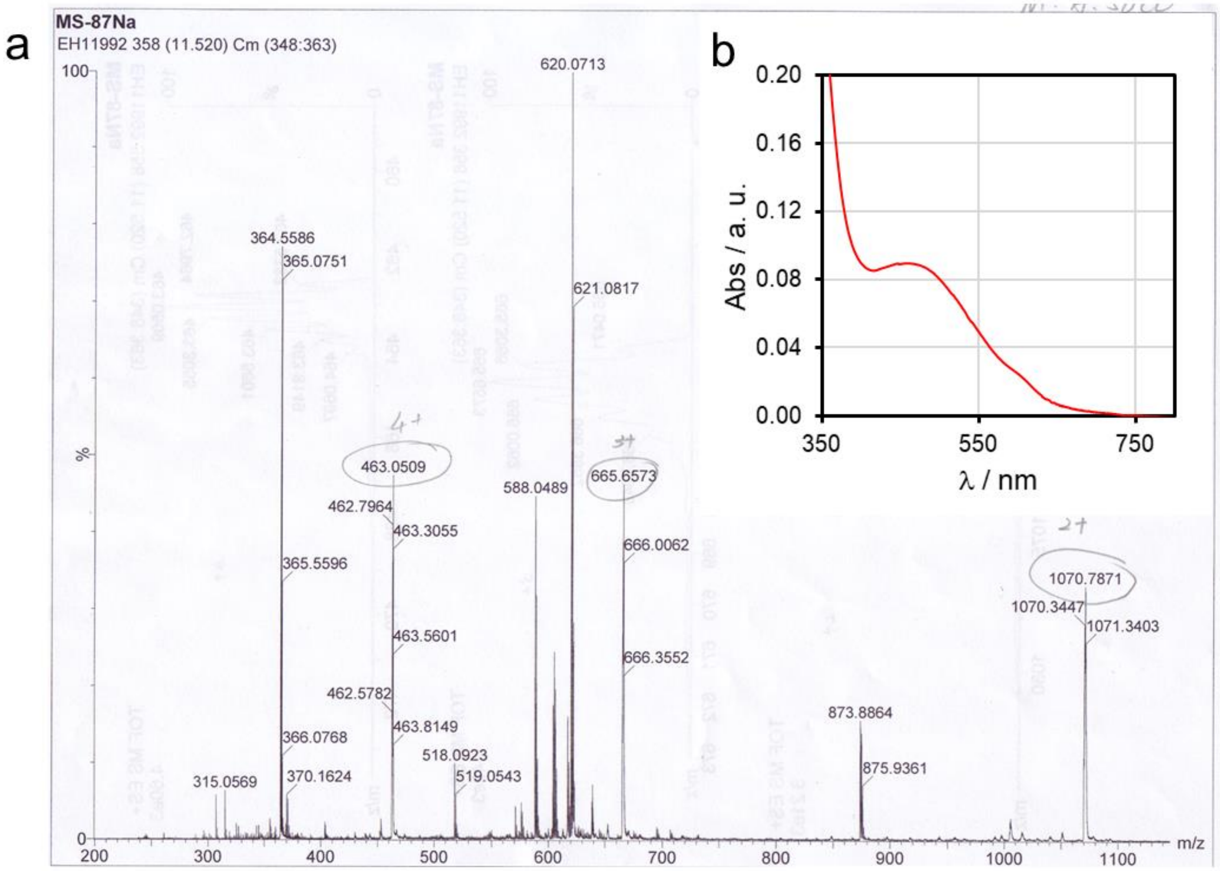

Figure S13. a) HRMS for a sample of ***p*-H[4]R** treated with one equiv of $NaOH_{(aq)}$. Ions [**1** + **DN32C8** + (2 × **21C6**) + 2H]$^{4+}$, [**1** + **DN32C8** + (2 × **21C6**) + 2H + PF_6]$^{3+}$, and [**1** + **DN32C8** + (2 × **21C6**) + 2H + $2PF_6$]$^{2+}$ are detected at *m/z* 463.0509, 665.6573, and 1070.7871, respectively. The free components as [**DN32C8** + Na]$^+$ (calc. m/z = 571.2302), [**1**]$^{2+}$ (calc. m/z = 346.2040), and [**21C6** + Na]$^+$ (calc. m/z = 327.1778) were not observed. Peak at m/z = 364.5586 might correspond to a fragmented portion of ***p*-H[4]R**, *i.e.* a viologen containing the oxybutylene chain and the mechanical stopper only on one *N*-position (calc. m/z = 364.7171). Peaks at m/z = 588.0489, m/z = 620.0713, and m/z = 873.8864 do not correspond to any envisioned fragmentation product. b) UV-vis spectrum (MeCN) of ***p*-H[4]R** treated with one equiv of base.

Polarity and temperature effect

When a solution of rotaxane ***p*-H[4]R** was prepared in DMSO-d_6 and analyzed at room temperature over a period of one month, no changes were observed. A representative ^{1}H spectrum is shown in Figure S14. As observed in CD_3CN, two isomers are distinguished in DMSO-d_6; however, their relative concentrations correspond to 2:1 according to the ^{1}H NMR integrals.

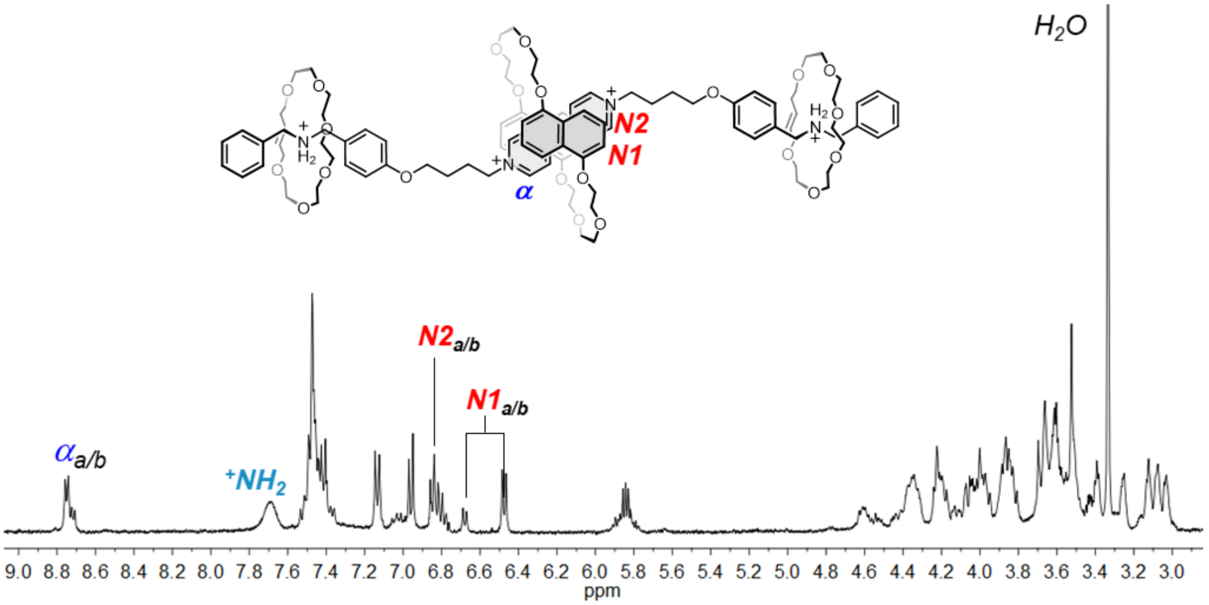

Figure S14. ^{1}H NMR (400 MHz, DMSO-d_6) of rotaxane ***p*-H[4]R** after 1 month of storage.

Heating the DMSO-d_6 sample did not produce significant effects. Elevated temperatures only caused faster isomer interconversion as suggested by the ^{1}H NMR spectra collected from 25 ºC to 125 ºC (Figure S15).

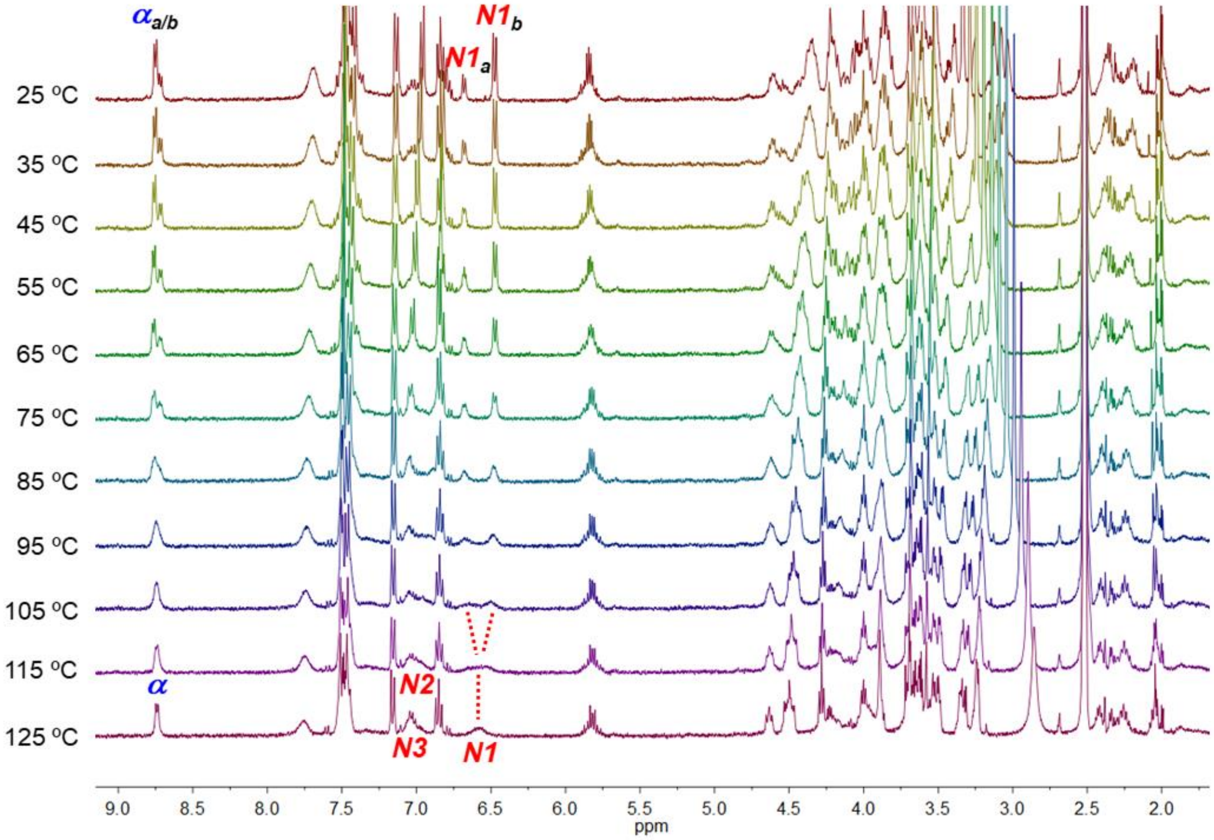

Figure S15. VT-NMR spectra (400 MHz, DMSO-d_6) for compound ***p*-H[4]R**. Selected resonances are labeled.

Isomer interconversion process

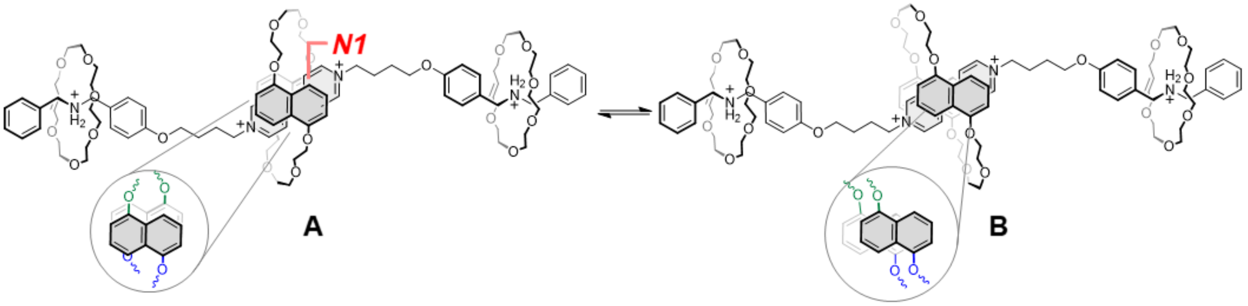

Scheme S4. Isomer interconversion.

To estimate the energy barriers between isomers **A** and **B** (interconverted by the rotation of one naphthalene motif (Np) along the RO–Np–OR axis), we employed the coalescence temperature method.[4] Resonances $N1_a$ and $N1_b$ were monitored by ^{1}H NMR in DMSO-d_6 from 25 to 125 ºC (Figure S16).

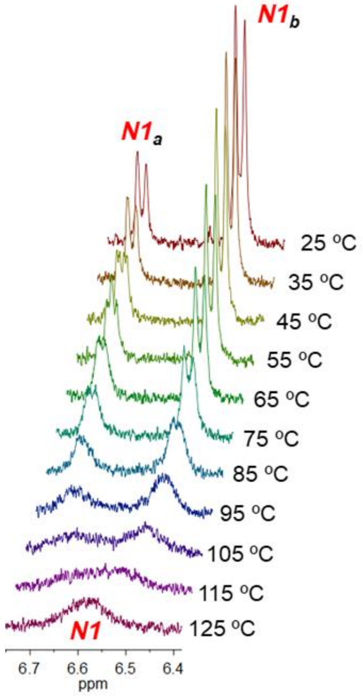

Figure S16. Partial VT-NMR spectra (400 MHz, DMSO-d_6) of compound ***p*-H[4]R**.

At the coalescence temperature (115 ºC) the exchange rate of the isomers is given by

$$k = \frac{\pi(\Delta\nu)}{\sqrt{2}}$$

where k is the exchange rate constant and $\Delta\nu$ is the limiting chemical shift between the exchanging resonances. For our system a k of 110 s^{-1} was found. The values of the free activation energies for each isomer were obtained using Eyring's equation:

$$\Delta G^{\neq}_{\mathbf{A}} = RT_{\mathrm{c}} \ln\left[\frac{k_{\mathrm{B}}}{\pi h}\left(\frac{T_{\mathrm{c}}}{\delta\nu}\right)\left(\frac{\mathrm{X}}{1-\Delta \mathrm{P}}\right)\right]$$

$$\Delta G^{\neq}_{\mathbf{B}} = RT_{\mathrm{c}} \ln\left[\frac{k_{\mathrm{B}}}{\pi h}\left(\frac{T_{\mathrm{c}}}{\delta\nu}\right)\left(\frac{\mathrm{X}}{1+\Delta \mathrm{P}}\right)\right]$$

where T_{c} is the coalesce temperature (388 K), R is the gas constant (1.986 × 10^{-3} kcal mol^{-1}), h is Planck's constant (1.584 × 10^{-37} kcal s), k_{B} is the Boltzmann's constant (3.299 × 10^{-27} kcal K^{-1}), $\delta\nu$ is the limiting chemical shift for the isomer signals at slow exchange (80 Hz at 298 K), and ΔP is the mole fraction difference between isomers **A** ($\chi_{\mathbf{A}}$ = 0.66) and **B** ($\chi_{\mathbf{B}}$ = 0.33); ΔP = 0.33. Parameter X was obtained via iteration of

$$\Delta \mathrm{P} = \left(\frac{\mathrm{X}^2 - 2}{3}\right)^{3/2} \times \frac{1}{\mathrm{X}}$$

(see Figure S17a for reference), X = 2.09 at ΔP = 0.33. The interconversion found barrier energies correspond to 19.5 kcal mol^{-1} and 19.0 kcal mol^{-1}, which implies a free energy difference (ΔG) of 0.5 kcal mol^{-1} (Figure S17b).

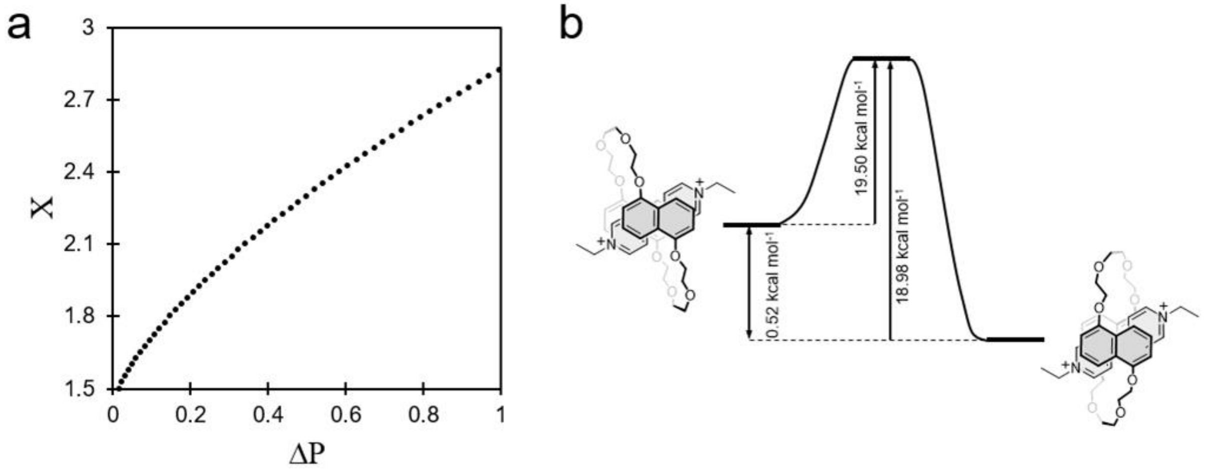

Figure S17. (a) Plot showing the iteration of parameter X. (b) Isomer representation and their relative energies.

Although other dynamic processes occurring within the hetero[4]rotaxanes cannot be excluded (such as short-rage shuttling and pirouetting), we rationalize that the *anti*/*syn* isomerization would be the only solvent-dependent process yielding different ratios of **A** and **B** in CD_3CN (1:1) and DMSO-d_6 (2:1).

Further computations demonstrated that the *anti*-**DN32C8** ring renders a more stable interlocked species when compared to the corresponding *syn* isomer. The computed energy difference for both isomers, using a **DN32C8**/*n*-butyl viologen system as a model, corresponds to 0.94 kcal mol^{-1}, in close agreement with the experimental observations in DMSO. Computed data in MeCN, as explicit solvent, produced similar results (1.43 kcal mol^{-1}), see Figure S18.

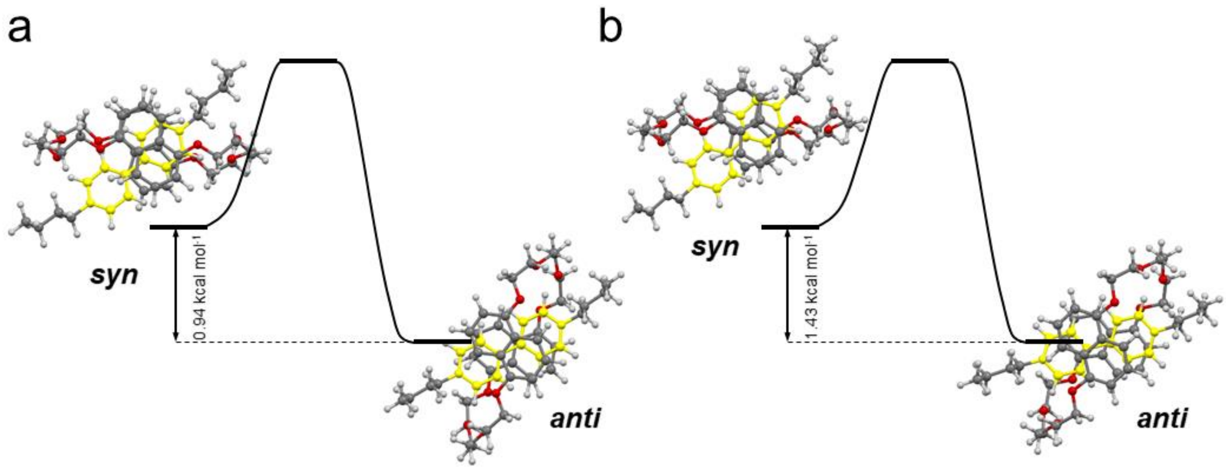

Figure S18. Computed syn/anti isomers and their relative energies in (a) DMSO and (b) MeCN. The viologen motif, as part of an *n*-butyl viologen, is represented in yellow.

These results were obtained with the Gaussian suite of program G016.revB01.[5] Geometries have been computed with the range separated dispersion corrected (wB97xD)[6] xc functional. Basis set for all atoms was 6-31g(d).[7] All structures were checked for being minima by computing the Hessian matrix at the same level of theory. All integrals were computed using the “Ultrafine” integration grid. Calculations were performed in solvent (MeCN and DMSO) using the SCRF (Self Consistent Reaction Field) approach for continuum solvent model simulation included in the Gaussian suite of programs (see data in Table S2–Table S4).[8] Reported SCXRD geometries[1,9] were used as input guesses for the optimization, increasing all the bond distances involving hydrogen atoms to 1.07 Å and adding the *n*-butyl side chains to the N and N’ positions of the viologen moiety.

Table S2. Cartesian coordinates of the *anti*-**DN32C8**/n-butyl viologen complex in its two most stable conformations (MeCN).

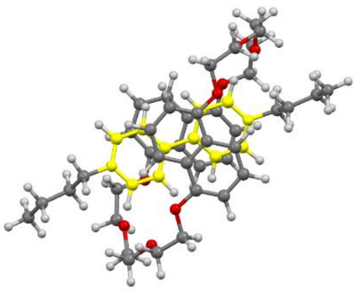<br>Electronic Energy (EE) -2652.861492 h<br>EE + Thermal Free Energy Correction<br>-2651.884724 h	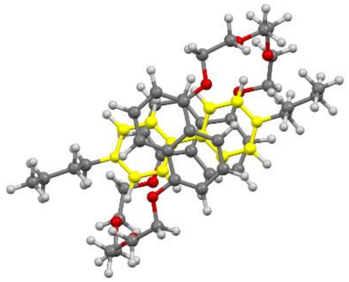<br>Electronic Energy (EE) -2652.861642 h<br>EE + Thermal Free Energy Correction<br>-2651.884901 h
Anti-**DN32C8** *anti*-*n*-But 6-31gd wb97xd MeCN	*Anti*-**DN32C8** *anti*-*n*-But 6-31gd wb97xd MeCN
N 0.1572498292 0.0373777558 0.0117002241	N 0.1642930091 0.2364004674 0.3268869638
N -0.5778184617 0.064736723 6.9664033549	N -0.5537298211 -0.0377073119 7.2755024561
C 1.2452129634 0.000520835 0.7988067431	C 1.2512324514 0.174631501 1.1144201842
O -2.272839328 3.0865478212 6.4911605456	O -2.2516134414 3.1108266859 6.3023080724
O -3.7176108331 1.1713793479 8.1346072733	O -3.6892232369 1.222172411 8.0868375051
O -4.3164742104 -1.3322824292 6.9756903236	O -4.3743070733 -1.2868354011 6.9998321941
O -2.4293149684 -2.9766955836 5.3833399891	O -2.4880323142 -2.9788708441 5.4122011334
O 2.6287647824 -2.5797712025 2.2849035904	O 2.4527937274 -2.5486258293 2.1178278911
O 4.2665921336 -0.4295605644 1.0702868088	O 4.1665429578 -0.487764853 0.7493297953
O 3.2962414476 2.0790185926 0.2721702525	O 3.1415552194 1.9692549885 -0.2149189132
O 1.503739678 3.6826725916 1.9261800083	O 1.4184176671 3.5226041991 1.6155005201
C 0.2609505491 3.6582752494 2.4637859815	C 0.1897827128 3.5554091912 2.1896187884
C -0.9096567095 3.6767605701 1.7407144957	C -0.9990879757 3.6097003426 1.4990213041
C -2.1493858855 3.5451089484 2.4086912232	C -2.2240290476 3.5226618686 2.20138407
C -2.2161295934 3.4065813113 3.7700310859	C -2.2593958473 3.3926367047 3.5649363102
C -1.0234539432 3.4487545384 4.5385027974	C -1.0472048445 3.4068657798 4.3031004959
C 0.2321127448 3.5951813021 3.8948946218	C 0.1955155063 3.5104137635 3.6228649802
C 1.4200124703 3.6858691931 4.6657729621	C 1.4046207404 3.5857343535 4.3620230782
C 1.3444297543 3.6606908057 6.0330817032	C 1.3614968907 3.6006669405 5.7310479836
C 0.1078725244 3.4940638562 6.6992447481	C 0.1389003511 3.4813876311 6.4324227786
C -1.0491269404 3.350308679 5.9673217756	C -1.0392150991 3.3386328063 5.7356637383
C -2.4640991407 3.1800711856 7.8946962719	C -2.3695119643 3.1716496798 7.7169026176
C -3.8094774706 2.5783814609 8.2306633962	C -3.725823007 2.6347443028 8.1186178899
C -4.9502649404 0.514966689 8.3474278506	C -4.9469484102 0.619688477 8.3135631838
C -4.7459942601 -0.9817551634 8.2761822661	C -4.7959140366 -0.886225548 8.2894587993
C -4.3858635855 -2.721159736 6.7171807521	C -4.4175008882 -2.6863849493 6.794122867
C -3.8490067172 -2.9939188228 5.3298835302	C -3.9104711627 -2.9950271885 5.4015342299
C -1.7282730922 -3.039566917 4.2231838458	C -1.8244781068 -3.0312873111 4.228306208
C -2.2819021708 -3.0601333335 2.9644891291	C -2.4233235698 -3.0474299466 2.9893734848
C -1.4373622001 -3.0499307919 1.8270942285	C -1.6233556395 -3.021884891 1.8218592769
C -0.0754443453 -2.9786373032 1.9487128368	C -0.2579061762 -2.9450356212 1.8936633339
C 0.5141286771 -2.9449712704 3.240454487	C 0.3795131003 -2.924978059 3.1608691347
C -0.3063475762 -3.0324537473 4.3931973314	C -0.3962385178 -3.0204808612 4.3453725766
C 0.2791936922 -3.0797166857 5.6822767914	C 0.242165614 -3.069214347 5.6103470697
C 1.6404181397 -3.0066476128 5.8145550053	C 1.6073391928 -2.9906139594 5.6851563219
C 2.4777213921 -2.848257389 4.6871984152	C 2.3995102059 -2.8256967347 4.5248373559
C 1.9269422481 -2.798442873 3.4256780386	C 1.7995002708 -2.7770528839 3.2867520617
C 4.0365643718 -2.4020925457 2.3753699723	C 3.8678864271 -2.3997390255 2.1460444516
C 4.5410676431 -1.8175012621 1.0763811099	C 4.3435833946 -1.8912537081 0.8018630014
C 4.7676742521 0.2294180849 -0.0760749611	C 4.5908961964 0.0851598536 -0.4734619343
C 4.6535876097 1.7252031596 0.1126912468	C 4.4934924698 1.5921402953 -0.3781352933
C 3.0674877764 3.4669026269 0.1495101103	C 2.9473081605 3.3686899366 -0.2038536498
C 1.6308919184 3.7543044115 0.5156091429	C 1.5221576082 3.6663205199 0.2062510581

C	0.3351097673	-0.1394743112	-1.4497959056	C	0.3575205794	0.118048565	-1.1350062031
C	-1.061937131	0.2471570452	0.5435578895	C	-1.0661707999	0.3294787322	0.8625143909
C	-1.221080275	0.3781258792	1.9041314991	C	-1.2361134817	0.35953879	2.2276108664
C	-0.110894347	0.3040708806	2.7532741769	C	-0.1224860308	0.3049510212	3.0724655185
C	1.1420123764	0.1556835218	2.1613543832	C	1.1350088227	0.2301214909	2.4802414281
C	-0.267946975	0.2819838187	4.2280544527	C	-0.2659673605	0.2462148564	4.5436255373
C	-1.5245491424	0.0899505975	4.8030118128	C	-1.5127325167	0.0126069228	5.1181942752
C	-1.6561075998	-0.0207250204	6.16567338	C	-1.6291883847	-0.1414649216	6.4749130552
C	0.6472255707	0.2628074616	6.4476100952	C	0.6634623254	0.2064953148	6.7592067065
C	0.8302262672	0.3757041649	5.0873466273	C	0.8344396951	0.3496543233	5.3998952063
C	-0.7662303279	-0.1277074856	8.4218624197	C	-0.7502947682	-0.2757448954	8.7227062114
H	3.741301846	4.0345362386	0.8067901047	H	3.6414182789	3.8530039294	0.4972229294
H	3.2450128787	3.7911772579	-0.886620267	H	3.1277718116	3.7845779094	-1.2053607568
H	5.2323737257	2.0411991764	0.9924623957	H	5.0915321241	1.9507505562	0.47197436
H	5.081642572	2.2163470221	-0.773630869	H	4.9091533688	2.0286350482	-1.2982300651
H	5.8262996712	-0.0238101898	-0.2325780229	H	5.6357956349	-0.1870085431	-0.680736706
H	4.2105272482	-0.0824638887	-0.973911075	H	3.9729018671	-0.2845605686	-1.3060311935
H	5.6253858337	-1.985895662	1.0111973149	H	5.4091823345	-2.1352687519	0.6890838749
H	4.0655358296	-2.3181814023	0.2205517527	H	3.7930527499	-2.3893079582	-0.0093561487
H	4.5169571144	-3.3665027537	2.576646025	H	4.3319612705	-3.3664243397	2.3727493489
H	4.2870146639	-1.7056921168	3.1835055405	H	4.1649077186	-1.6771371496	2.9150998383
H	3.5472968523	-2.7722514971	4.8379264927	H	3.4736240164	-2.7407580101	4.6338594188
H	0.5615341886	-2.9485293404	1.072430181	H	0.3461194328	-2.9058743189	0.9951036233
H	-1.8929577625	-3.0817341646	0.8422129393	H	-2.1156575986	-3.0469105098	0.854339779
H	-3.3555099797	-3.0828102536	2.8214306079	H	-3.5009303593	-3.0786886153	2.8847631993
H	0.985398346	3.0101700111	0.0361468977	H	0.8495609705	2.9683637135	-0.306956232
H	1.337740682	4.7503488646	0.1623678392	H	1.2474407211	4.6875868506	-0.0833779486
H	0.0852876783	0.3311169837	8.9269376386	H	0.1135128261	0.1340242738	9.2482043122
H	-1.675754396	0.4118930421	8.6918220091	H	-1.6408889358	0.288817793	9.0104507626
H	-3.0599753176	3.5422647357	1.817475189	H	-3.1496041247	3.5453710546	1.634566468
H	-0.90482617	3.7664866903	0.6611636325	H	-1.0208309066	3.6941213053	0.4191505541
H	2.3727153819	3.7966790569	4.1622792054	H	2.3468638029	3.660566905	3.8327727682
H	-3.1682255457	3.2902213816	4.2733109816	H	-3.2017400818	3.3099137436	4.0929124228
H	2.2464462262	3.7564681429	6.6293760373	H	2.2802503447	3.6907770366	6.3021620152
H	0.0977272016	3.4684272687	7.7822762996	H	0.1578086554	3.4838027618	7.5154333236
H	-2.4273038721	4.231188421	8.2040350025	H	-2.2548555689	4.2093367595	8.051388646
H	-1.6840602886	2.6345564017	8.4363297361	H	-1.5940774696	2.5661277837	8.1998707362
H	-4.5749452463	2.9646994039	7.5431002095	H	-4.4966691538	3.0197119391	7.4364404391
H	-4.0863617694	2.8730527023	9.2531049731	H	-3.961967432	2.9842270876	9.1338408986
H	-5.3570274114	0.7695682279	9.3370558352	H	-5.3491706274	0.9195099393	9.2922986619
H	-5.6796076012	0.8254986092	7.5857126568	H	-5.6611046556	0.9316489482	7.5381948639
H	-5.7010300239	-1.4725758364	8.511336142	H	-5.7661789229	-1.3399908009	8.5355380039
H	-4.0061035522	-1.3058881495	9.023761498	H	-4.0679402575	-1.2113756813	9.0491945105
H	-3.8084833662	-3.2905210945	7.4599576197	H	-3.8113199653	-3.2166677573	7.5428858495
H	-5.4311764554	-3.0570751919	6.7671085658	H	-5.4533584635	-3.0425019416	6.881734416
H	-4.2193616389	-2.221281951	4.645264592	H	-4.2997396736	-2.2376551846	4.7107587424
H	-4.193866917	-3.9730161376	4.9770752738	H	-4.2639690595	-3.9815964149	5.0800254363
H	-0.3620356662	-3.192370846	6.5464635221	H	-0.3608956806	-3.1783672132	6.5033159213
H	2.0958277357	-3.0578032263	6.7989315025	H	2.1023505749	-3.0373006687	6.6503544812
H	-0.6443930761	-0.368085791	-1.8719220444	H	1.2513940064	0.6991232172	-1.3761108625
H	0.975408756	-1.0146954652	-1.5843201525	H	-0.502009514	0.5830752553	-1.6209695135
H	-1.895054736	0.2884969518	-0.1452491343	H	-1.8978319953	0.3754733471	0.1719273852
H	-2.2229493733	0.5412394997	2.2743731184	H	-2.2458806908	0.4478391727	2.6004569844
H	-2.4225094612	-0.0164334539	4.2108185464	H	-2.4135008675	-0.1015778973	4.5320483524
H	-2.6136123478	-0.1954556235	6.6395294691	H	-2.581170622	-0.3622724403	6.9347057736
H	1.4686902388	0.3244529136	7.1490441908	H	1.4854492502	0.2793014888	7.4589983632
H	1.8355955963	0.5450026423	4.7291156551	H	1.8325875997	0.5562451979	5.0419788601
H	2.0616229739	0.0891273406	2.7254295566	H	2.0491411153	0.1615111475	3.0533061578
H	2.201456161	-0.1802395558	0.332797165	H	2.2164185878	0.0699134633	0.6421413152
C	-0.9072367133	-1.6040661576	8.7726672478	C	-0.9322928848	-1.7637100181	9.0049419151
H	-1.7096192771	-2.0373204753	8.1601870395	H	-1.7300400657	-2.154948331	8.3588395164
H	0.0184686739	-2.1340141325	8.5207061526	H	-0.0125883347	-2.3002171931	8.742061133

C	-1.2274556217	-1.7953392499	10.2549912083	C	-1.2884844825	-2.0200392614	10.4688307882
H	-0.4072965932	-1.3897347651	10.8599060709	H	-0.4828238452	-1.6447514809	11.1114089439
H	-2.1216020339	-1.2134139749	10.5138013107	H	-2.1881764795	-1.4476563771	10.7292871801
C	-1.4523819476	-3.2650390989	10.6027085433	C	-1.5258478629	-3.5029055567	10.7453621291
H	-0.565055595	-3.8637874103	10.3693124837	H	-0.6341901004	-4.0940896017	10.5094325095
H	-1.6725642872	-3.387644526	11.667430255	H	-1.7739719854	-3.6706312365	11.7976908212
H	-2.2941309638	-3.6764418213	10.0339145467	H	-2.353773112	-3.8857978248	10.1379045158
C	0.9485646538	1.1033797525	-2.0856917973	C	0.5138465501	-1.3437870397	-1.5417806245
H	0.2554754746	1.9458053239	-1.9669467172	H	1.3314445905	-1.7930980227	-0.962782102
H	1.8723780769	1.3618167596	-1.5538345329	H	-0.4019194016	-1.8914229816	-1.2866699428
C	1.2550161507	0.8891685012	-3.5676661492	C	0.8112772719	-1.4828825492	-3.0343840921
H	0.3324115728	0.6338856464	-4.1032672172	H	1.7191302815	-0.9157015313	-3.2755477451
H	1.9310584942	0.0318734777	-3.6771842803	H	-0.0047810328	-1.0323140174	-3.6125303559
C	1.8921290742	2.1285626237	-4.1931929566	C	0.9923572552	-2.9426287412	-3.4449153253
H	2.1169226742	1.963871202	-5.2512963227	H	1.2064119761	-3.0252455437	-4.5147588264
H	1.2228678819	2.9930110139	-4.120387787	H	1.8228405743	-3.4023795379	-2.8974077854
H	2.8283345196	2.3849105581	-3.6840316844	H	0.0881212874	-3.5245748831	-3.2349870511

Table S3. Cartesian coordinates of the *anti*-**DN32C8**/n-butyl viologen complex in its most stable conformation (DMSO).

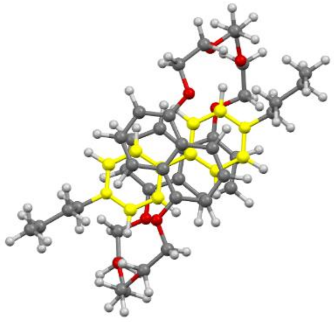

Electronic Energy (EE) -2652.862832 h
EE + Thermal Free Energy Correction -2651.886023 h

Anti-**DN32C8** *anti*-*n*-But 6-31gd wb97xd DMSO

N	0.161978754	0.235581277	0.3246550502
N	-0.5534344126	-0.0384056118	7.2735864645
C	1.2492991517	0.1736022631	1.1116533601
O	-2.2507031005	3.1117381498	6.3031414796
O	-3.6879120826	1.2225012226	8.0871529202
O	-4.3741474439	-1.2863718089	7.0008373357
O	-2.488409948	-2.9798171401	5.4132012496
O	2.4529232837	-2.5491574953	2.1197045362
O	4.1657570697	-0.4867304573	0.7517835869
O	3.1412908018	1.9696047427	-0.2137628936
O	1.4178591953	3.5233049088	1.6150739931
C	0.1894159554	3.5556235622	2.1896082648
C	-0.9996545719	3.6093982346	1.4993278135
C	-2.2243547209	3.5220049522	2.2020556405
C	-2.2592514397	3.3924037689	3.5656688169
C	-1.0468490039	3.4070535893	4.3034923708
C	0.1956516868	3.5106439222	3.6228486467
C	1.4050363793	3.5856066688	4.3615943785
C	1.3624268469	3.6001758075	5.7306406158
C	0.1400334397	3.4812399418	6.432433883
C	-1.0383798169	3.3388733097	5.7360629484
C	-2.3677854019	3.1717608847	7.7178816767
C	-3.7240029612	2.635037001	8.1201265028
C	-4.9454231356	0.6201058737	8.3152427525
C	-4.7944232556	-0.8858066632	8.2909284896

C	-4.4176827954	-2.6859184653	6.7950945444
C	-3.9108911013	-2.9947087528	5.4024439269
C	-1.8247769765	-3.0319732481	4.2293381424
C	-2.4234768969	-3.0473707986	2.9903381634
C	-1.6233437696	-3.0214477839	1.8229230086
C	-0.2578796822	-2.9450422291	1.8949062515
C	0.3794019175	-2.9256764934	3.1622234204
C	-0.3965340122	-3.0216081015	4.3465819487
C	0.2416789892	-3.0708453943	5.6116310234
C	1.6068610275	-2.9923465843	5.6867199391
C	2.3992082914	-2.8271010094	4.5265884833
C	1.7993732735	-2.7778356071	3.288424854
C	3.8678744536	-2.3987859675	2.1486518637
C	4.3437606924	-1.8900770866	0.8046399219
C	4.5918874327	0.0862897029	-0.4703661876
C	4.493599995	1.5932209272	-0.3753769356
C	2.9460770675	3.3688876346	-0.2046190042
C	1.5208605146	3.6658897855	0.2056141384
C	0.354719142	0.1175169637	-1.1373210494
C	-1.0681968577	0.3291552127	0.86084312
C	-1.2373488273	0.3593076928	2.2260642166
C	-0.1233371493	0.3043736191	3.0703737111
C	1.1338933636	0.2293296535	2.477532805
C	-0.2662971872	0.2455150266	4.5416142857
C	-1.5130497319	0.0124376374	5.1165403995
C	-1.6291772239	-0.1415928755	6.4732714753
C	0.6637351995	0.205100418	6.7569573102
C	0.8343898553	0.3482974399	5.3975615111
C	-0.7497901634	-0.2760810328	8.7208798994
H	3.640055817	3.8546753718	0.4955877564
H	3.1259070489	3.7835398544	-1.2067510713
H	5.0904713836	1.9523896778	0.4753327398
H	4.9100739033	2.0297427307	-1.2950844828
H	5.6372579244	-0.1854501239	-0.6758449981
H	3.9754575949	-0.2837561437	-1.3039205427
H	5.4095920717	-2.1333414213	0.6924139872
H	3.7939709834	-2.388633543	-0.0067769246
H	4.3328224871	-3.3649798415	2.375679285
H	4.1637048122	-1.6758403263	2.9178413018
H	3.4732678224	-2.7418746579	4.6358681188
H	0.3462124152	-2.905144213	0.9964052053
H	-2.1156169676	-3.0453916244	0.8553816808
H	-3.5010894466	-3.0777765335	2.8855461491
H	0.8485651483	2.9669797332	-0.3066392528
H	1.2452432535	4.6866753157	-0.0848456653
H	0.1145152173	0.1329695963	9.2460806308
H	-1.6397735905	0.2893347397	9.008799137
H	-3.1500742222	3.543796977	1.6354495595
H	-1.0216840044	3.6930592132	0.4194088761
H	2.3471537935	3.6599626993	3.8320533223
H	-3.201422389	3.3090897937	4.0938647165
H	2.2814225678	3.6894647624	6.3014859423
H	0.1594171243	3.4833664831	7.5154370465
H	-2.2526758871	4.2092267081	8.0528901725
H	-1.5921918538	2.5658346519	8.2000081402
H	-4.4952790778	3.020840218	7.4388950054
H	-3.9591476002	2.983783665	9.1358339298
H	-5.346532454	0.9198581642	9.2944512361
H	-5.6604288899	0.932162902	7.5406876345
H	-5.7644052036	-1.3396235694	8.53803136
H	-4.0655908925	-1.2109195192	9.0498455264
H	-3.8115208343	-3.2163260766	7.5437804974

H	-5.4536055888	-3.0417609724	6.8828930354
H	-4.2994715544	-2.2369183721	4.7117752471
H	-4.2652058753	-3.9809190233	5.0807440612
H	-0.361557212	-3.1800037957	6.5044719291
H	2.1017104566	-3.0390268616	6.6519940763
H	1.2473487998	0.7002585148	-1.37896297
H	-0.505943529	0.5808297127	-1.622841813
H	-1.9001557007	0.3755492102	0.1706734866
H	-2.2468994586	0.4478852627	2.5994095316
H	-2.4140216495	-0.1013874755	4.5306344632
H	-2.581092461	-0.3621120169	6.9333416738
H	1.4859357799	0.2774036953	7.4565195637
H	1.8326239176	0.5541164237	5.0394427757
H	2.0483819804	0.1609312759	3.0500406629
H	2.2141564973	0.0687425731	0.6387782443
C	-0.9332803278	-1.7637852151	9.003424235
H	-1.7318045676	-2.1542411693	8.3577927686
H	-0.0143165899	-2.3013463515	8.7402245478
C	-1.2890038933	-2.0193848453	10.4675477845
H	-0.4825478138	-1.6448237052	11.1095516242
H	-2.1879111684	-1.4459340683	10.7283693673
C	-1.5278325085	-3.5019245901	10.7445831852
H	-0.6369383215	-4.0941071401	10.5082318553
H	-1.7755151934	-3.6691384844	11.7971148364
H	-2.3565387536	-3.884054169	10.1377019108
C	0.5137317363	-1.3440266775	-1.5440703395
H	1.3318872016	-1.7919781308	-0.9647952968
H	-0.4012408318	-1.8931759907	-1.289450937
C	0.8120959105	-1.4824560374	-3.0365400057
H	1.7190492854	-0.9136353076	-3.2771891006
H	-0.0045123875	-1.0332864716	-3.6150009743
C	0.995991583	-2.941839842	-3.4471207467
H	1.2105853164	-3.0240020934	-4.5169108894
H	1.8271257842	-3.4000740864	-2.8993181092
H	0.0927313823	-3.5254358672	-3.2375284337

Table S4. Cartesian coordinates of the *syn*-**DN32C8**/n-butyl viologen complex in its most stable conformation in DMSO (right) and MeCN (left).

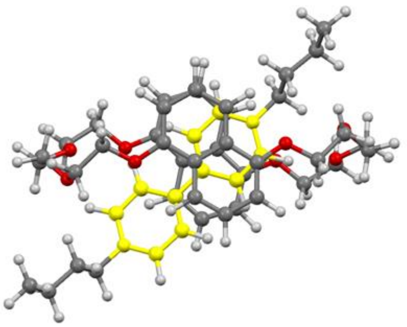<br>Electronic Energy (EE) -2652.858925 h<br>EE + Thermal Free Energy Correction -2651.882580 h	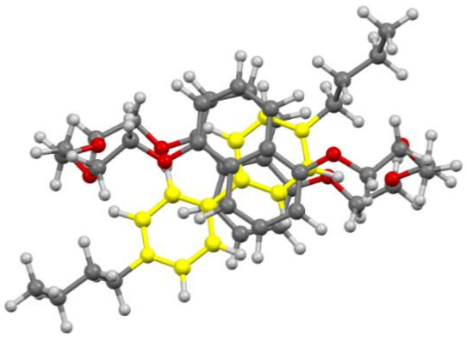<br>Electronic Energy (EE) -2652.860349 h<br>EE + Thermal Free Energy Correction -2651.884527 h
Syn-**DN32C8** *anti*-*n*-But 6-31gd wb97xd MeCN<br><br>N -1.0466991169 0.1647837058 1.7040216579<br>N 4.2089788113 -0.1448582165 -2.9211436796<br>C -1.3158184217 0.0714381789 0.3876834261<br>H -2.3575806403 0.1143276377 0.1005626806	*Syn*-**DN32C8** *anti*-*n*-But 6-31gd wb97xd DMSO<br><br>N -0.011489466 -0.0005624081 0.0174055795<br>N -0.0153813311 -0.0055949616 7.0260557537<br>C 1.1552280803 -0.0031254067 0.6902754876<br>H 2.057906744 0.0607796574 0.0980380637

C	-0.3023320122	-0.0476649131	-0.53295139	C	1.1778662773	-0.0597246139	2.0634336882
H	-0.5746553071	-0.139967892	-1.5750659633	H	2.1425233507	-0.0796378003	2.5517266151
C	1.0289295714	-0.0617011724	-0.1047773	C	-0.0239118053	-0.1060463064	2.7778612665
C	1.2715189145	0.0020129125	1.2678624801	C	-1.2135613252	-0.1390528785	2.0492174381
H	2.2717960425	0.0089927385	1.6785572033	H	-2.183687816	-0.1627472674	2.5268673922
C	0.2173611363	0.1055260343	2.1537576442	C	-1.1820182431	-0.0938899851	0.6692308819
H	0.3491241852	0.1870603788	3.2273271027	H	-2.0753224226	-0.0861370358	0.0540786618
C	2.9738153915	0.2084784151	-3.3135303701	C	1.0677402895	0.3980246647	6.341634342
H	2.841705065	0.482047925	-4.3488676171	H	1.9103322947	0.754931489	6.9130231042
C	1.9236751052	0.2218951961	-2.4240433343	C	1.0935577368	0.3582316422	4.966487907
H	0.9676125924	0.547461943	-2.8100359015	H	1.9870439272	0.7355586123	4.4892082402
C	2.1387064776	-0.1125642919	-1.0878839621	C	-0.0220644298	-0.0907046982	4.2613448187
C	3.4361114936	-0.4736266833	-0.707895562	C	-1.1366485385	-0.5021517533	5.00039974
H	3.6765918982	-0.774218728	0.3024922875	H	-2.0284983592	-0.8899709185	4.5279202706
C	4.4472741519	-0.4884909295	-1.6398256826	C	-1.1063530621	-0.4553870991	6.3749629585
H	5.46130777	-0.7747858963	-1.3940308845	H	-1.9392513477	-0.7761822181	6.9864707095
O	0.2958026633	2.9000035416	-3.0979231116	O	2.5666552305	3.0572321215	4.0353529764
O	0.6272696198	1.0014440435	-5.308210084	O	4.081830879	1.3190070269	5.8425177057
O	1.8599296954	-1.5986988153	-5.3926652663	O	3.4642415439	-1.2679465856	6.9388161883
O	1.0052346021	-3.0788458399	-2.8604205011	O	2.2230893542	-2.9232105422	4.6907090665
O	-0.2165403689	-3.1084656062	2.9617859601	O	-1.3116131505	-3.3621967977	-0.0729680693
O	-1.0198296284	-1.37429891	5.2571047233	O	-2.5899269431	-1.7894387327	-2.2669076421
O	0.0729179505	1.3056428199	5.1106409619	O	-3.3515838999	0.8921159481	-1.4604773158
O	0.2785804771	3.1558811838	2.8394297735	O	-1.8839064373	2.9039751151	0.101130289
C	-0.3199156853	3.0729100102	-1.9014673001	C	2.0682928941	3.1461202114	2.7767148887
C	-1.6837614323	3.1220500871	-1.7200710893	C	2.830386434	3.178498079	1.6307350483
H	-2.3649088518	3.069138276	-2.5604110023	H	3.9125273279	3.1792882779	1.6746192235
C	-2.2149967944	3.24802901	-0.4157318745	C	2.1970045368	3.215445234	0.3670050026
H	-3.2926004889	3.2967405668	-0.2943184442	H	2.8149216876	3.2528163356	-0.5247127642
C	-1.3977663118	3.2944327789	0.6828652003	C	0.831835988	3.1904800085	0.2553547122
H	-1.8097209473	3.3836159779	1.6811530245	H	0.3509341733	3.2120147199	-0.7154836922
C	0.010851012	3.2488295953	0.5150299598	C	0.029971645	3.1583142705	1.4260087698
C	0.570497495	3.1806335779	-0.7855119791	C	0.6386649792	3.1802287239	2.7058879458
C	1.9794033911	3.2005730145	-0.9540103521	C	-0.1661304771	3.2150288888	3.874114828
H	2.3957887364	3.1631667866	-1.9532155352	H	0.3097990425	3.2473151654	4.8465720593
C	2.7937285525	3.2732724288	0.1446126721	C	-1.5307664246	3.2137051923	3.7590550836
H	3.871675094	3.301076767	0.0201768524	H	-2.1504905117	3.2519327856	4.6494131944
C	2.2618273755	3.2920961972	1.4542207909	C	-2.1620503831	3.1405542904	2.4963689564
H	2.94279982	3.3260590677	2.2955405134	H	-3.2438094431	3.1167693235	2.4521283011
C	0.8982210882	3.2492630686	1.6367240175	C	-1.3964261701	3.0823037681	1.3540198091
C	1.0726267592	3.1603655923	4.016215026	C	-3.2900276166	2.8207850528	-0.0758625083
H	1.9129365697	2.463188155	3.9191800901	H	-3.7315753908	2.1315390177	0.6532483077
H	1.4661681546	4.167869861	4.1943357567	H	-3.7395658601	3.8118193295	0.0556378334
C	0.2145095821	2.711421935	5.1737577698	C	-3.5670721738	2.2899885841	-1.4613151153
H	0.7147327077	2.9944904242	6.1107736314	H	-4.6143600035	2.5056577786	-1.7162606905
H	-0.7646134893	3.2090350634	5.1429533115	H	-2.9240355225	2.7884353262	-2.1998118825
C	-0.5735649656	0.7594898372	6.2435608936	C	-3.7436939541	0.2688371111	-2.6678426859
H	-1.6539296733	0.9618102113	6.2090420717	H	-3.0170221836	0.4780135988	-3.4662297222
H	-0.1706092643	1.2118873846	7.1608431334	H	-4.722707576	0.6531197519	-2.9878975211
C	-0.3166432823	-0.7263072257	6.2980276894	C	-3.8703738127	-1.2183558342	-2.4467625634
H	0.765319425	-0.9054996769	6.21612291	H	-4.5102000542	-1.3967240669	-1.5702713146
H	-0.6524987131	-1.106893042	7.2736658639	H	-4.361906139	-1.6642763373	-3.3238086931
C	-0.8004328805	-2.7725389627	5.2313335146	C	-2.6382523132	-3.1834042778	-2.0255043138
H	-1.7316149392	-3.2450465634	4.903991243	H	-1.7502092114	-3.6269102187	-2.4862203212
H	-0.5663981406	-3.1450320574	6.2366000932	H	-3.5232448609	-3.6266993961	-2.499336067
C	0.3147024202	-3.158717548	4.2783443835	C	-2.6438993496	-3.5080902268	-0.5437099905
H	0.6783714044	-4.1715086821	4.491306231	H	-2.986322151	-4.5356405645	-0.369715538
H	1.1508479493	-2.455683912	4.3819128921	H	-3.3153059176	-2.8197079355	-0.0149470571
C	0.6204756764	-3.2710973374	1.9085866851	C	-1.0709897148	-3.4533651962	1.2574095033
C	1.9863590598	-3.4055182749	2.0071829309	C	-2.0421370157	-3.6022420884	2.2208890785
H	2.4800652462	-3.4423258056	2.9705594051	H	-3.0867059731	-3.7094919317	1.9553303853
C	2.7677828308	-3.4899158417	0.8317309783	C	-1.6770983929	-3.6084697635	3.5869340103

H	3.8434778725	-3.6016369592	0.9284893943
C	2.1968686068	-3.4109202038	-0.4108772334
H	2.8048715712	-3.4468407452	-1.3068067212
C	0.7892053846	-3.281744675	-0.5318291377
C	-0.0230985863	-3.2600171803	0.630523232
C	-1.4357602434	-3.2156741228	0.5076960701
H	-2.0478473075	-3.2404877851	1.4000488785
C	-2.009323153	-3.1681525054	-0.7356608793
H	-3.0902706047	-3.1490843486	-0.8342519597
C	-1.2200916878	-3.1294275406	-1.907514432
H	-1.7143400063	-3.0711949503	-2.8693950779
C	0.1533836919	-3.1642724367	-1.8096813013
C	0.4731948704	-3.1929097465	-4.1731066154
H	-0.3077707472	-2.4405368585	-4.336932392
H	0.0305279306	-4.188526682	-4.300973184
C	1.6067358514	-2.9719802351	-5.1602987856
H	2.5267788084	-3.4052616407	-4.7577121275
H	1.374912373	-3.4811929039	-6.1047066494
C	1.0603145766	-1.0590476216	-6.4273409875
H	-0.000077231	-1.3005423388	-6.2707825625
H	1.3651414627	-1.4784546294	-7.3971729999
C	1.2097822695	0.4428385875	-6.4693814226
H	2.2710389037	0.7240924192	-6.5526739503
H	0.6961632038	0.8119994236	-7.3686216742
C	0.3748941427	2.3879466237	-5.4218870447
H	-0.161057197	2.5978110418	-6.358562577
H	1.3118069047	2.9626869905	-5.4279879611
C	-0.501963644	2.8193034325	-4.2700715402
H	-0.9529065218	3.7968148588	-4.4786135203
H	-1.2998099372	2.0785647685	-4.1425962111
C	5.2898750305	-0.2335882921	-3.9304762946
H	5.1838677778	0.6286624223	-4.5918811209
H	6.2386597145	-0.140066806	-3.3997158384
C	-2.1622809434	0.3544255354	2.6604048633
H	-1.7093035252	0.6627199549	3.6030110221
H	-2.7664937747	1.1803265179	2.2767899057
C	-2.9810199026	-0.9172696436	2.8307168886
H	-3.4660311159	-1.1812477611	1.8820578669
H	-2.2985049385	-1.730759225	3.0901561827
C	-4.0331562763	-0.7535998921	3.9257359142
H	-4.6934426682	0.0888094865	3.6833260326
H	-3.5237363897	-0.5067574525	4.8641691493
C	-4.8621574005	-2.0226861004	4.1109093893
H	-5.6068375235	-1.8952509209	4.9027154752
H	-5.3916252929	-2.2865285002	3.1884237164
H	-4.2231021408	-2.8703167977	4.3838616254
C	5.1953526263	-1.546733956	-4.7012215893
H	4.2121270588	-1.6025979053	-5.1832747494
H	5.2634876955	-2.3828977979	-3.9937203507
C	6.2977011117	-1.6630799015	-5.7536875238
H	7.2794507126	-1.6107506744	-5.2667136861
H	6.2391655834	-0.8068876592	-6.4373452448
C	6.1833298628	-2.9627196245	-6.54819139
H	5.2180781251	-3.0216700304	-7.0636876889
H	6.2652748018	-3.8338656642	-5.8886595437
H	6.9737755252	-3.0342014036	-7.3015211182
H	-2.4555953574	-3.7337712421	4.333377668
C	-0.3751758843	-3.4387019841	3.9776885328
H	-0.106556739	-3.4164775649	5.0269781632
C	0.6401983257	-3.2917987258	2.9978385517
C	0.3091238785	-3.347629641	1.6201299061
C	1.3343457194	-3.2859468222	0.6414723862
H	1.0745498992	-3.3703159739	-0.4058674883
C	2.6401518746	-3.1457456794	1.0318788346
H	3.4288770067	-3.1125416776	0.2866603828
C	2.9900449559	-3.0283095667	2.3963102297
H	4.0322355278	-2.897373008	2.6604238709
C	2.0087848736	-3.0787940108	3.3616391285
C	3.5612916126	-2.9427991792	5.1678835669
H	4.1615156223	-2.1789413781	4.6591367498
H	4.0048989914	-3.9257869636	4.9669812255
C	3.5317193088	-2.654090813	6.6593032218
H	2.642626316	-3.1147054882	7.0993644992
H	4.4162366378	-3.0959001786	7.1361469476
C	4.7383942883	-0.6568543483	7.0032463238
H	5.3394137928	-0.9076789033	6.1182069067
H	5.2806236245	-1.0076880697	7.8934112339
C	4.5908243427	0.844232875	7.0733241093
H	3.9337976002	1.1303918335	7.9092201475
H	5.5831728064	1.2759113633	7.267350021
C	4.2769867864	2.7062412961	5.6531002872
H	5.3244359121	2.9710014145	5.8567123128
H	3.6383375114	3.2931356693	6.3283517
C	3.9757498941	3.0468730999	4.2126457331
H	4.3881079267	4.030525059	3.9583704278
H	4.4380962084	2.2887338478	3.5698542702
C	0.0342811201	-0.0293677315	8.5067014018
H	0.5403289939	0.8839827613	8.8267778391
H	-0.9948054773	0.008057463	8.8676828395
C	0.0035394727	0.1305999158	-1.4584005662
H	-1.018229877	0.3676844669	-1.7559701422
H	0.6420499935	0.9868876431	-1.6892982284
C	0.7652171943	-1.2754083296	8.9967742935
H	0.2451663978	-2.1662740941	8.6221645743
H	1.7758296912	-1.2811023711	8.5715938033
C	0.8429008952	-1.319423809	10.5226140716
H	-0.1700961592	-1.3145647278	10.9438972002
H	1.3400101327	-0.411723008	10.8872226945
C	1.6009041687	-2.5518024449	11.012603222
H	1.6555807997	-2.5716154726	12.1053478946
H	1.1072406652	-3.4725186529	10.6821586066
H	2.624797999	-2.5613302173	10.6221220904
C	0.4956857593	-1.1413171843	-2.133378023
H	1.5382708222	-1.3361408445	-1.8508612551
H	-0.1072750059	-1.9772157641	-1.769265254
C	0.3787455512	-1.0398142062	-3.6528415173
H	0.9537591941	-0.1769479462	-4.0125023912
H	-0.6710505278	-0.8595878331	-3.910764703
C	0.8659686699	-2.3105495736	-4.3455783692
H	0.7702904061	-2.2287845673	-5.4327458644
H	1.9184590653	-2.5082931645	-4.112897721
H	0.2826635191	-3.1799959734	-4.0211645948

Deprotection attempt on ***p*-H[4]R**

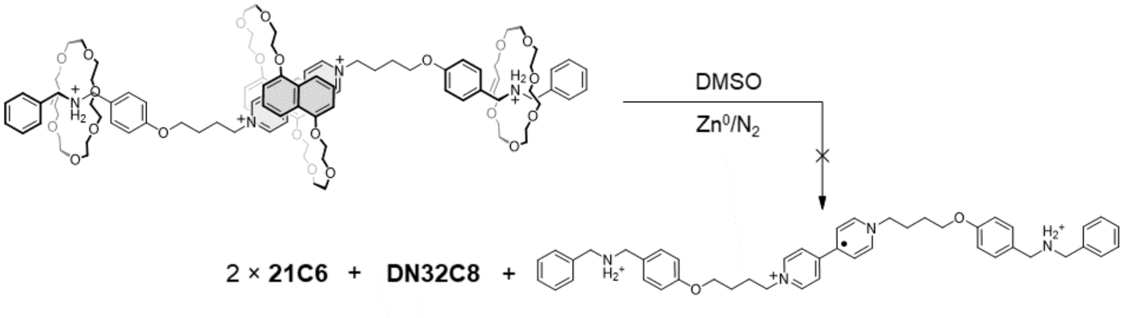

Scheme S5. Deprotection attempted on rotaxane ***p*-H[4]R** via polarity effect.

A sample of ***p*-H[4]R** in DMSO (1 mL, 2 × 10^{-3} M) was loaded with zinc dust (50 mg) and stored over 6 months at room temperature in a N_2 glove box. Figure S19 shows the UV-vis spectra for the initial and final stages, where no significant differences are observed. This confirms that the hetero[4]rotaxane remains assembled, with the substrate permanently protected.

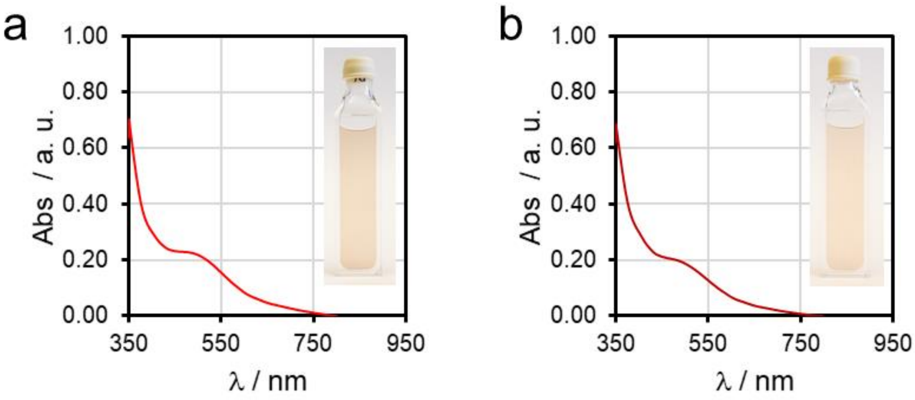

Figure S19. UV-vis spectra of ***p*-H[4]R** in DMSO, a) before mixing with Zn^0, and b) after storing for 6 months in the presence of Zn^0 under a N_2 atmosphere. The photographs show the collected samples.

Cyclic voltammetry analyses

Voltammograms of [**1**·H_2][PF_6]$_4$, [**1**·H_2⊂(**21C6**)$_2$] [PF_6]$_4$, ***p*-H[4]R**, and ***t*-H[4]R** were recorded in MeCN, employing *n*-Bu_4NPF_6 as the electrolyte (100 mM) at 25 ºC, and at a sweep rate of 100 mV/s. The working electrode was a glassy carbon disc (~0.5 mm in diameter), the counter electrode consisted of a platinum mesh, and the pseudo-reference electrode was a silver wire. The ferrocene (Fc) / ferrocenium (Fc^+) redox couple was used to reference all measurements.

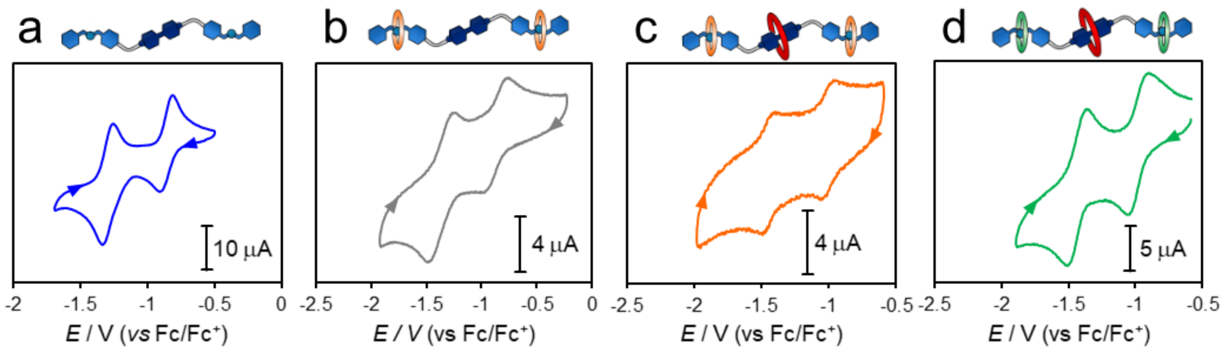

Figure S20. Cyclic voltammograms of the unprotected species a) [**1**·H_2]$^{4+}$ (1.0 mM) and b) [**1**·H_2⊂(**21C6**)$_2$]$^{4+}$ (1.0 mM), and the protected systems c) ***p*-H[4]R** (0.1 mM) and d) ***t*-H[4]R** (1.0 mM). Arrows indicate the sweep direction.

As shown in Figure S20, all cyclic voltammograms showed a reversible two-electron process for the reduction of the viologen substrate. As expected, the half-wave potentials ($E^1_{1/2}$ and $E^2_{1/2}$, summarized in Table S5) of the unprotected species, thread [**1**·H_2]$^{4+}$ and [3]rotaxane [**1**·H_2⊂(**21C6**)$_2$]$^{4+}$, are equivalent; *i.e.* $E^1_{1/2}$ is -0.86 V in both cases (as for the formation of the corresponding radical trications). In contrast, the presence of the protecting unit (**DN32C8**) in ***p*-H[4]R** and ***t*-H[4]R** produces a cathodic shift of ~140 mV for the same redox pair. This can be ascribed to the stabilization effect of **DN32C8** to the viologen as result of a charge transfer process within the rotaxane structures, *i.e.* from the electron-rich ring to the electron-poor substrate. This stabilization effect was calculated to be ~13 kJ mol^{-1} for ***p*-H[4]R** and ***t*-H[4]R** with respect to [**1**·H_2]$^{4+}$ and [**1**·H_2⊂(**21C6**)$_2$]$^{4+}$.

Table S5. Half-wave potentials ($E_{1/2}$) of the redox pairs for the thread molecule and the assembled MIMs (*vs* Fc/Fc^+).

System	$E^1_{1/2}$ (1st reduction) / V	$E^2_{1/2}$ (2nd reduction) / V
$[\mathbf{1}\cdot H_2]^{4+}$	-0.86	-1.30
$[\mathbf{1}\cdot H_2\subset(\mathbf{21C6})_2]^{4+}$	-0.86	-1.36
***p*-H[4]R**	-1.00	-1.46
***t*-H[4]R**	-0.99	-1.43

Rotaxane (*p*-H[4]R) degradation *via* ring-opening metathesis

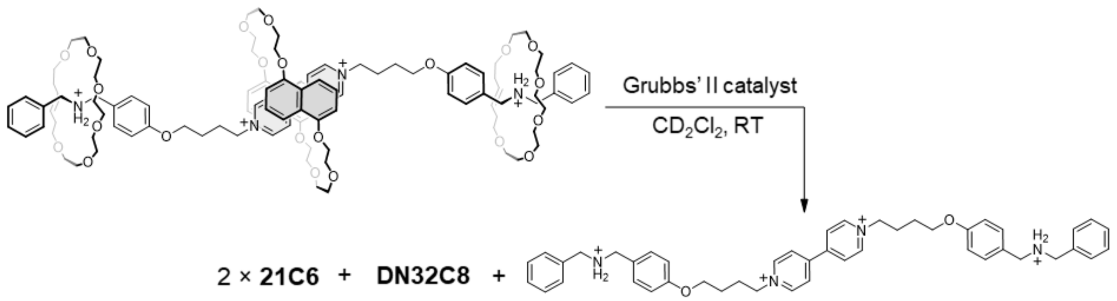

Scheme S6. Degradation of rotaxane ***p*-H[4]R** *via* ring-opening metathesis.

Disassembly of the permanently protected rotaxane, ***p*-H[4]R**, was assessed by ring-opening metathesis (Scheme S6). A solution of ***p*-H[4]R** (2 mM) in CD_2Cl_2 was mixed with Grubbs' II catalyst under N_2 atmosphere and kept at RT for 24 h. The sample was analysed by ^{1}H NMR spectroscopy within this period. The results (Figure S21) suggest full disassembly of the MIM, releasing the protecting unit and the viologen-based thread component into solution.

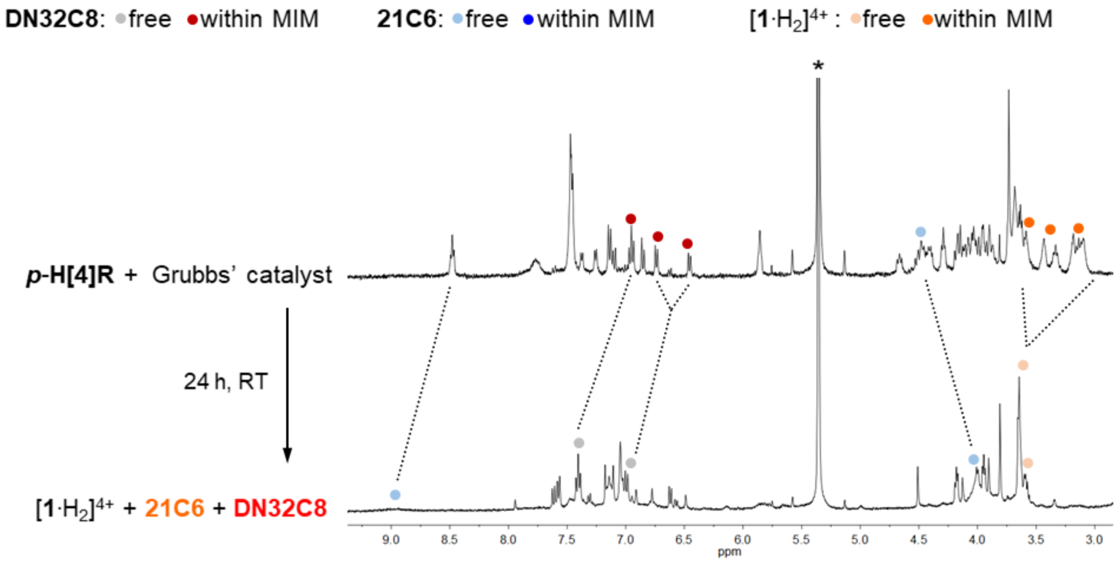

Figure S21. Partial ^{1}H NMR spectra (400 MHz, CD_2Cl_2) for the degradation of ***p*-H[4]R** through ring-opening metathesis. Diagnostic signals have been labeled. Broadening of the viologen resonances at $\delta \approx 9$ ppm, suggest that the released axle and **DN32C8** might undergo pseudorotaxane formation in solution after ring-opening.

Unstoppering process on *t*-H[4]R

Solutions of rotaxane ***t*-H[4]R** at 1 × 10^{-3} M concentration were analyzed towards three different stimuli: base, polarity, and heat.

Base trigger

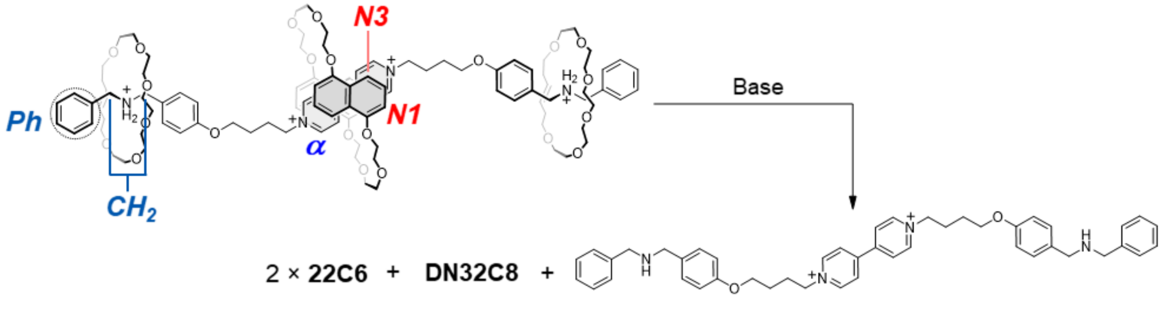

Scheme S7. Unstoppering process on ***t*-H[4]R** triggered by base addition.

A base solution (1 M, $NaOH_{(aq)}$, 1 equiv) was added to a CD_3CN solution of ***t*-H[4]R** and stirred for 10 min at room temperature, then a ^1H NMR spectrum was collected (Figure S22).

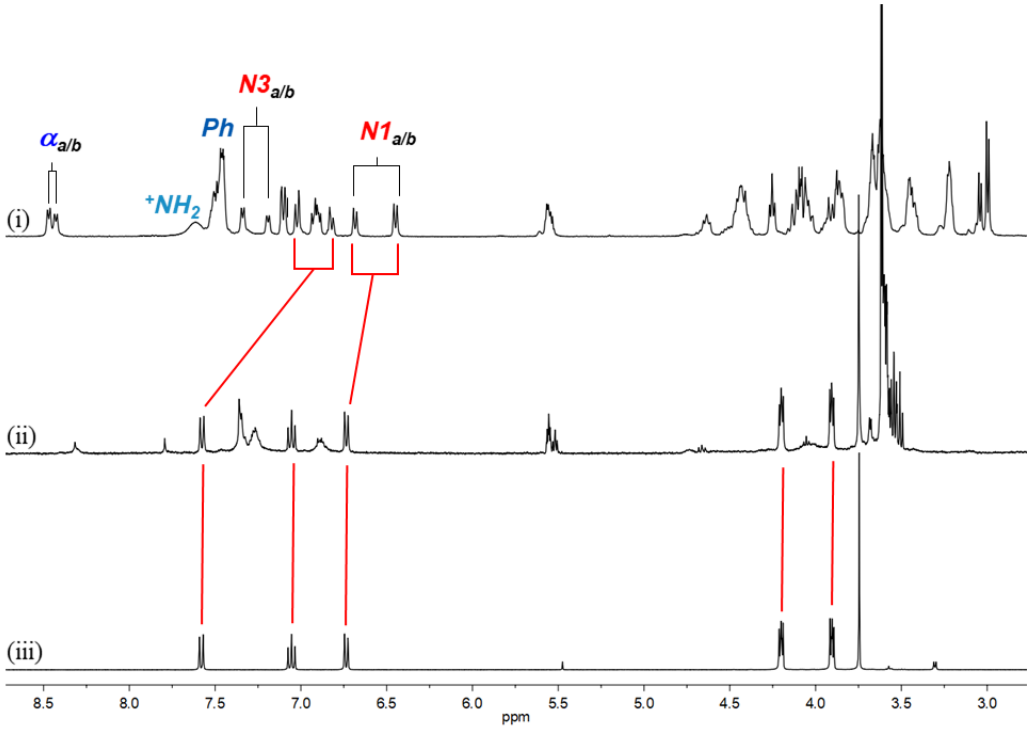

Figure S22. Partial ^1H NMR spectra (400 MHz, CD_3CN) of ***t*-H[4]R** (i) before and (ii) after the addition of one equiv of base ($NaOH_{(aq)}$), and (iii) pure **DN32C8**.

Based on the chemical shifts and the number of observable resonances, it is clear that the three free macrocycles were deposited in solution after deprotonation of the ammonium stations. The parent ions [**DN32C8** + Na]$^+$ and [**22C6** + Na]$^+$ were detected by HRMS at *m/z* 570.7601 and 340.9149, respectively, while the ion [**1** + **DN32C8** + (2 × **21C6**) + (2 × PF_6) + 2H]$^{2+}$ at *m/z* 1085.0029, observed in Figure S66, was no longer identified in the spectrum (Figure S23a). In addition, just after mixing with base, the solution turned from red to pale yellow and the charge transfer band (490 nm) in the UV-vis spectrum was no longer detected (Figure S23b), suggesting that ***t*-H[4]R** was successfully disassembled.

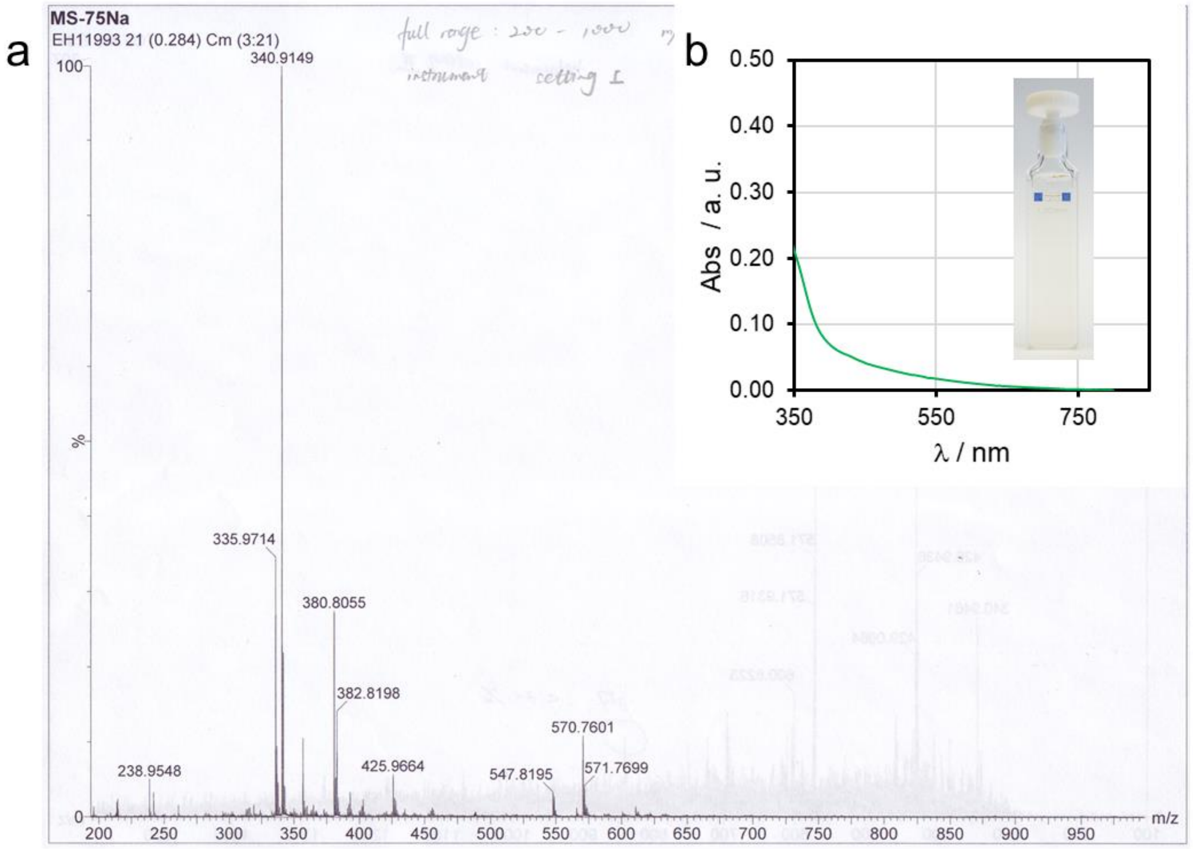

Figure S23. a) HRMS of ***t*-H[4]R** after the addition of one equiv of base ($NaOH_{(aq)}$). b) UV-vis spectrum (MeCN) of the disassembled system upon addition of base. The photograph shows the analyzed sample.

Polarity and temperature stimuli

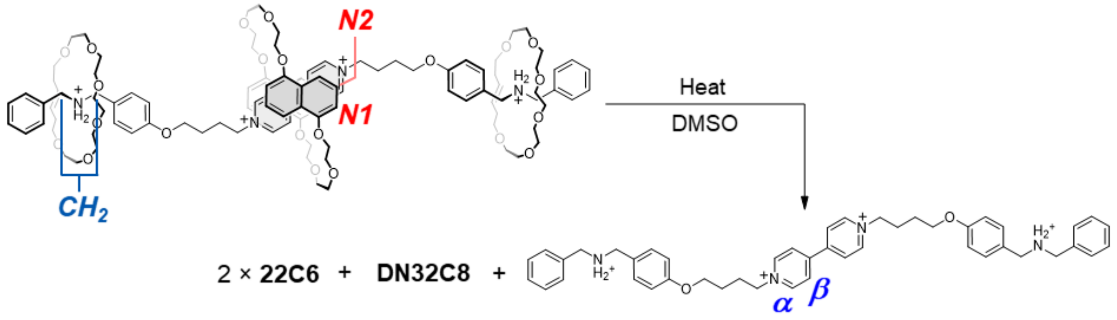

Scheme S8. [4]Rotaxane disassembly triggered by heat and polarity.

In contrast to other non-competitive solvents, such as CH_2Cl_2 or MeCN, DMSO produced unstoppering at room temperature, releasing the protecting macrocycle **DN32C8**. Heating the system causes fast unstoppering that renders all free components in solution, as clearly detected by ^{1}H NMR spectroscopy (Figure S24).

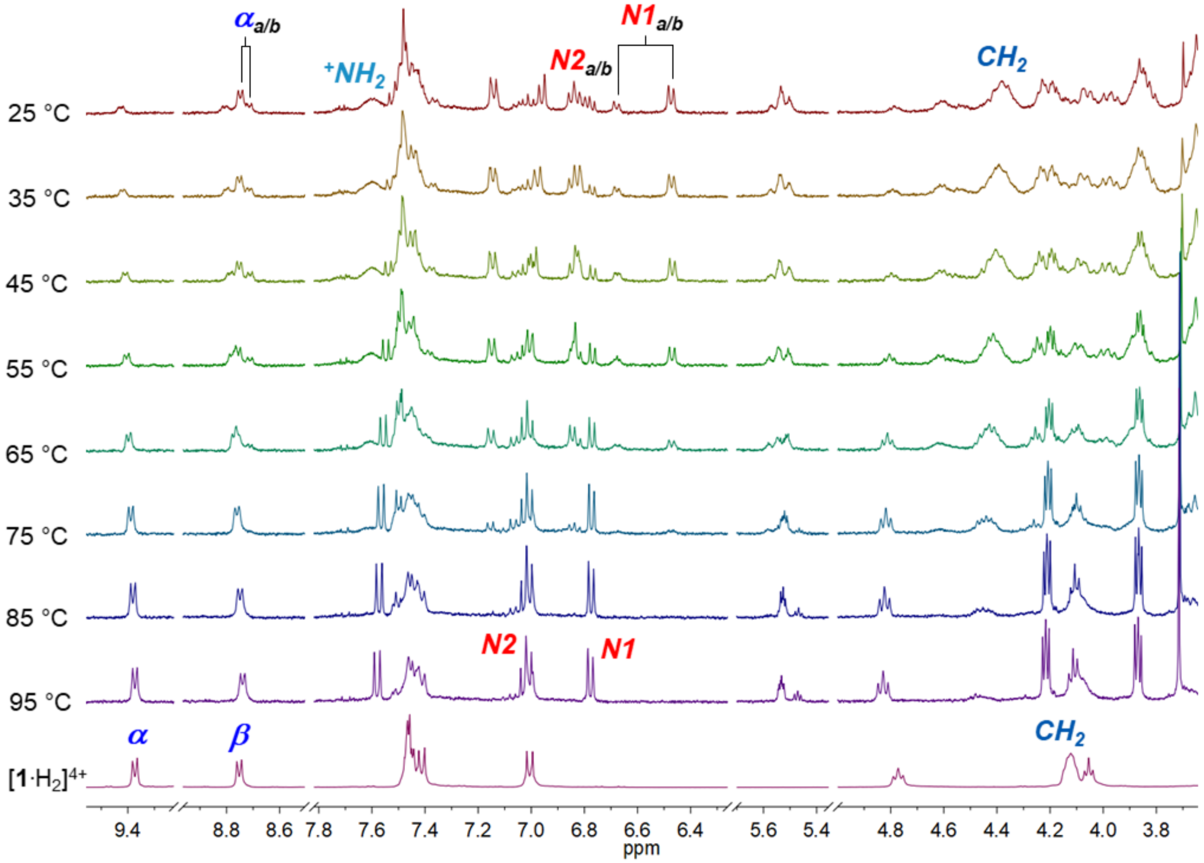

Figure S24. Partial ^{1}H NMR spectra (400 MHz, CD_3CN) tracking the unstoppering of ***t*-H[4]R** directed by two combined stimuli (polarity and heat).

In addition, HRMS proved the presence of [**DN32C8** + H]$^+$ at *m/z* 548.2, [**1** + PF_6]$^+$ at *m/z* 837.9, and [**22C6** + Na]$^+$ at *m/z* 341.2, generated after the unstoppering process, whereas the ion [**1** + **DN32C8** + (2 × **22C6**) + (2 × PF_6) + 2H]$^{2+}$, observed at *m/z* 1085.0029 in Figure S66, was no longer detected (Figure S25).

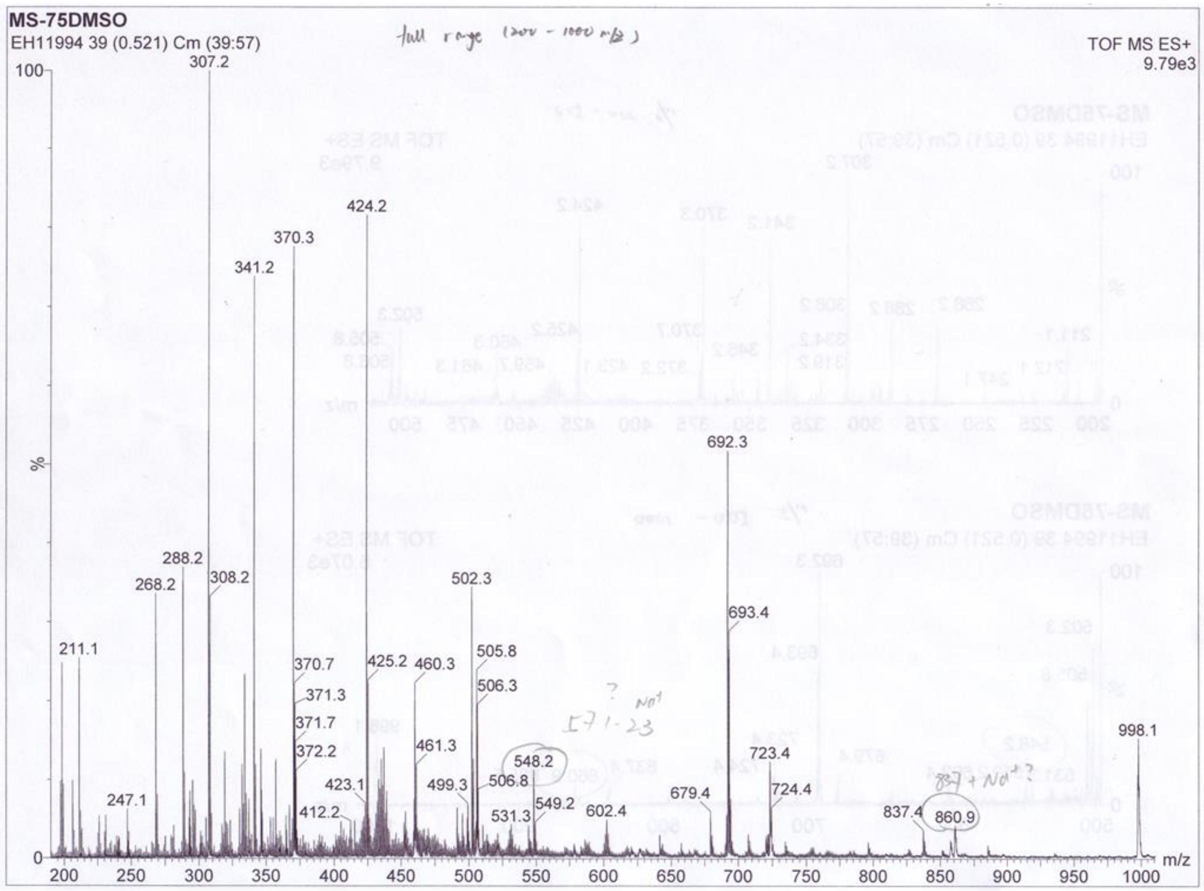

Figure S25. HRMS for rotaxane ***t*-H[4]R** after heating in DMSO at 90 ºC.

Controlled deprotection of *t*-H[4]R

Base trigger

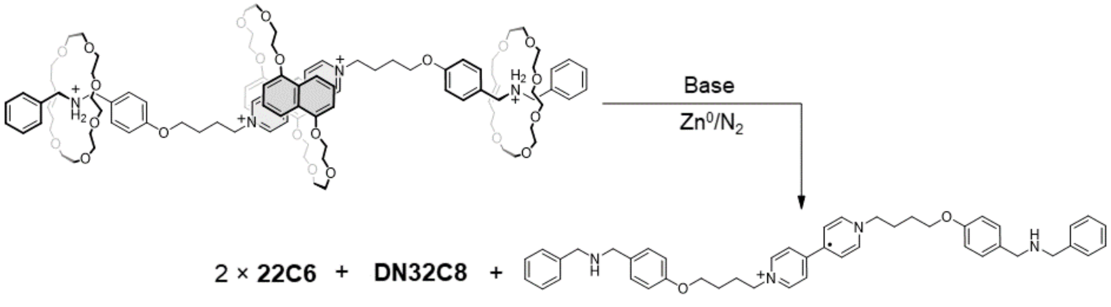

Scheme S9. Controlled deprotection through base trigger.

A suspension containing ***t*-H[4]R** (1 mL, 2 × 10^{-3} M in CH_3CN) and zinc dust (50 mg) was treated with one equiv of base (1 M $NaOH_{(aq)}$) under a N_2 atmosphere; an immediate change of color from red to blue due to the formation of [**1**·H_2]$^{(3+)•}$ was noticed; this is a consequence of the unstoppering/deprotection sequence. Figure S26 shows the UV-vis spectrum of the system after deprotection, this experiment matches with the one reported for species [**1**·H_2]$^{(3+)•}$, shown in Figure S2a.

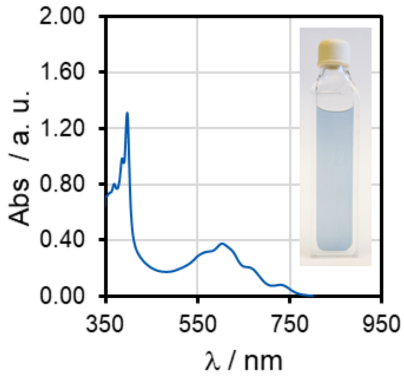

Figure S26. UV-vis spectrum (MeCN) of ***t*-H[4]R** after the unstoppering/deprotection process. The photograph shows the prepared sample.

Polarity and temperature effect

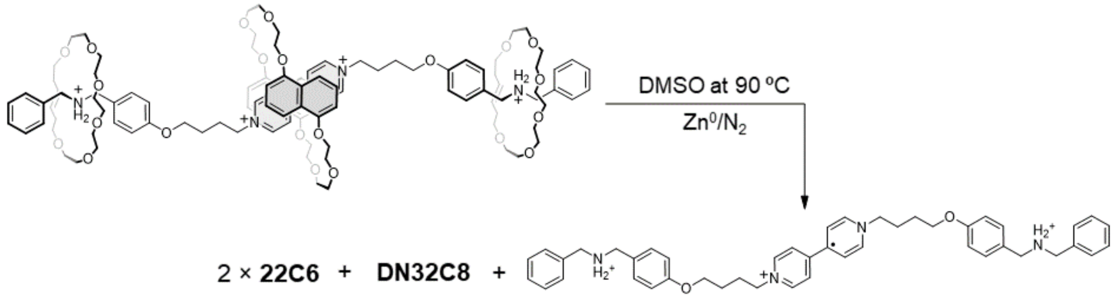

Scheme S10. Controlled deprotection via polarity and thermal stimuli.

A solution of ***t*-H[4]R** (400 μL, 1 x10^{-3} M) was prepared in deaerated DMSO, then loaded with zinc dust (10 mg) and heated at 90 ºC. UV-vis spectra were collected every minute followed by equilibration to RT. Figure S27 shows the collected spectra along with photographs of the samples. The registered bands in the spectra corroborate the gradual formation of [**1**·H_2]$^{(3+)\bullet}$, which is produced by the thermal-triggered deprotection process.

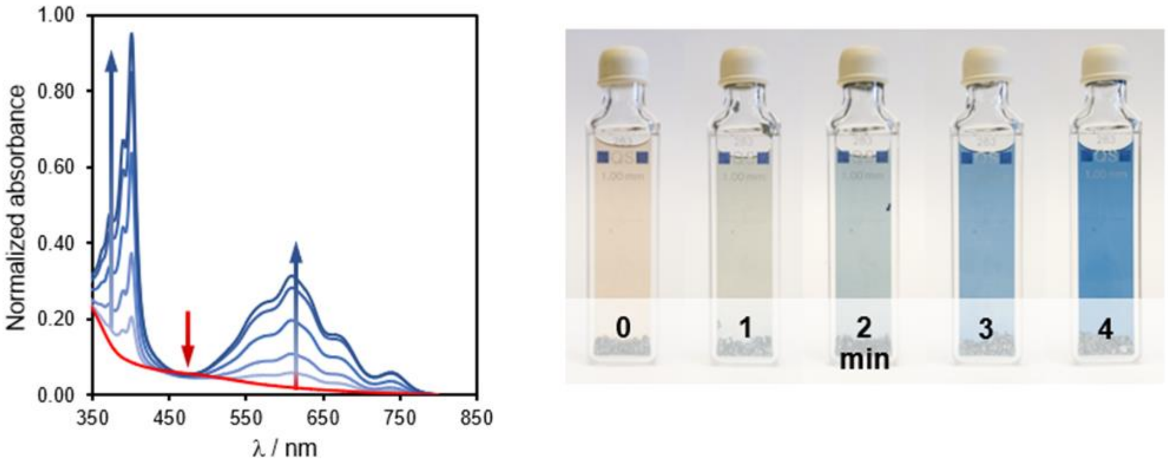

Figure S27. UV-VIS spectra for the gradual deprotection of ***t*-H[4]R** via thermal/polarity effect. Photographs show the gradual deprotection observed at macroscopic level.

Synthesis

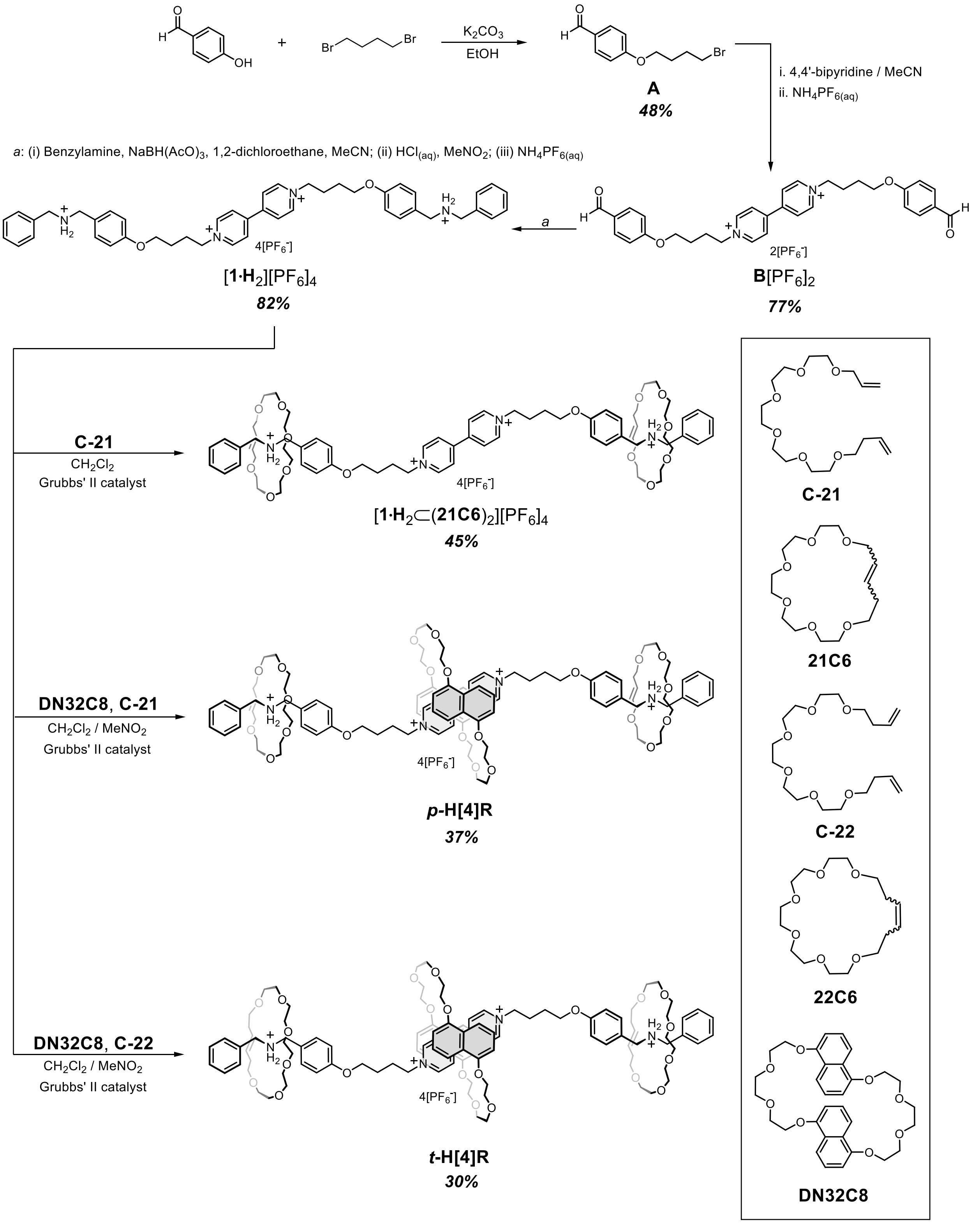

Scheme S11. Synthesis of the targeted hetero[4]rotaxanes and their precursors.

Functionalized benzaldehyde A

The synthesis was adapted from a reported method.[10] 1,4-Dibromobutane (14.6 g, 66.7 mmol) and 4-hydroxybenzaldehyde (2.0 g, 16.7 mmol) were dissolved in EtOH (60 mL), and K_2CO_3 (4.6 g, 33.3 mmol) was then added in one portion; the resulting suspension was heated under reflux and stirred for 10 h. After cooling to room temperature, the mixture was filtered, and the filtrate evaporated under vacuum. Column chromatography (SiO_2, petroleum ether:DCM, 1:1, R_f = 0.49) afforded compound **A** as a yellow oil (2.1 g, 8.2 mmol, 48% yield); this compound crystalizes over time. ^{1}H NMR (400 MHz, DMSO-d_6) δ_H 9.86 (s, 1H), 7.86 (d, *J* = 8.7 Hz, 2H), 7.12 (d, *J* = 8.7 Hz, 2H), 4.13 (t, *J* = 6.2 Hz, 2H), 3.61 (t, *J* = 6.6 Hz, 2H), 2.11–1.74 (m, 4H); ^{13}C NMR (100 MHz, DMSO-d_6) δ_C 191.8, 164.0, 132.3, 130.1, 115.4, 67.7, 35.2, 29.4, 27.7; ESI-HRMS, *m/z* for [**A** + H]$^+$ 257.0176 (found), 257.0177 (calculated), relative error -0.4 ppm.

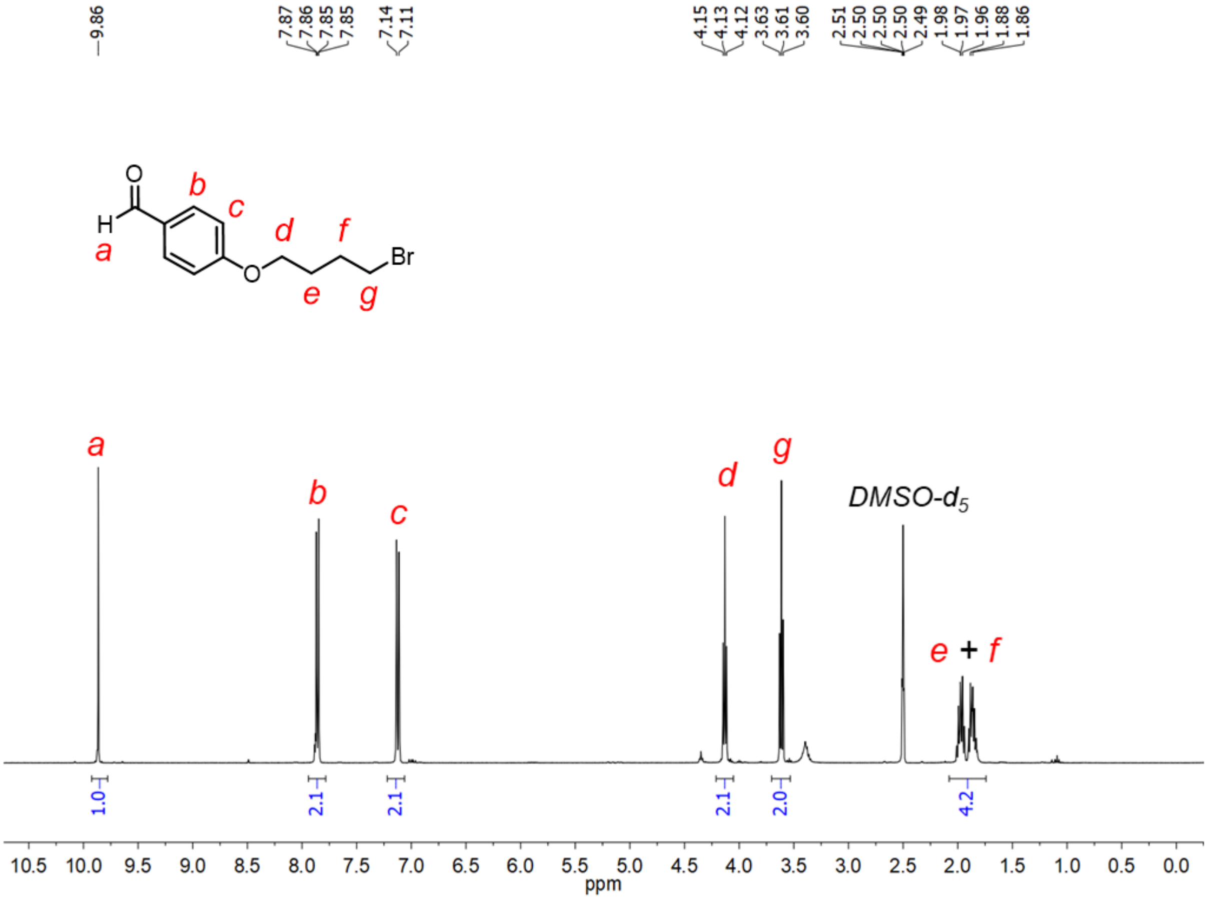

Figure S28. ^{1}H NMR spectrum (400 MHz, DMSO-d_6) of compound **A**.

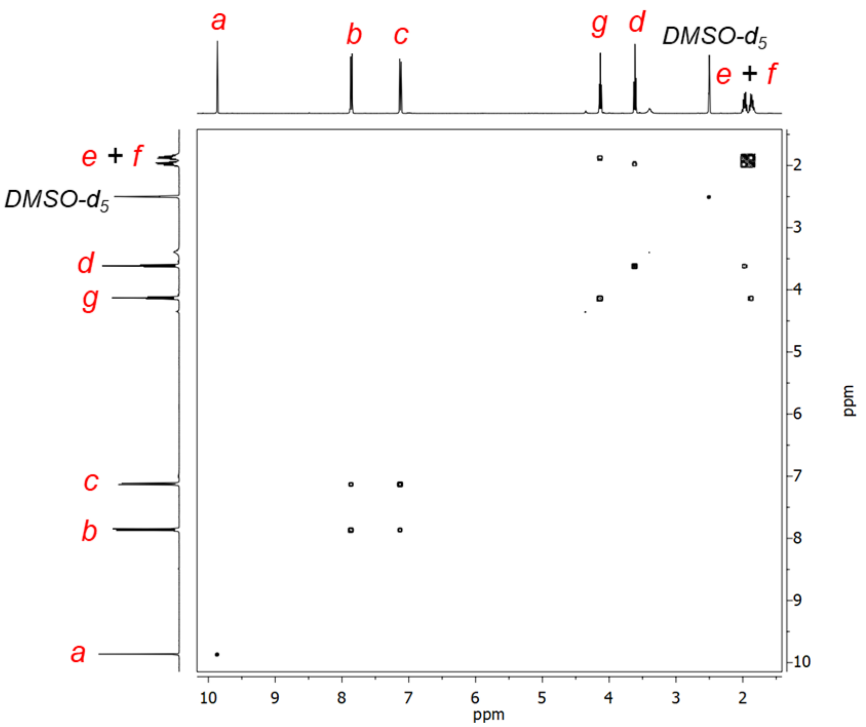

Figure S29. ^{1}H-^{1}H COSY NMR spectrum (400 MHz, DMSO-d_6) of compound **A**.

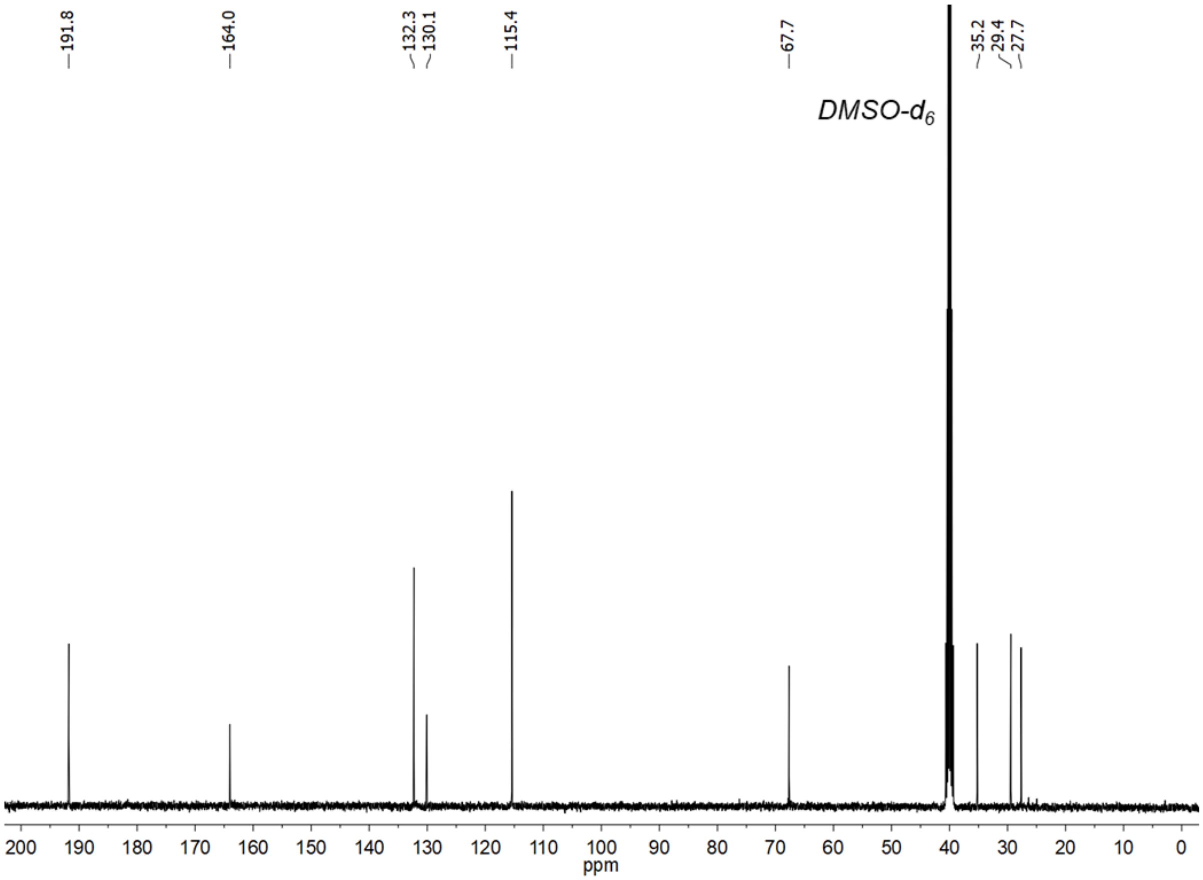

Figure S30. ^{13}C {^{1}H} NMR spectrum (100 MHz, DMSO-d_6) of precursor **A**.

4,4’-Bipyridinium salt B[PF_6]$_2$

A solution of precursor **A** (866.0 mg, 3.38 mmol) and 4,4’-bipyridine (134.5 mg, 0.84 mmol) in MeCN (3 mL) was heated under reflux for 4 d and then cooled to room temperature. Separating the precipitate by filtration allowed isolation of **B**Br_2 as a yellow solid, which was washed with MeCN (30 mL) and $CHCl_3$ (30 mL) and dissolved in H_2O (20 mL). The addition of NH_4PF_6 (522.0 mg, 3.20 mmol) in one portion caused precipitation of a white solid, which was isolated on a Buchner funnel, washed with H_2O (50 mL) and cold MeOH (30 mL), and air-dried to isolate **B**[PF_6]$_2$. ^{1}H NMR (400 MHz, DMSO-d_6) δ_H 9.86 (s, 2H), 9.46 (d, J = 5.8 Hz, 4H), 8.82 (d, J = 6.0 Hz, 4H), 7.87 (d, J = 8.7 Hz, 4H), 7.12 (d, J = 8.7 Hz, 4H), 4.80 (t, J = 7.1 Hz, 4H), 4.17 (t, J = 6.2 Hz, 4H), 2.18 (m, 4H), 1.83 (m, 4H); ^{13}C NMR (100 MHz, DMSO-d_6) δ_C 191.8, 163.9, 149.0, 146.3, 132.3, 130.1, 127.1, 115.4, 67.8, 61.0, 28.1, 25.6. ESI-HRMS, m/z for [**B**]$^{2+}$ 255.1254 (found), 255.1259 (calculated), relative error -2.0 ppm.

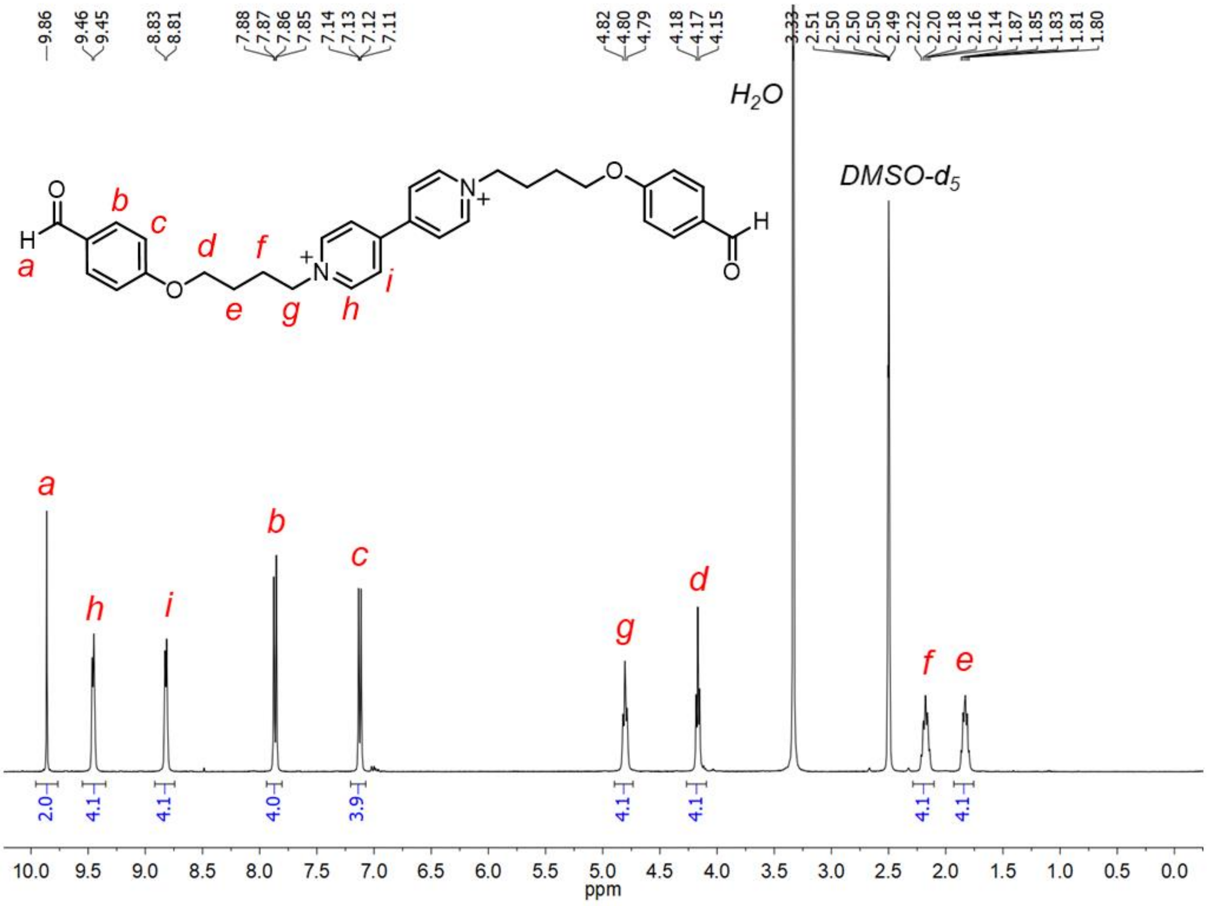

Figure S31. ^{1}H NMR spectrum (400 MHz, DMSO-d_6) of salt **B**[PF_6]$_2$.

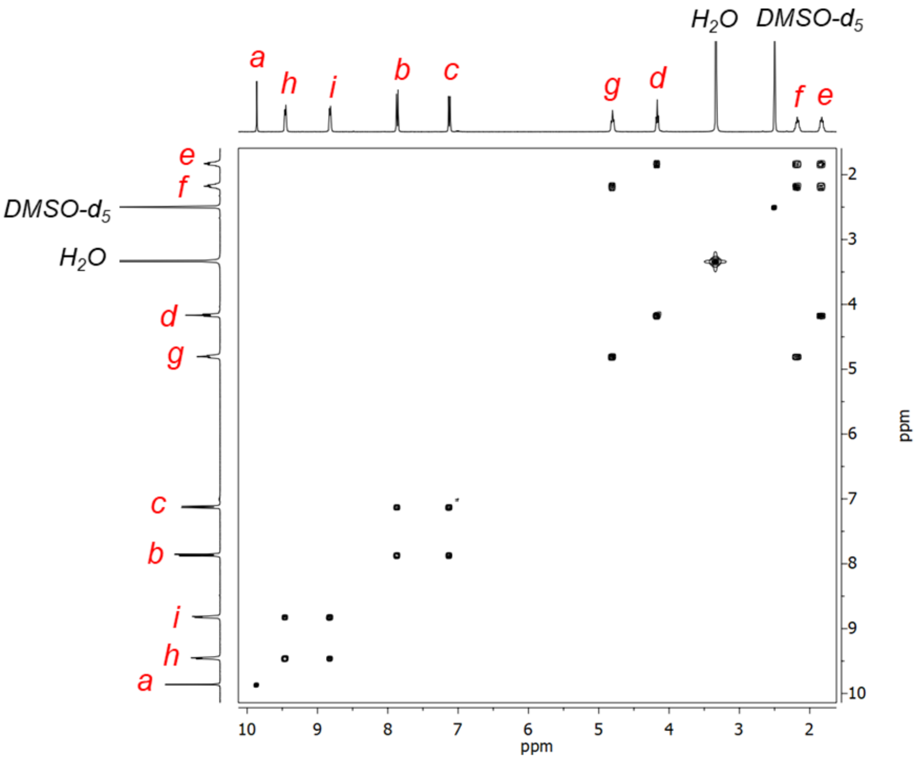

Figure S32. ^{1}H-^{1}H COSY NMR spectrum (400 MHz, DMSO-*d*$_6$) of compound **B**[PF$_6$]$_2$.

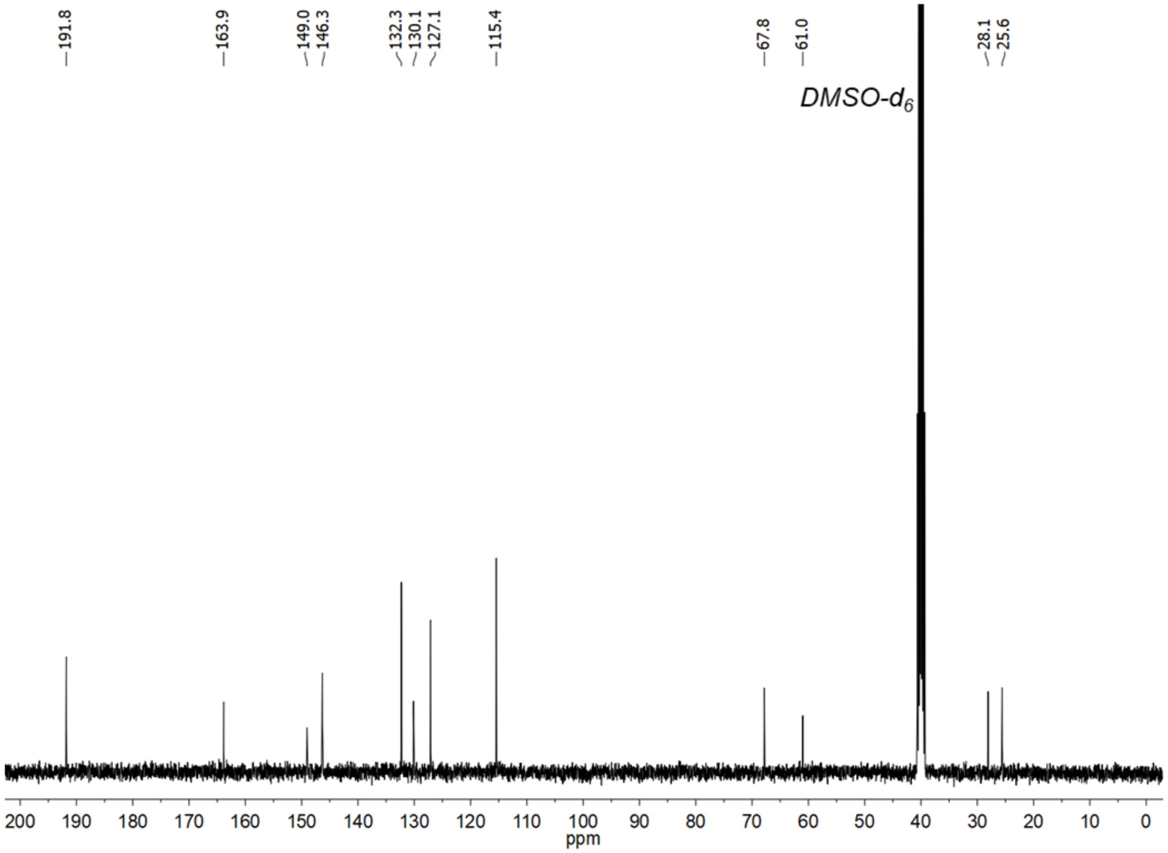

Figure S33. ^{13}C {^{1}H} NMR spectrum (100 MHz, DMSO-*d*$_6$) of precursor **B**[PF$_6$]$_2$.

Thread [$\mathbf{1}\cdot H_2$][PF_6]$_4$

Precursor **B**[PF_6]$_2$ (1.0 g, 1.25 mmol) and benzylamine (297.5 mg, 2.75 mmol) were dissolved in MeCN (12.5 mL) and 1,2-dichloroethane (5 mL), followed by the addition of $NaBH(OAc)_3$ (831.3 mg, 3.90 mmol). The resulting suspension was stirred for 16 h at room temperature and an extra portion of $NaBH(OAc)_3$ was added (415.7 mg, 1.95 mmol). After stirring the mixture for 16 h more, it was quenched with saturated $NaHCO_{3(aq)}$ (50 mL) and the organic layer was separated. The aqueous layer was extracted with $MeNO_2$ (3 × 15 mL) and all combined organic phases were dried (Na_2SO_4) and evaporated under reduced pressure. The brown residue was then dissolved in $MeNO_2$ (5 mL) and 12 M aqueous HCl (990 μL) was added dropwise as the residue was rapidly stirred. After 10 min of continuous stirring, a solution of NH_4PF_6 (1.0 g, 6.3 mmol) in H_2O (15 mL) was added. Finally, the organic phase was separated, washed with H_2O (3 × 25 mL) and dried (Na_2SO_4). Rotary evaporation yielded salt [$\mathbf{1}\cdot H_2$][PF_6]$_4$ as a brown crystalline solid (1.3 g, 1.02 mmol, 82% yield). ^{1}H NMR (400 MHz, DMSO-*d*$_6$) δ_H 9.37 (d, *J* = 6.9 Hz, 4H), 9.11 (br, 4H), 8.75 (d, *J* = 7.0 Hz, 4H), 7.68–7.30 (m, 10H), 7.41 (d, *J* = 8.7 Hz, 4H), 7.01 (d, *J* = 8.7 Hz, 4H), 4.77 (t, *J* = 7.4 Hz, 4H), 4.12 (br, 8H), 4.05 (t, *J* = 6.2 Hz, 4H), 2.17 (m, 4H), 1.82 (m, 4H); ^{13}C NMR (100 MHz, DMSO-*d*$_6$) δ_C 159.5, 149.3, 146.2, 132.2, 132.1, 130.4, 129.6, 129.2, 127.1, 124.0, 115.0, 67.4, 61.2, 50.3, 50.1, 28.2, 25.8. ESI-HRMS, *m/z* for [**1** + PF_6]$^+$ 837.3728 (found), 837.3732 (calculated), relative error -0.5 ppm.

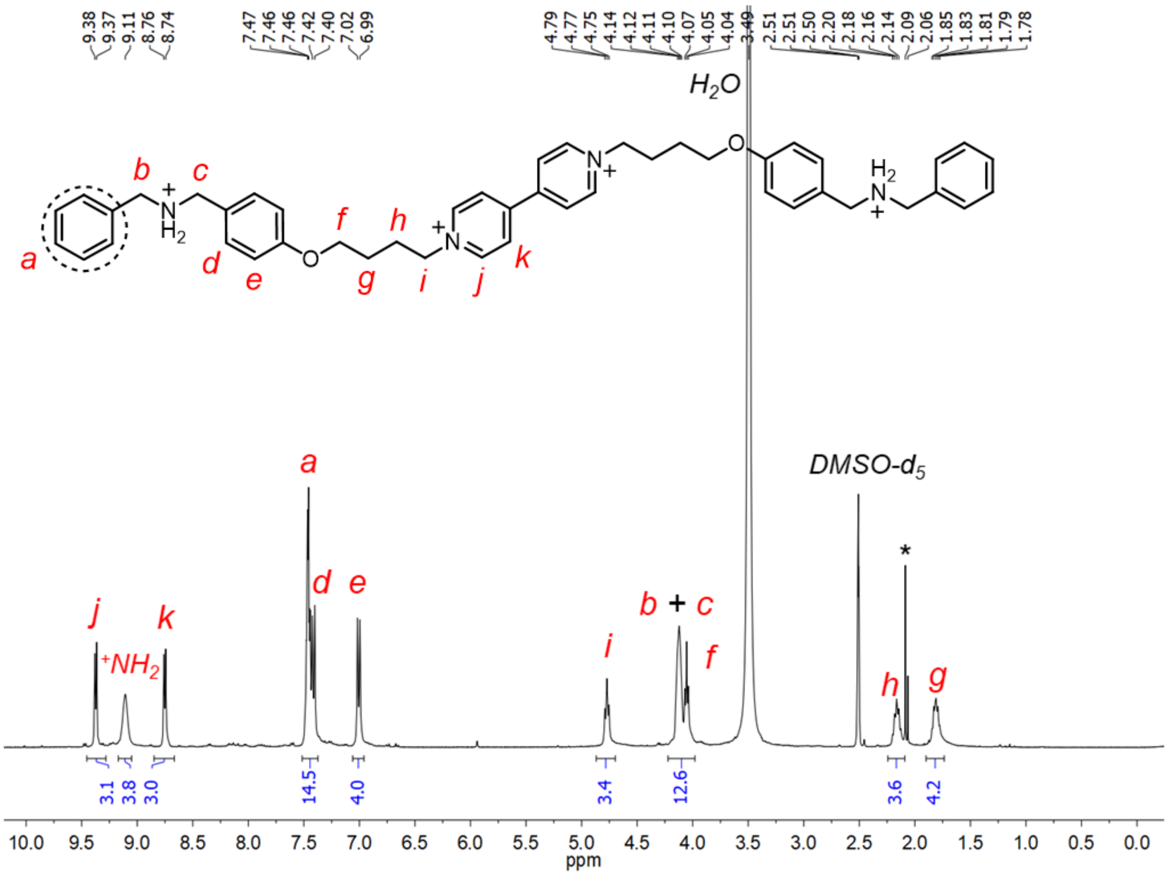

Figure S34. ^{1}H NMR spectrum (400 MHz, DMSO-d_6) of compound [**1**·H_2][PF_6]$_4$. * Denotes CH_3CN and $(CH_3)_2CO$.

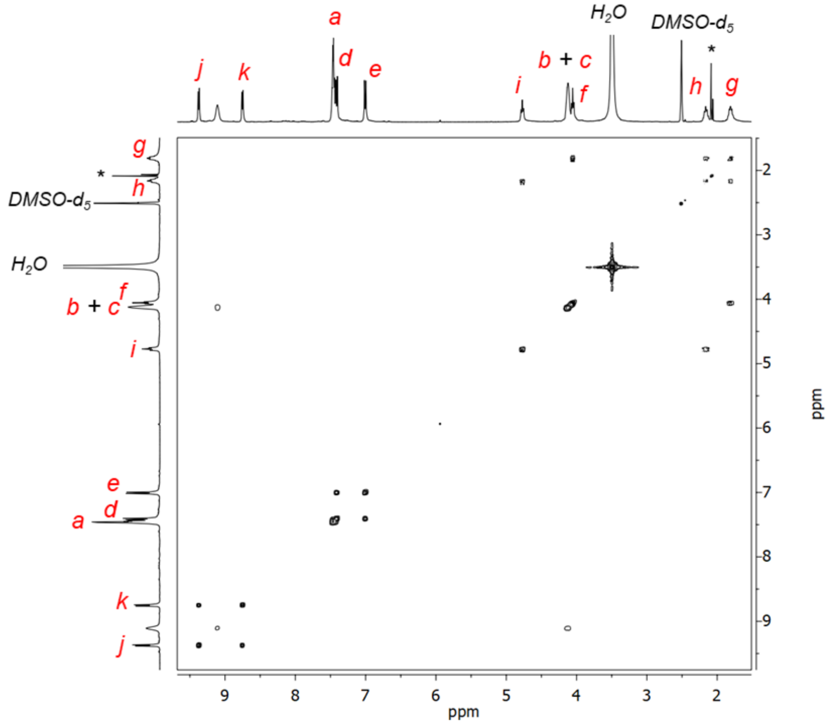

Figure S35. ^{1}H-^{1}H COSY NMR spectrum (400 MHz, DMSO-d_6) of salt [**1**·H_2][PF_6]$_4$. * Denotes CH_3CN and $(CH_3)_2CO$.

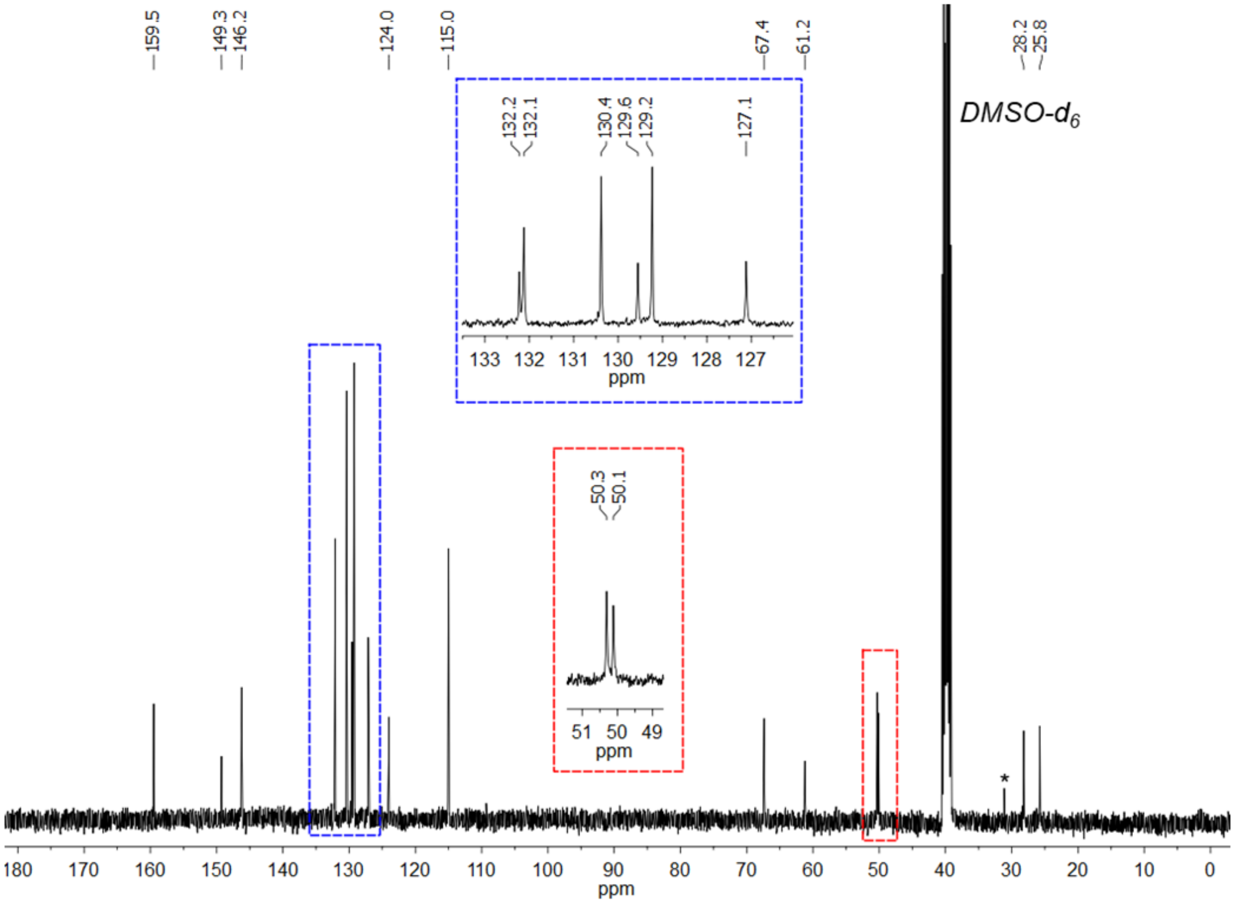

Figure S36. ^{13}C {^{1}H} NMR spectrum (100 MHz, DMSO-*d*$_6$) of compound [**1**·H_2][PF_6]$_4$. * Denotes $(CH_3)_2CO$.

[3]Rotaxane [1·H$_2$⊂(21C6)$_2$][PF$_6$]$_4$

A solution of **C-21** (159.5 mg, 0.48 mmol) and [**1**·H_2][PF_6]$_4$ (150.0 mg, 0.12 mmol), prepared in dry DCM (40 mL) and dry $MeNO_2$ (5 mL), was stirred for 15 min at room temperature under a N_2 atmosphere. Then, second-generation Grubbs' catalyst (29.5 mg, 0.048 mmol) was added and the resulting mixture was stirred for 15 min under same conditions, followed by 24 h of heating at 43 °C. After cooling the system to room temperature, ethyl vinyl ether was added (5 mL) and the mixture was stirred for 20 min followed by evaporation under reduced pressure. Column chromatography (SiO_2, MeOH:DCM, 2.5:7.5, R_f =0.2) afforded rotaxane [**1**·H_2⊂(**21C6**)$_2$][PF_6]$_4$ as a pale yellow solid (101.7 mg, 0.05 mmol, 45% yield). ^{1}H NMR (400 MHz, CD_3CN) δ_H 8.94 (d, J = 6.8 Hz, 4H), 8.40 (d, J = 6.7 Hz, 4H), 7.74 (br, 4H), 7.58–7.36 (m, 14H), 6.98 (d, J = 8.7 Hz, 4H), 5.99–5.74 (m, 4H), 4.72 (t, J = 7.6 Hz, 4H), 4.54–4.32 (m, 8H), 4.06 (t, J = 6.1 Hz, 4H), 4.02 (d, J = 5.6 Hz, 4H), 3.69 (s, 8H), 3.67–3.52 (m, 16H), 3.45–3.39 (m, 4H), 3.32–3.27 (m, 4H), 3.20–3.06

(m, 12H), 2.44–3.40 (m, J = 4.8 Hz, 4H), 2.25–2.18 (m, 4H), 1.92–1.85 (m, 4H); ^{13}C NMR (100 MHz, CD_3CN) δ_C 159.5, 150.1, 145.6, 136.8, 132.5, 132.0, 130.4, 129.2, 128.6, 127.8, 127.3, 124.7, 114.4, 72.2, 71.9, 71.6, 71.3, 71.1, 71.0, 70.5, 70.4, 70.3, 70.2, 70.0, 67.1, 61.9, 51.1, 50.7, 33.3, 28.0, 25.5. ESI-HRMS, *m/z* for [**1** + (2 × **21C6**) + 2H]$^{4+}$ 325.7016 (found), 325.6999 (calculated), relative error 5.2 ppm.

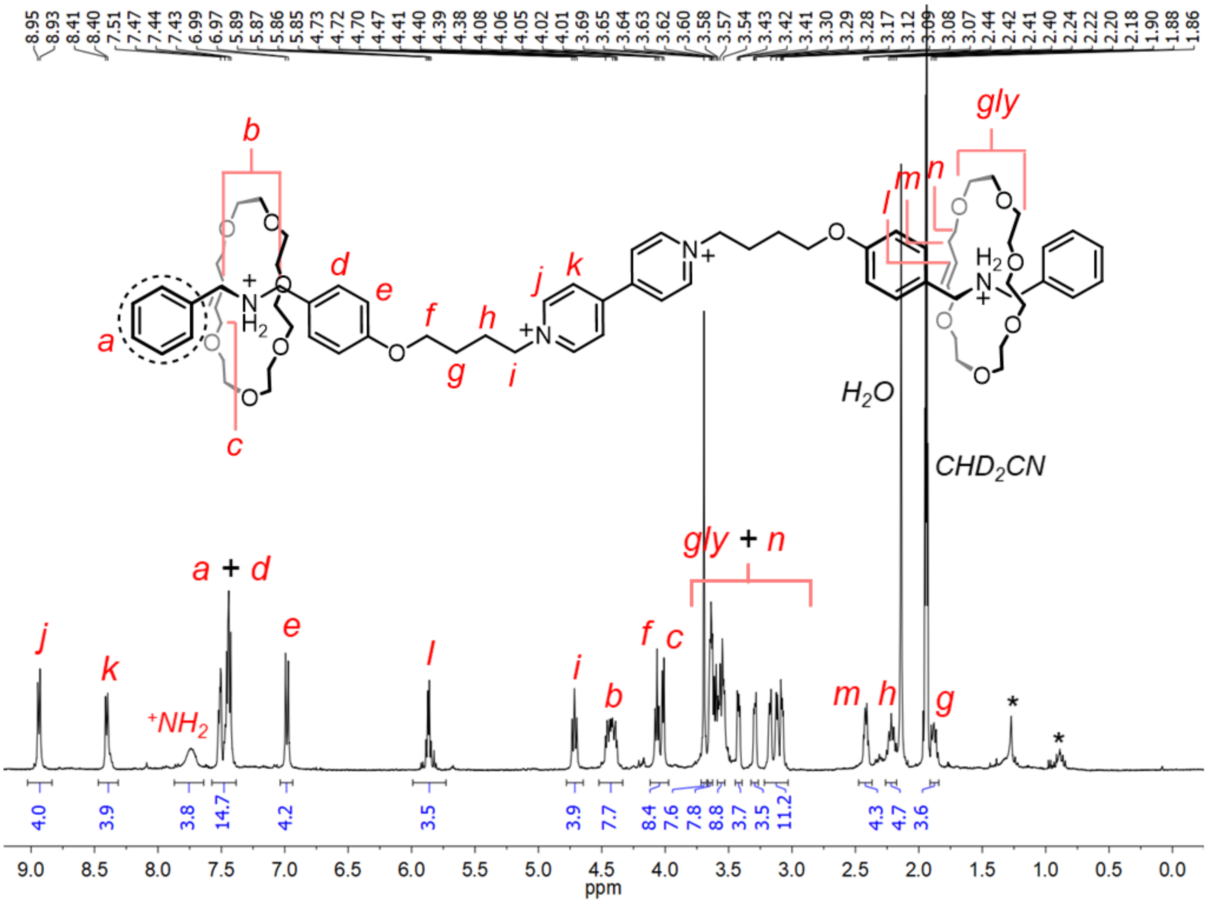

Figure S37. ^{1}H NMR spectrum (400 MHz, CD_3CN) of [3]rotaxane [**1**·H_2⊂(**21C6**)$_2$][PF_6]$_4$. * Denotes grease.

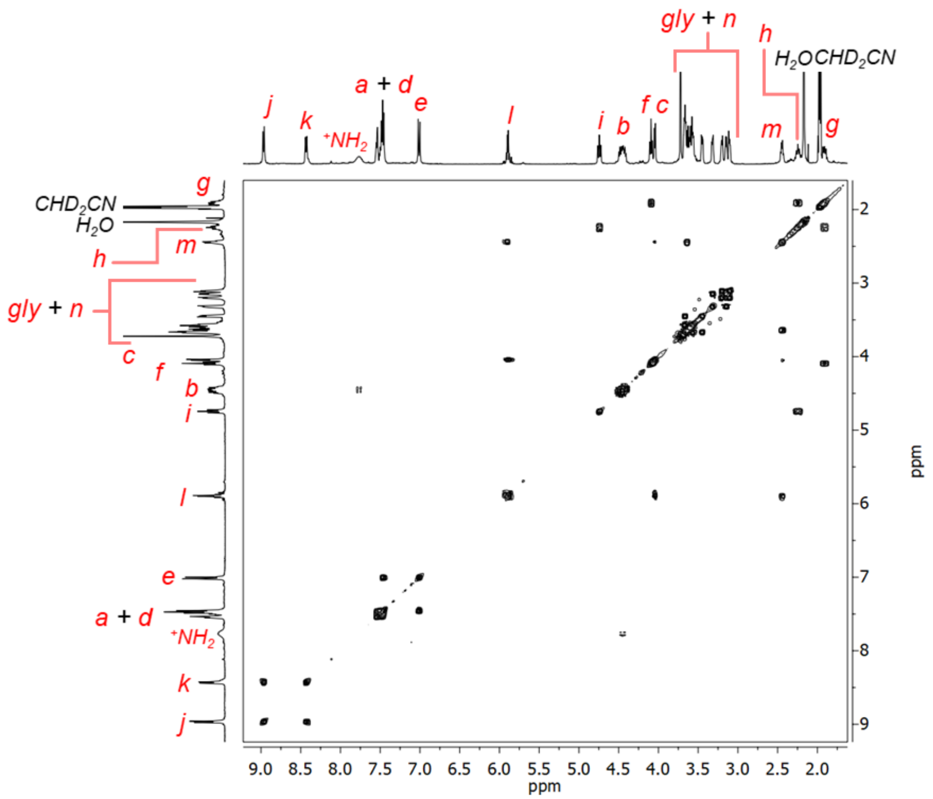

Figure S38. ^{1}H-^{1}H COSY NMR spectrum (400 MHz, CD_3CN) of compound [**1**·H_2⊂(**21C6**)$_2$][PF_6]$_4$.

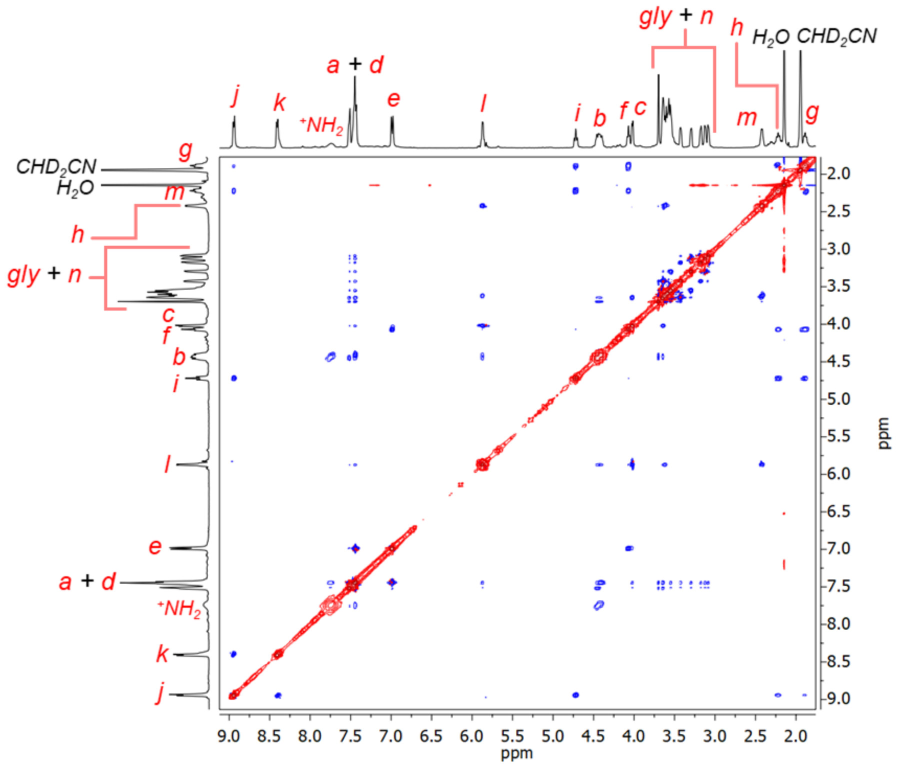

Figure S39. ^{1}H-^{1}H NOESY NMR spectrum (400 MHz, CD_3CN) of [3]rotaxane [**1**·H_2⊂(**21C6**)$_2$][PF_6]$_4$.

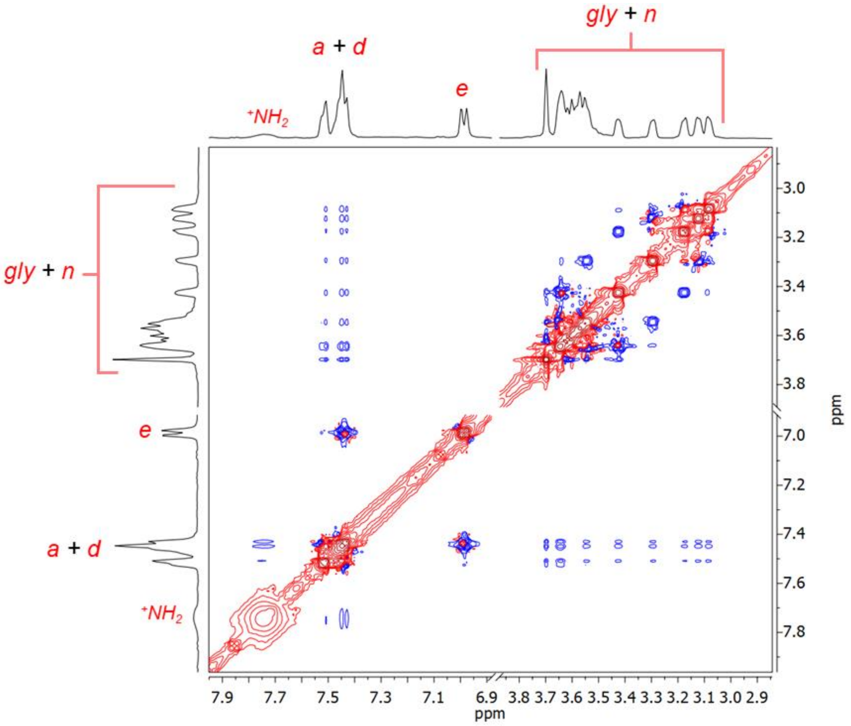

Figure S40 NOESY NMR spectrum of [**1**·H_2⊂(**21C6**)$_2$][PF_6]$_4$; cropped section showing the benzyl and glycol regions.

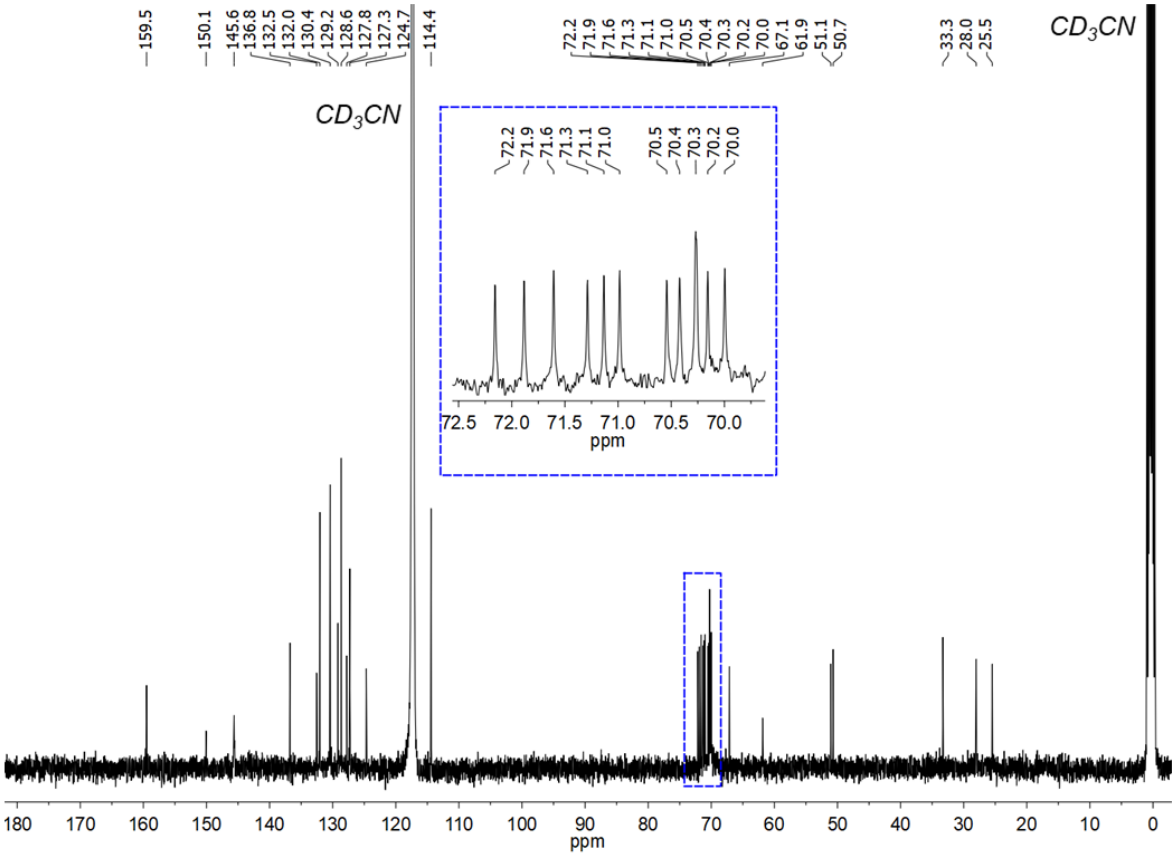

Figure S41. ^{13}C {^{1}H} NMR spectrum (100 MHz, CD_3CN) of rotaxane[**1**·H_2⊂(**21C6**)$_2$][PF_6]$_4$.

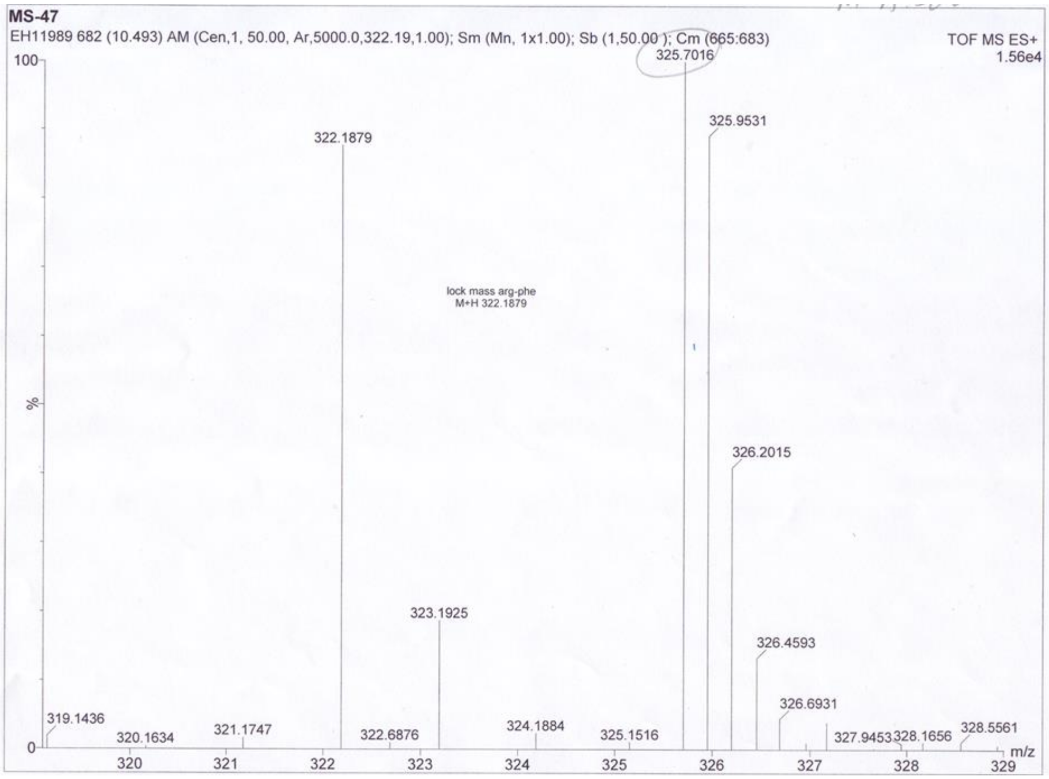

Figure S42.Partial HRMS of the isolated rotaxane [**1**·H_2⊂(**21C6**)$_2$][PF_6]$_4$.

General synthesis of the hetero[4]rotaxane structures

Macrocycle **DN32C8**, salt [**1**·H_2][PF_6]$_4$, and the corresponding glycol precursor (**C-21** or **C-22**) were all dissolved in MeCN (1 mL) and DCM (9 mL). The resulting red solution was stirred for 15 min followed by rotary evaporation (T < 30 ºC) to dry the product. The residue was then dissolved under N_2 atmosphere in dry CH_2Cl_2 (23 mL) and dry $MeNO_2$ (3 mL). After stirring for 5 min at room temperature, second-generation Grubbs' catalyst was added, and stirring was continued for 10 min. The reaction mixture was heated at 43 °C for 36 h and then cooled to room temperature to be subsequently quenched with ethyl vinyl ether (5 mL) and stirred for 20 min. After removing all solvents by rotary evaporation (T < 30 °C) the hetero[4]rotaxane species, ***p*-H[4]R** and ***t*-H[4]R**, were purified by column chromatography (SiO_2, MeOH:DCM, 2.5:7.5).

***p*-H[4]R**: compound **C-21** (85.1 mg, 0.26 mmol); macrocycle **DN32C8** (131.6 mg, 0.24 mmol); salt [**1**·H_2][PF_6]$_4$ (80.0 mg, 0.06 mmol), Grubbs' catalyst (16.0 mg, 0.03 mmol). R_f = 0.24, red crystalline solid, 37% yield (43.8 mg, 0.02 mmol). ^{1}H NMR (400 MHz, CD_3CN) δ_H H_j (4H) observed as $H_{j/j'}$ at 8.41 (d, J = 6.4 Hz) and 8.37 (d, J = 6.4 Hz), 7.70 (br, 4H), 7.50–7.40 (m, 14H), H_k (4H) observed as $H_{k/k'}$ at 7.30 (d, J = 6.6 Hz) and 7.14 (d, J = 6.4 Hz), 7.07 (d, J = 6.8 Hz, 4H), H_o (4H) observed as $H_{o/o'}$ at 7.00 (d, J = 8.3 Hz) and 6.79 (d, J = 8.3 Hz), H_p observed as $H_{p/p'}$ at 6.91–6.86 (m, 4H), H_q (4H) observed as $H_{q/q'}$ at 6.66 (d, J = 7.5 Hz) and 6.42 (d, J = 7.6 Hz), 5.81–5.78 (m, 4H), H_i (4H) observed as $H_{i/i'}$ at 4.59 (t, J = 7.7 Hz) and 4.52–4.45 (br), 4.44–4.32 (m, 8H), 4.22 (t, J = 5.9 Hz, 4H), 4.14–3.79 (m, 28H), 3.72–3.46 (m, 28H), 3.39–3.35 (m, 4H), 3.25–3.22 (m, 4H), 3.17–2.97 (m, 12H), 2.38–2.35 (m, 4H), 2.26 (br, 4H), 2.07–2.01 (m, 4H); ^{13}C NMR (100 MHz, CD_3CN) δ_C 159.5, 153.3, 153.0, 144.1, 144.0, 143.9, 143.8, 136.7, 132.5, 132.1, 130.4, 129.1, 128.6, 127.7, 125.7, 125.5, 125.3, 124.71, 124.66, 124.4, 123.7, 114.4, 113.5, 105.3, 105.1, 72.1, 71.8, 71.6, 71.2, 71.1, 70.9, 70.7, 70.5, 70.4, 70.2, 70.1, 69.94, 69.91, 69.89, 67.8, 67.3, 67.2, 61.2, 51.0, 50.6, 33.3, 28.2, 28.1, 25.9. UV-vis (MeCN): λ_{max} (ε) 475 nm (1520 mol^{-1} dm^3 cm^{-1}). ESI-HRMS, m/z for [**1** + **DN32C8** + (2 × **21C6**) + 2H]$^{4+}$ 462.7604 (found), 462.7607 (calculated), relative error -0.7 ppm.

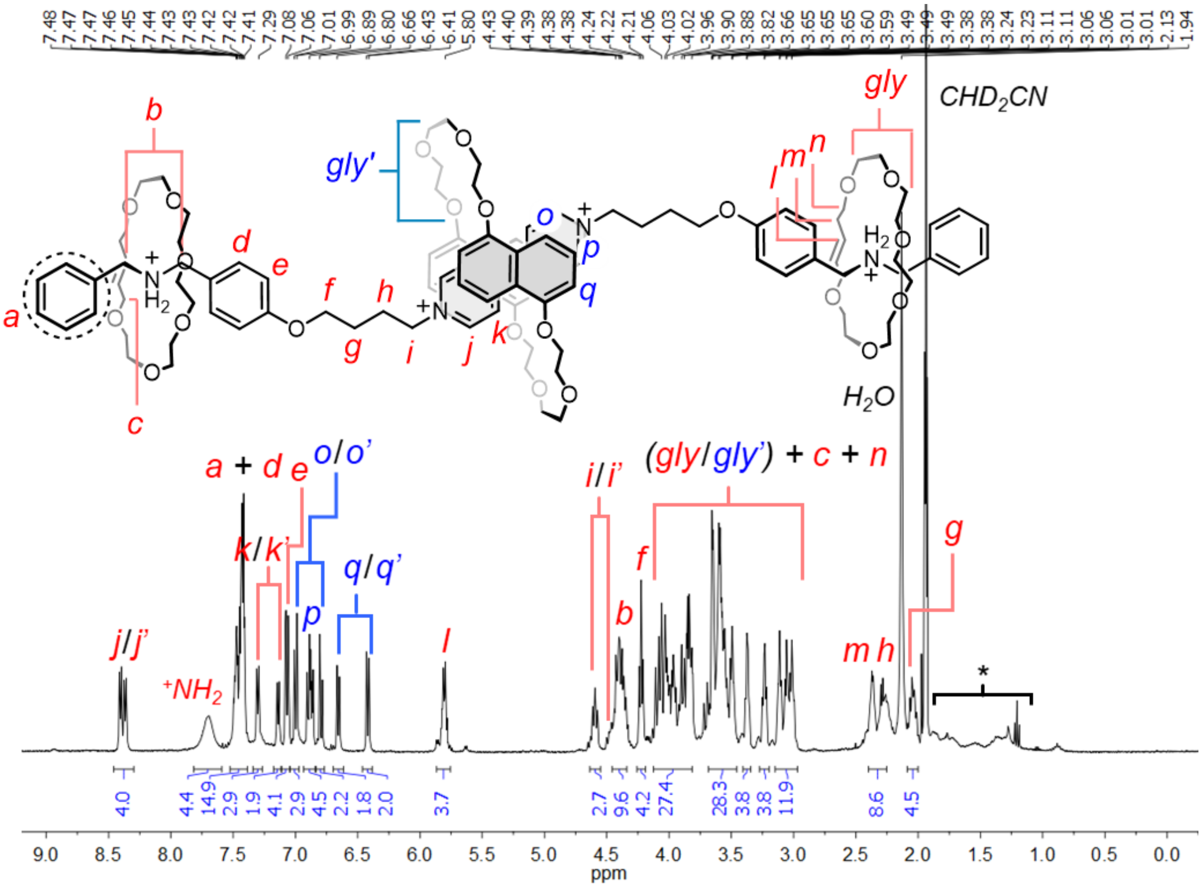

Figure S43. ^{1}H NMR spectrum (400 MHz, CD_3CN) of hetero[4]rotaxane ***p*-H[4]R**. * Denotes hexanes and AcOEt.

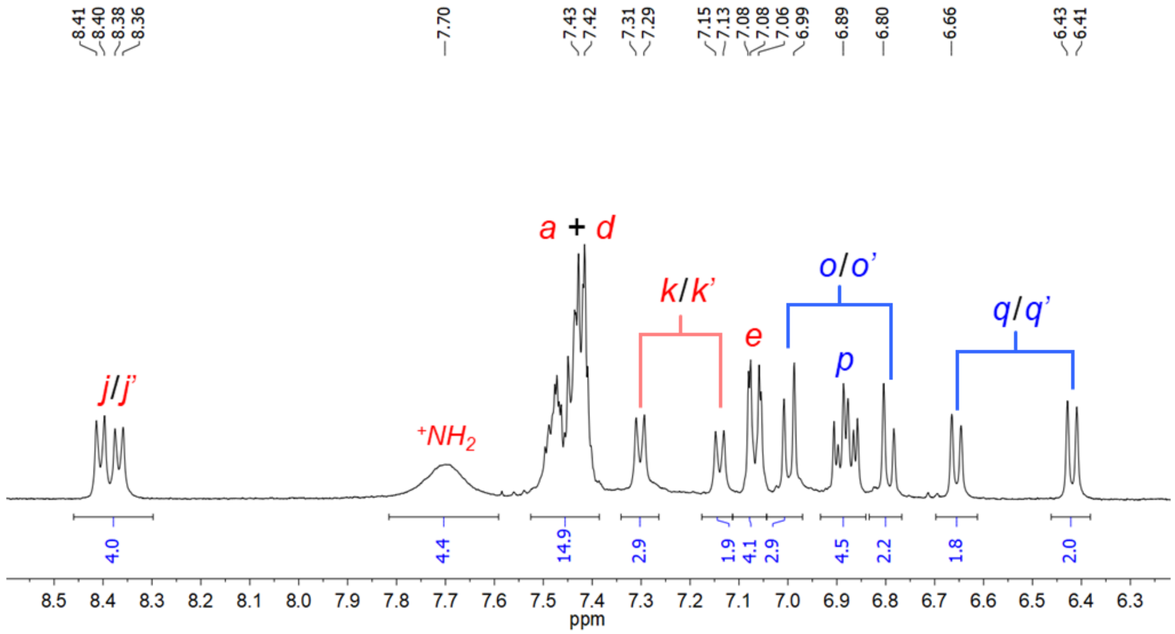

Figure S44. Partial ^{1}H NMR spectrum (400 MHz, CD_3CN) of hetero[4]rotaxane ***p*-H[4]R**.

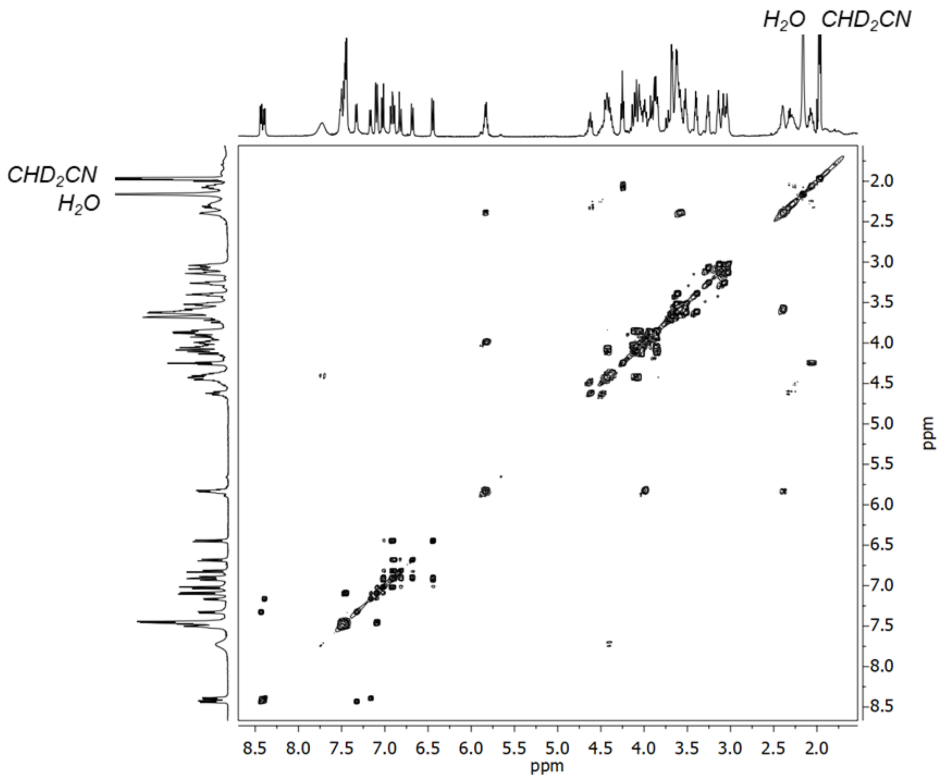

Figure S45. ^{1}H-^{1}H COSY NMR spectrum (400 MHz, CD_3CN) of rotaxane ***p*-H[4]R**.

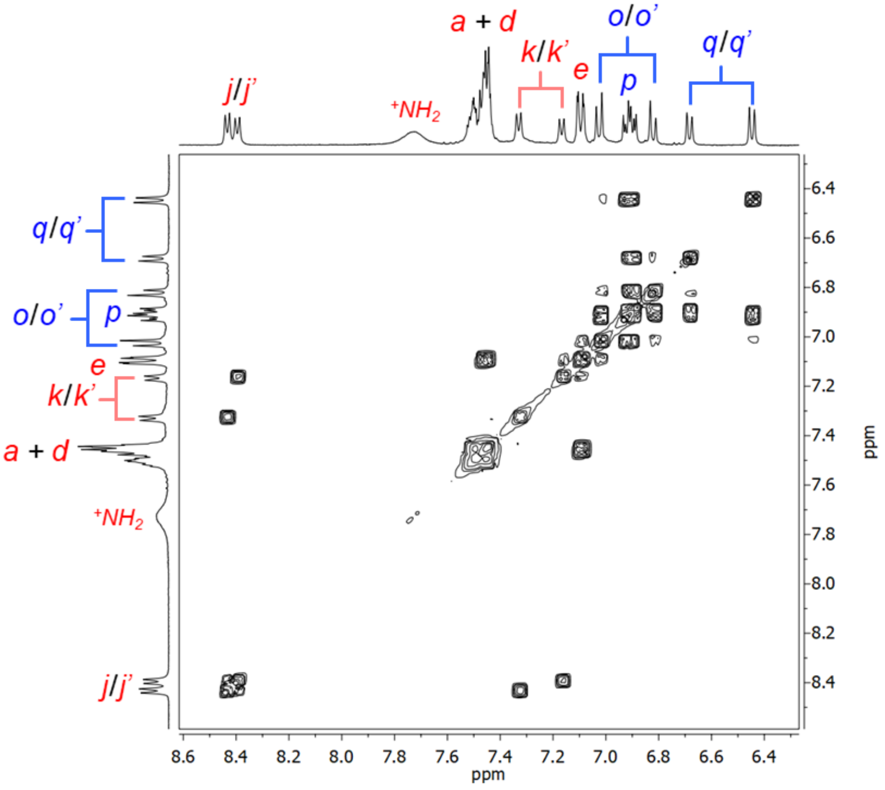

Figure S46. Partial (aromatic region) COSY NMR spectrum (400 MHz, CD_3CN) of ***p*-H[4]R**.

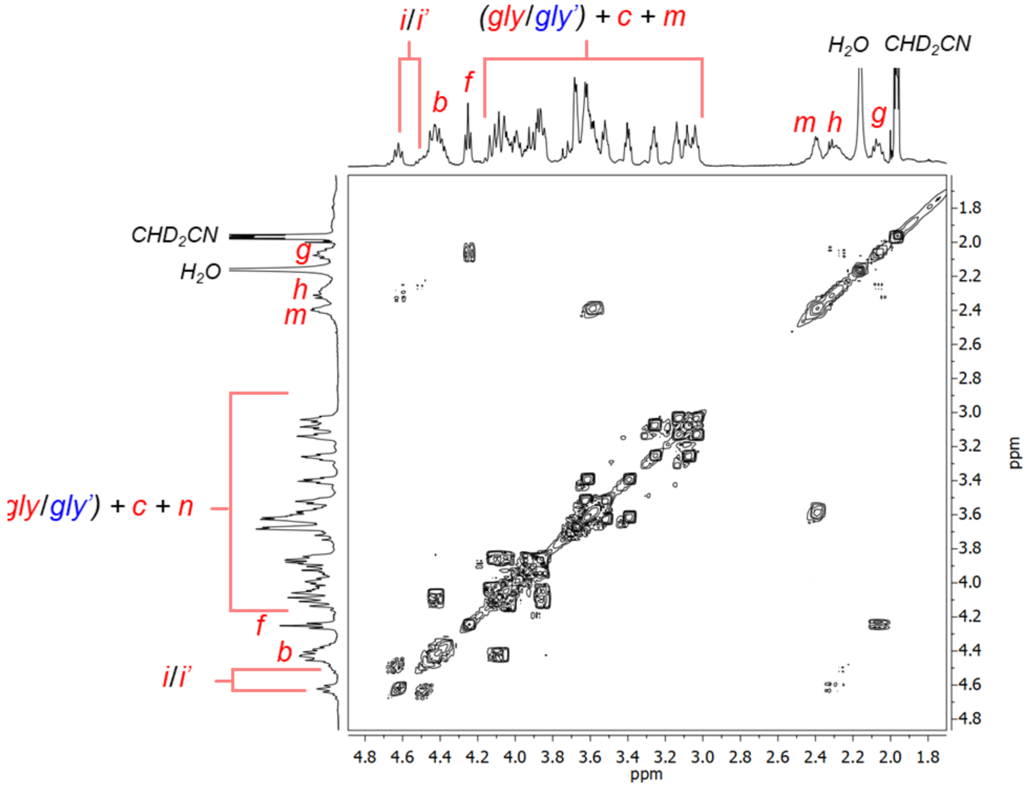

Figure S47. Partial (aliphatic/glycol regions) ^{1}H-^{1}H COSY NMR spectrum (400 MHz, CD_3CN) of rotaxane ***p*-H[4]R**.

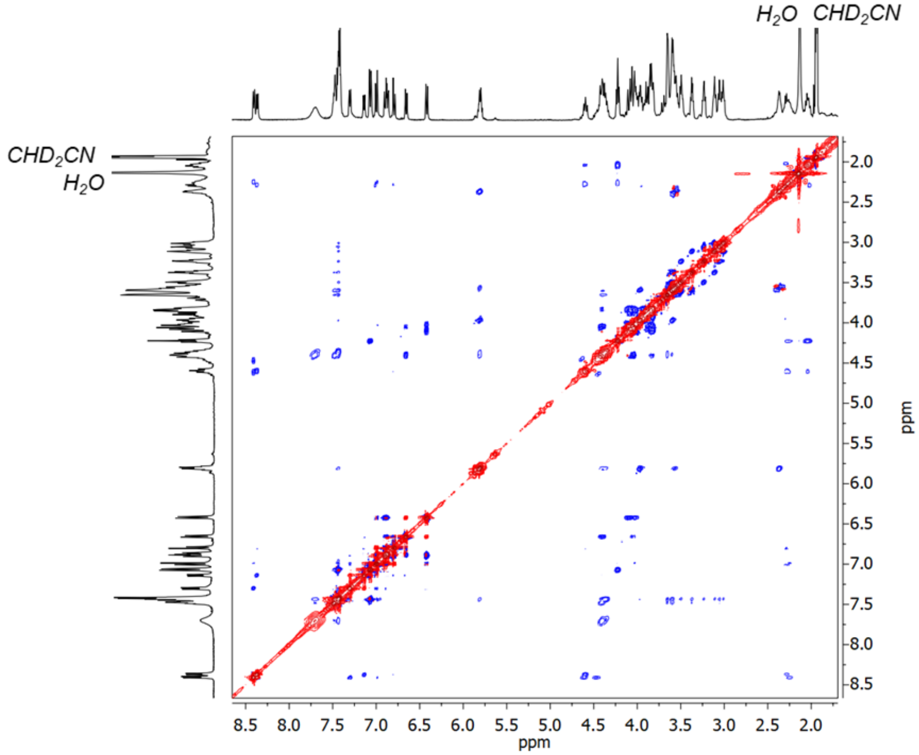

Figure S48. ^{1}H-^{1}H NOESY NMR spectrum (400 MHz, CD_3CN) of compound ***p*-H[4]R**.

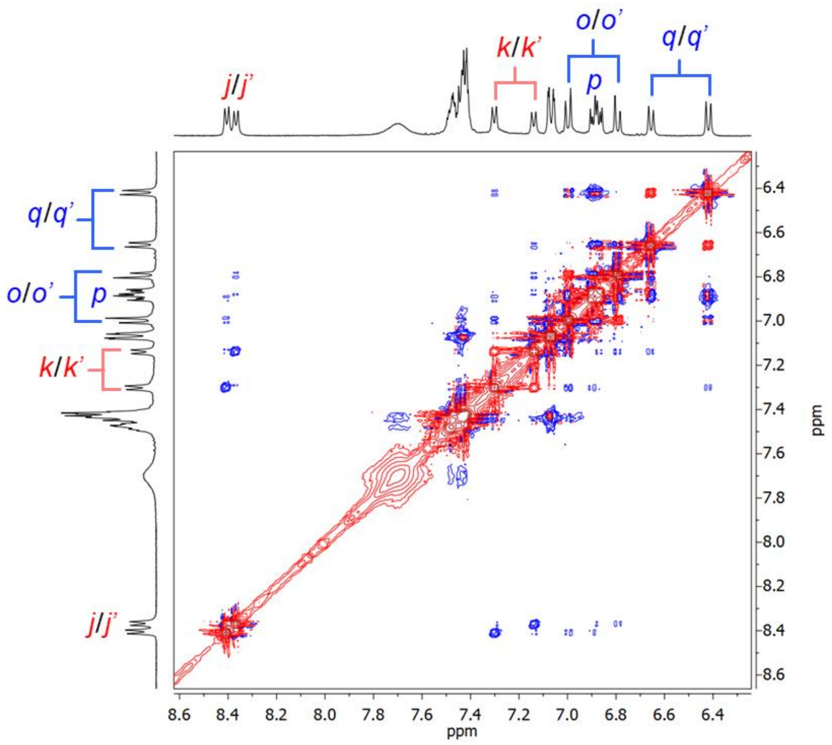

Figure S49. NOESY NMR spectrum of ***p*-H[4]R**; zoom-in showing bipyridinium–naphthalene through-space couplings.

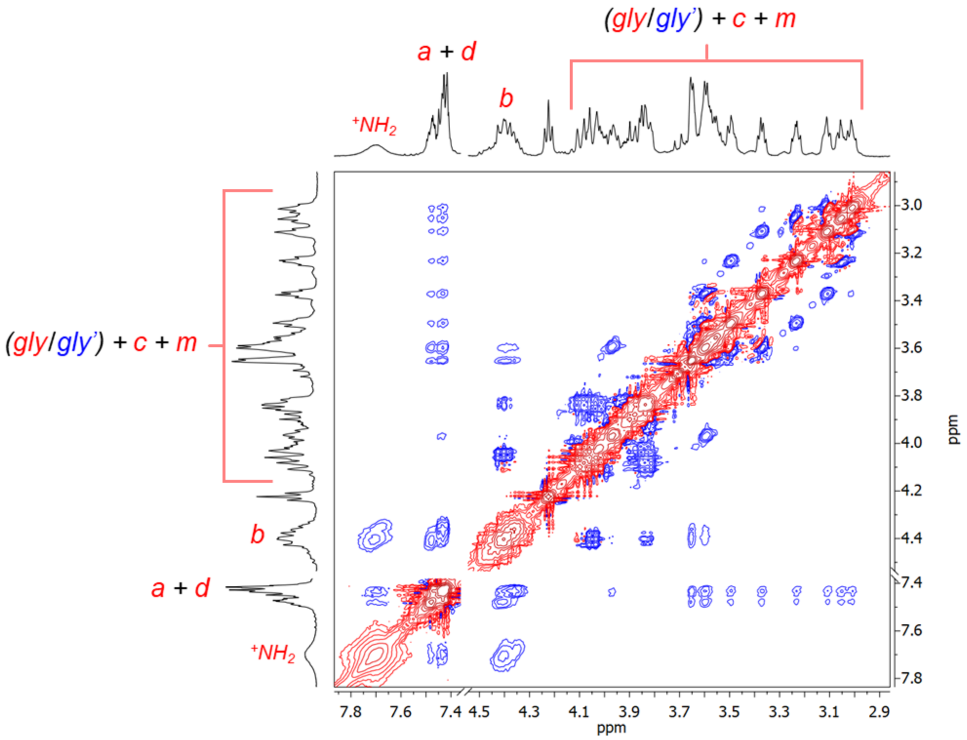

Figure S50. NOESY NMR spectrum of ***p*-H[4]R**; cropped section showing benzyl–glycol through-space couplings.

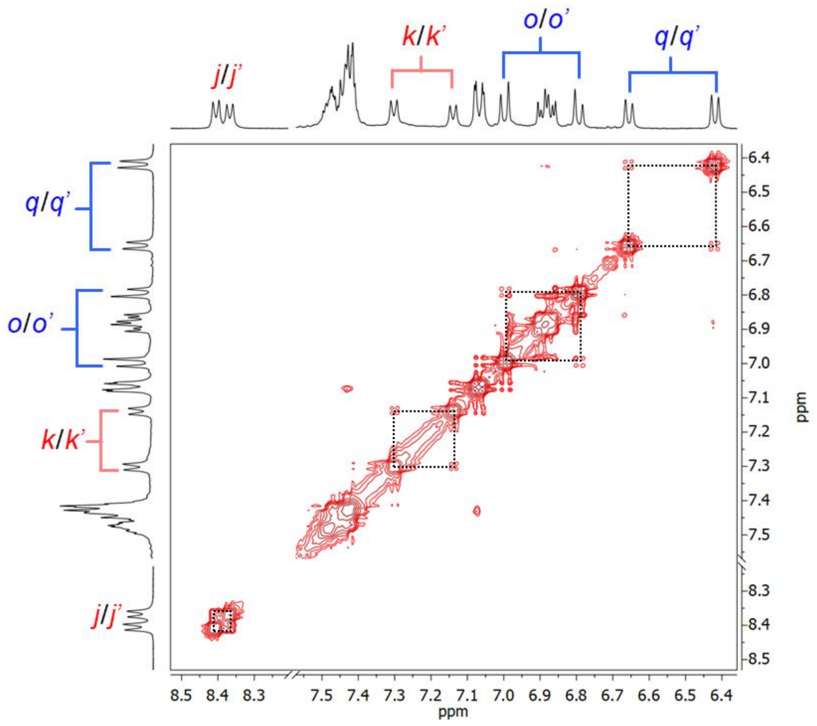

Figure S51. Partial ^{1}H-^{1}H EXSY NMR spectrum (400 MHz, CD_3CN) of ***p*-H[4]R**.

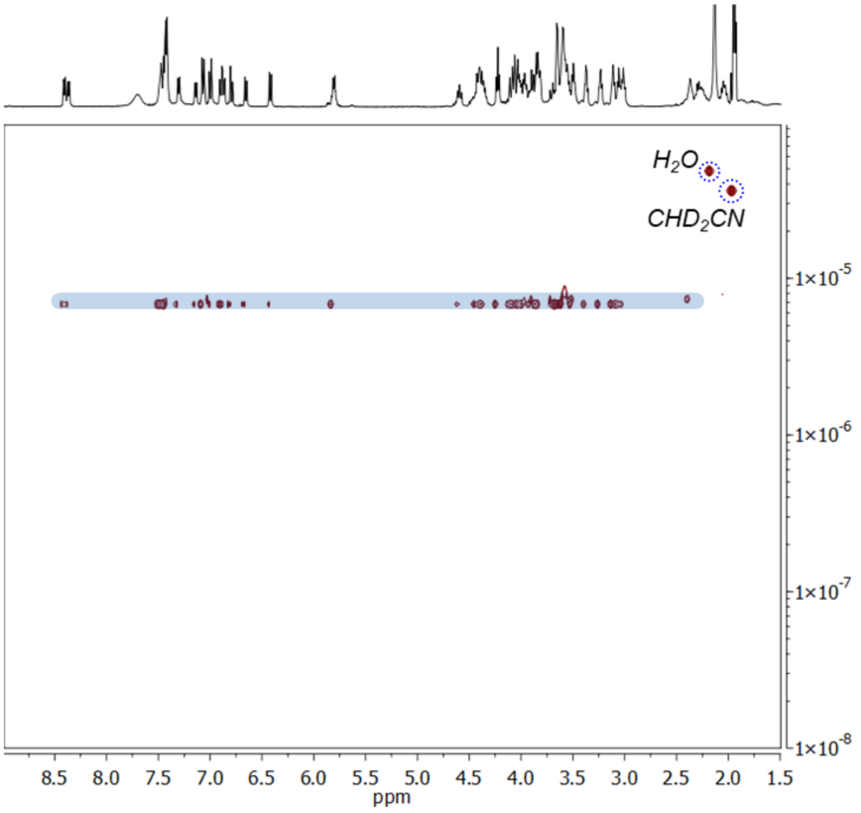

Figure S52 ^{1}H DOSY NMR spectrum (100 MHz, CD_3CN, 25 ºC) of hetero[4]rotaxane ***p*-H[4]R**.

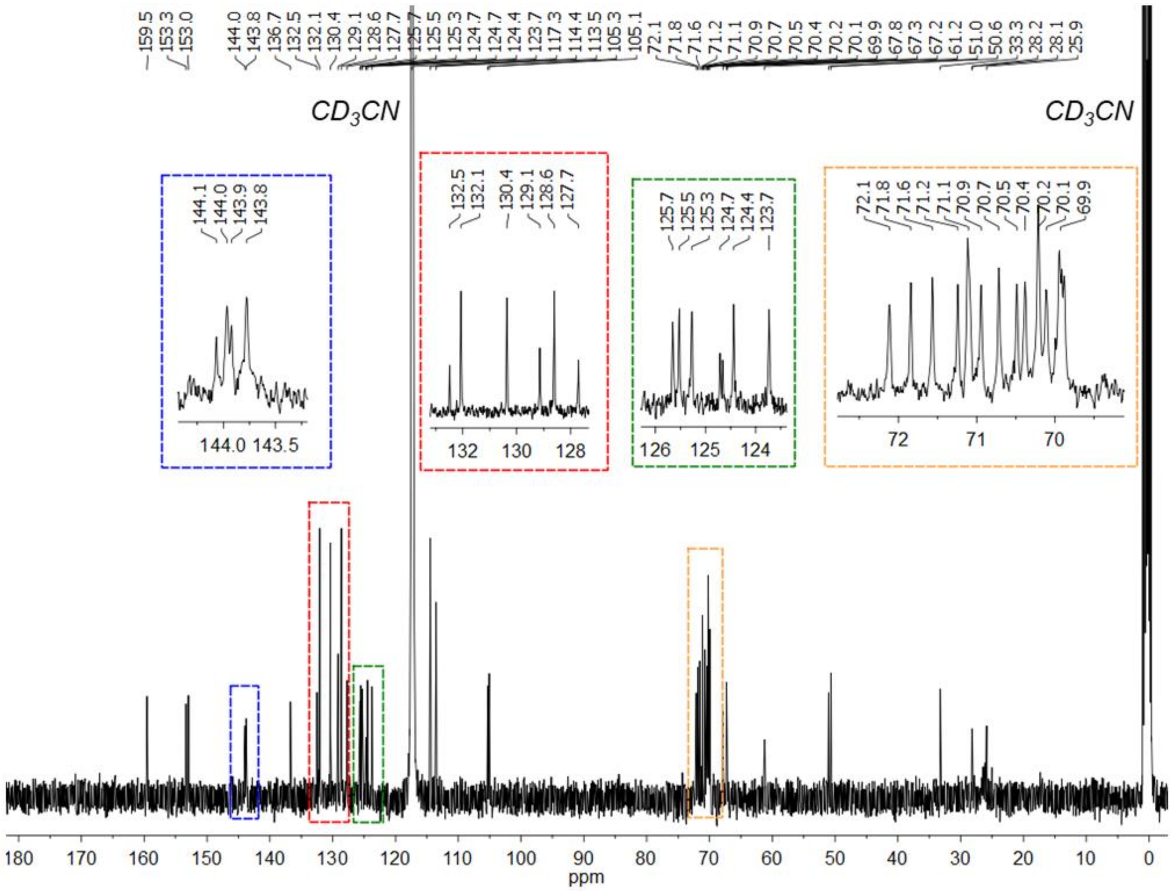

Figure S53. ^{13}C {^{1}H} NMR spectrum (100 MHz, CD_3CN) of hetero[4]rotaxane ***p*-H[4]R**.

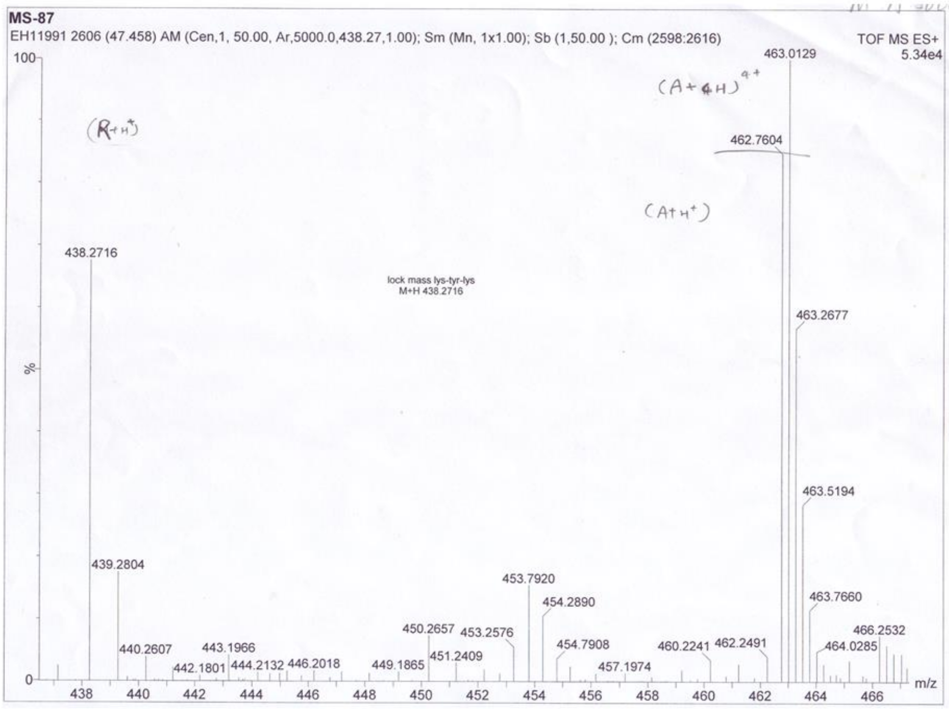

Figure S54. Partial HRMS showing the parent ion [**1** + **DN32C8** + (2 × **21C6**) + 2H]$^{4+}$ for rotaxane ***p*-H[4]R**.

***t*-H[4]R**: compound **C-22** (103.9 mg, 0.30 mmol); macrocycle **DN32C8** (159.1 mg, 0.29 mmol); salt [**1**·H_2][PF_6]$_4$ (95.0 mg, 0.07 mmol), Grubbs' catalyst (16.0 mg, 0.03 mmol). R_f = 0.24, red crystalline solid, 30% yield (54.6 mg, 0.02 mmol). ^{1}H NMR (400 MHz, CD_3CN) δ_H H_i (4H) observed as $H_{i/i'}$ at 8.43 (d, *J* = 6.5 Hz) and 8.39 (d, *J* = 6.3 Hz), 7.62 (s, 4H), 7.53–7.44 (m, 14H), H_j (4H) observed as $H_{j/j'}$ at 7.32 (d, *J* = 6.4 Hz) and 7.16 (d, *J* = 6.3 Hz), 7.11 (d, *J* = 7.0 Hz, 4H), H_n (4H) observed as $H_{n/n'}$ at 7.02 (d, *J* = 8.4 Hz) and 6.82 (d, *J* = 8.3 Hz), H_o observed as $H_{o/o'}$ at 6.94–6.89 (m, 4H), H_p (4H) observed as $H_{p/p'}$ at 6.68 (d, *J* = 7.6 Hz) and 6.45 (d, *J* = 7.6 Hz), 5.57–5.52 (m, 4H), H_h (4H) observed as $H_{h/h'}$ at 4.62 (t, *J* = 7.7 Hz) and 4.53–4.49 (m), 4.48–4.37 (m, 8H), 4.25 (t, *J* = 5.9 Hz, 4H), 4.15–4.02 (m, 10H), 3.95–3.83 (m, 12H), 3.69–3.56 (m, 32H), 3.48–3.40 (m, 8H), 3.25–3.20 (m, 8H), 3.06–2.97 (m, 8H), 2.38–2.23 (m, 16H), 2.10–2.03 ; ^{13}C NMR (100 MHz, CD_3CN) δ_C 159.6, 153.4, 153.0, 144.1, 144.0, 143.9, 143.8, 132.3, 130.0, 130.5, 130.2, 130.0, 129.2, 128.7, 125.7, 125.5, 125.3, 124.4, 123.7, 114.5, 113.5, 105.3, 105.1, 71.7, 71.5, 71.4, 71.1, 70.9, 70.8, 70.7, 70.5, 70.4, 70.1, 69.9, 67.8, 67.3, 67.2, 61.3, 61.2, 52.1, 51.8, 51.7, 51.4, 32.7, 28.2, 28.1, 25.9. UV-vis (MeCN): λ_{max} (ε) 475 nm (1520 mol^{-1} dm^3 cm^{-1}). ESI-HRMS, *m/z* for [**1** + **DN32C8** + (2 × **22C6**) + (2 × PF_6) + 2H]$^{2+}$ 1084.9943 (found), 1085.0024 (calculated), relative error -7.5 ppm.

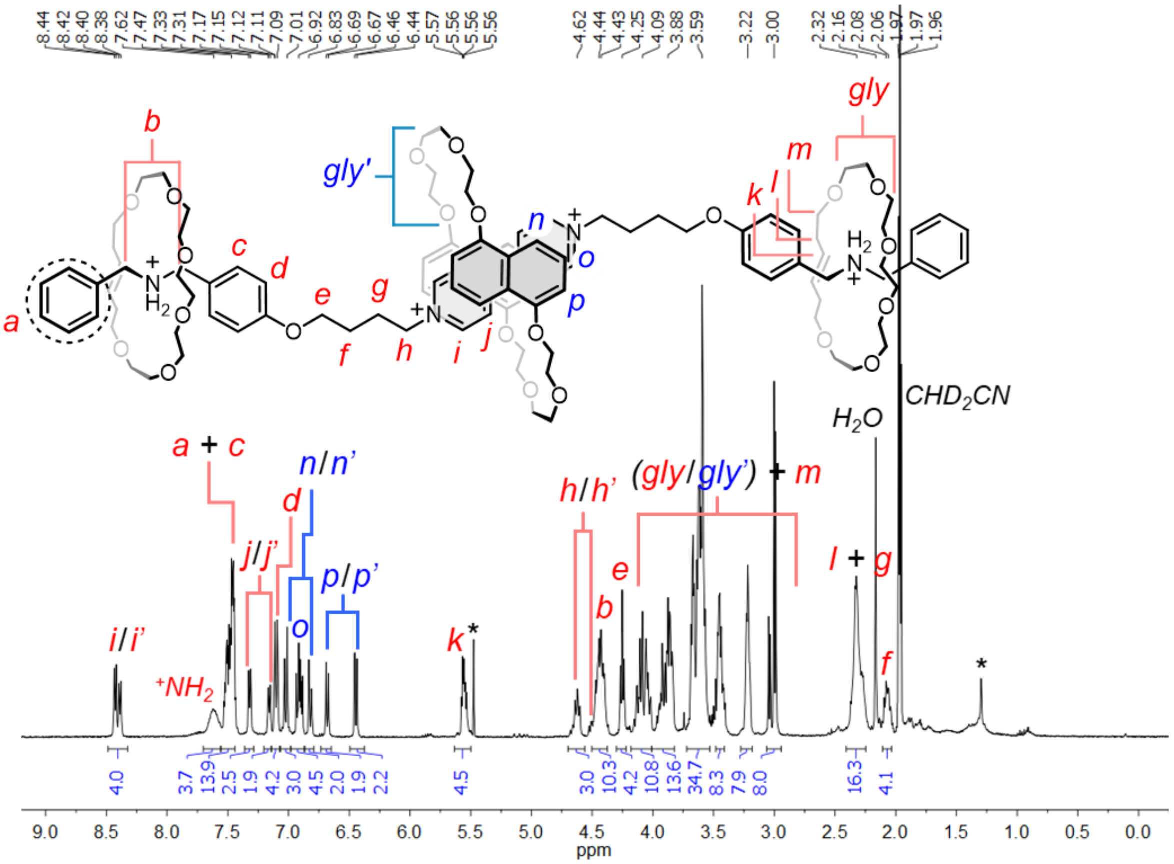

Figure S55. ^{1}H NMR spectrum (400 MHz, CD_3CN) of hetero[4]rotaxane ***t*-H[4]R**. * Denotes CH_2Cl_2 and hexanes.

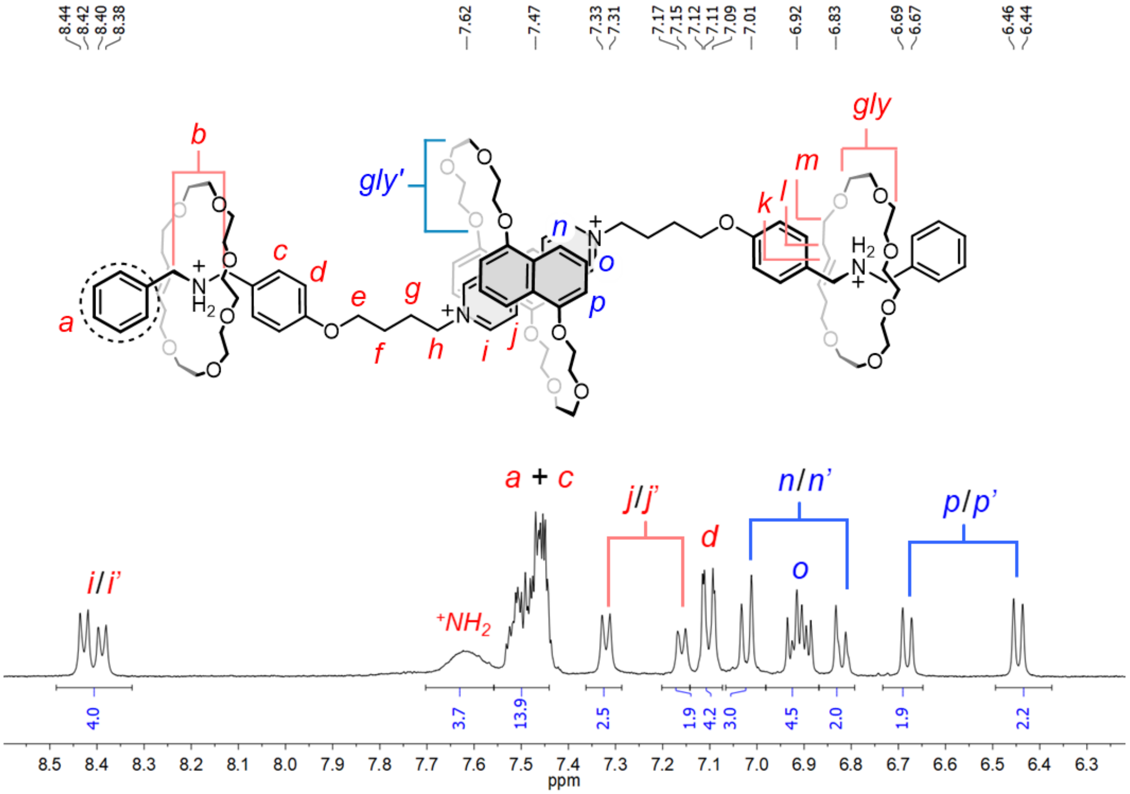

Figure S56. Partial ^{1}H NMR spectrum (400 MHz, CD_3CN) of hetero[4]rotaxane ***t*-H[4]R**.

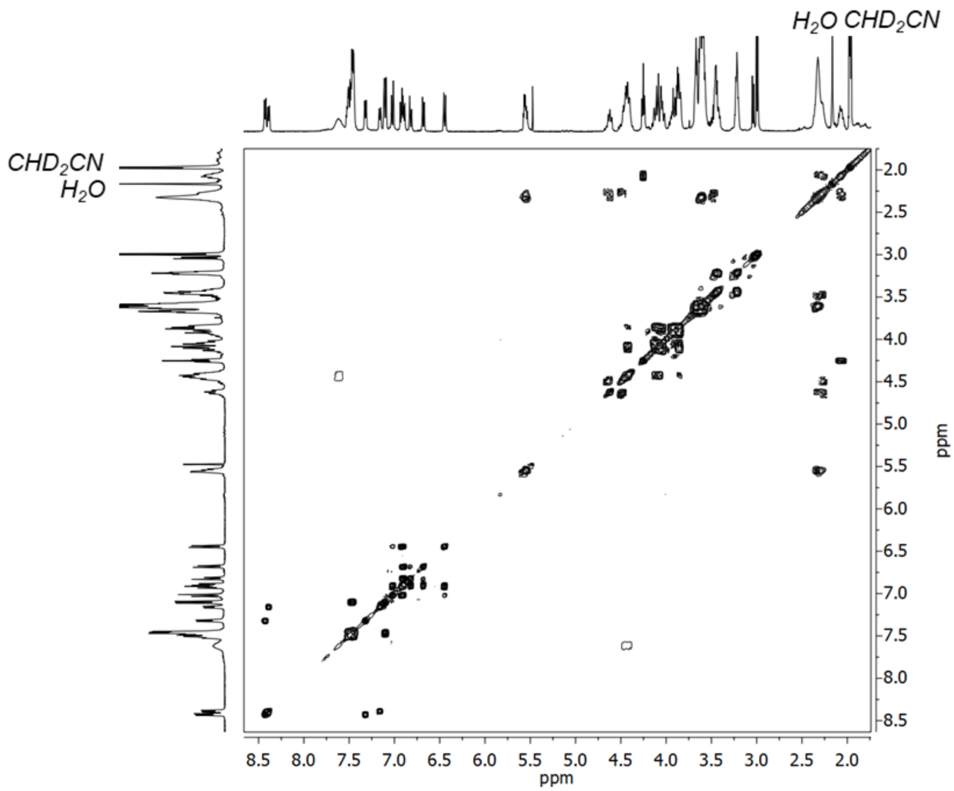

Figure S57. ^{1}H-^{1}H COSY NMR spectrum (400 MHz, CD_3CN) of rotaxane ***t*-H[4]R**.

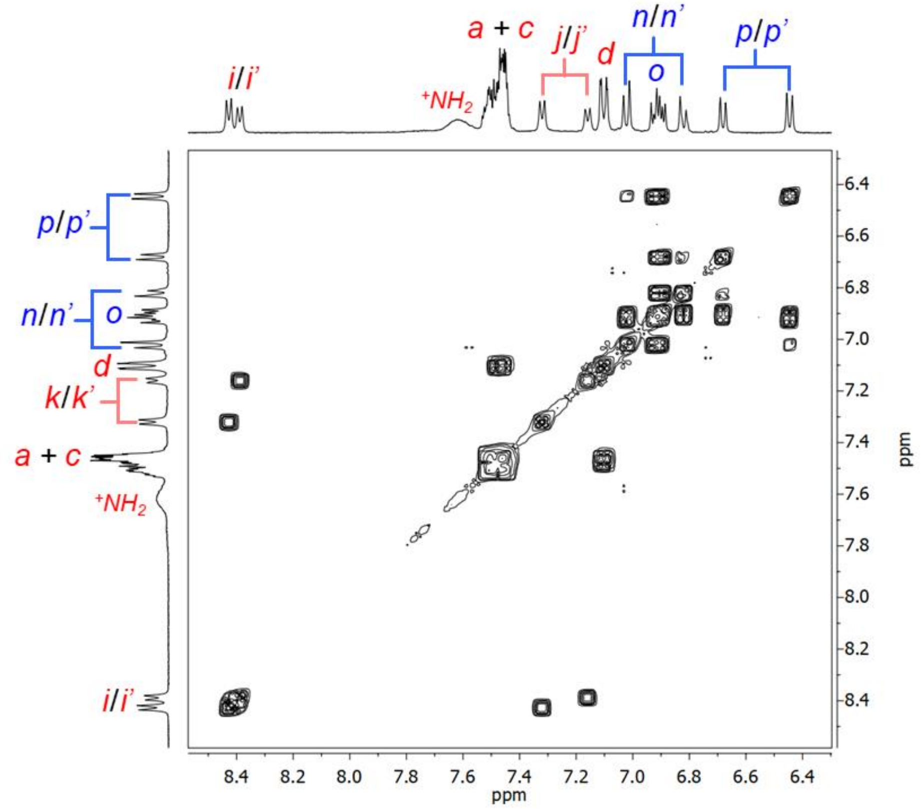

Figure S58. Partial (aromatic region) COSY NMR spectrum (400 MHz, CD_3CN) of ***t*-H[4]R**.

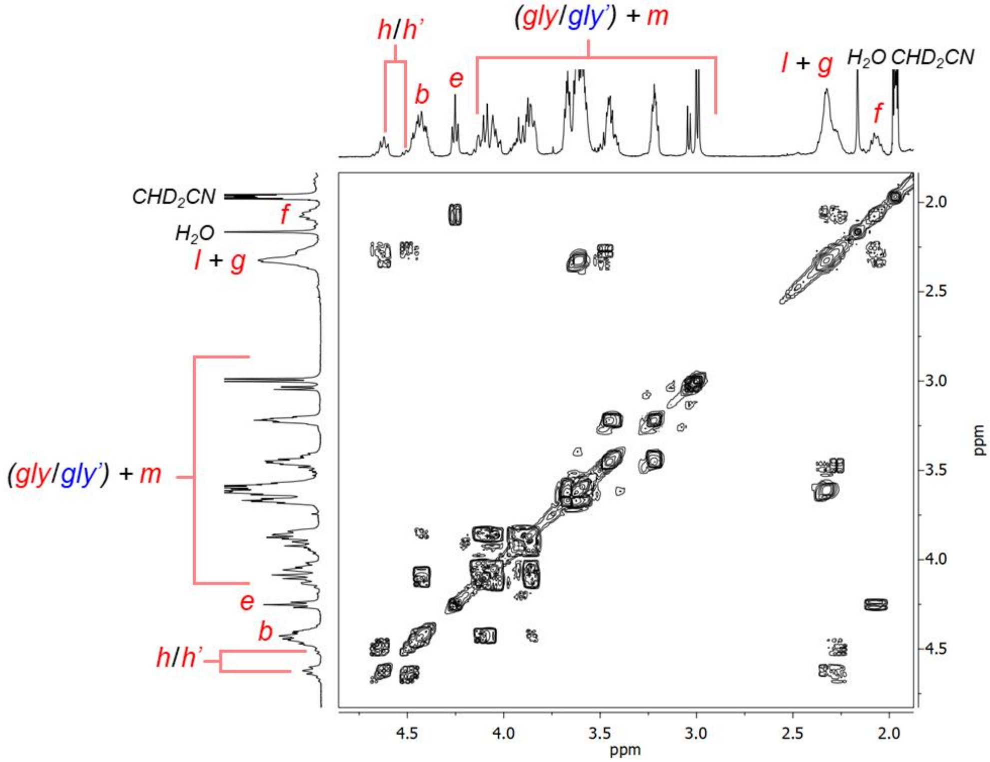

Figure S59. Partial (aliphatic/glycol regions) COSY NMR spectrum (400 MHz, CD_3CN) of rotaxane ***t*-H[4]R**.

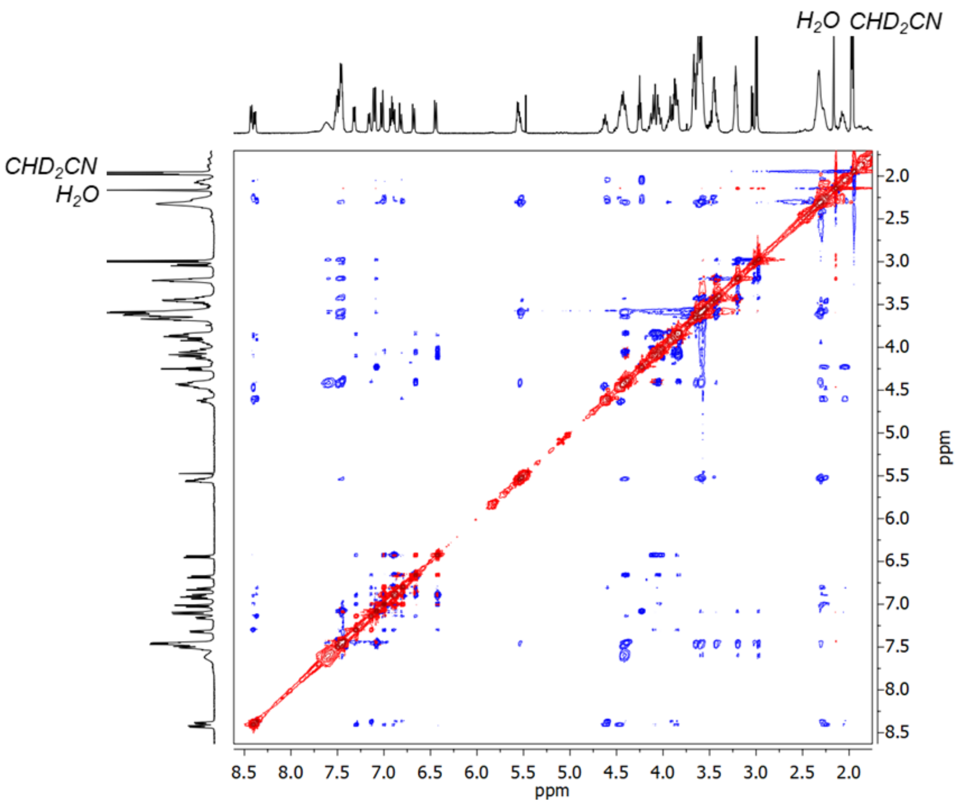

Figure S60. ^{1}H-^{1}H NOESY NMR spectrum (400 MHz, CD_3CN) of compound ***t*-H[4]R**.

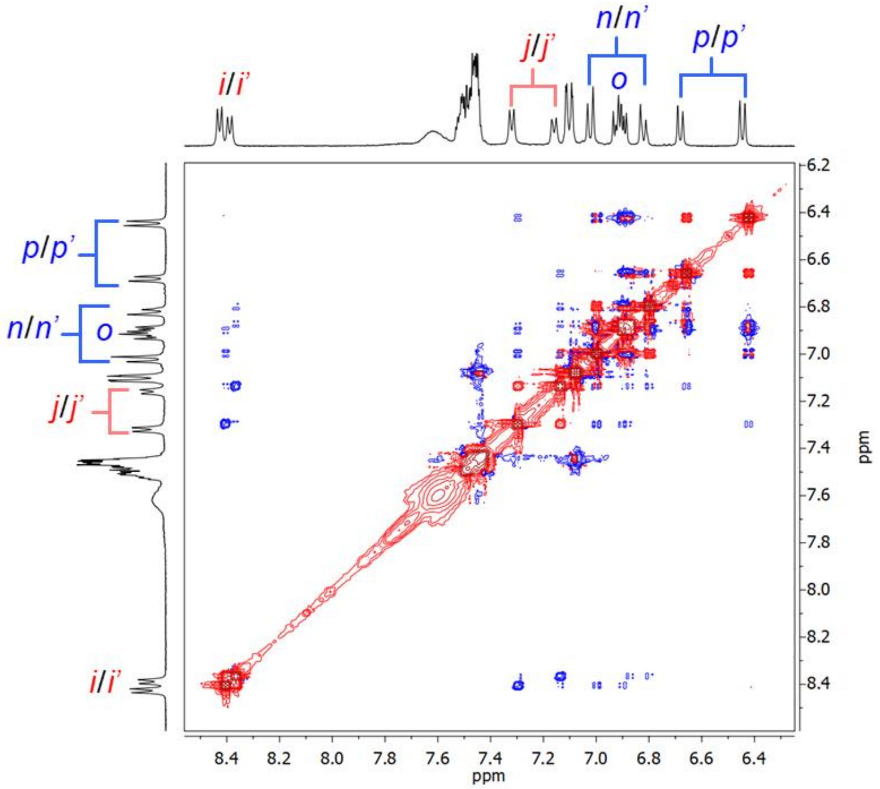

Figure S61. NOESY NMR spectrum of ***t*-H[4]R**; zoom-in showing bipyridinium–naphthalene through-space couplings.

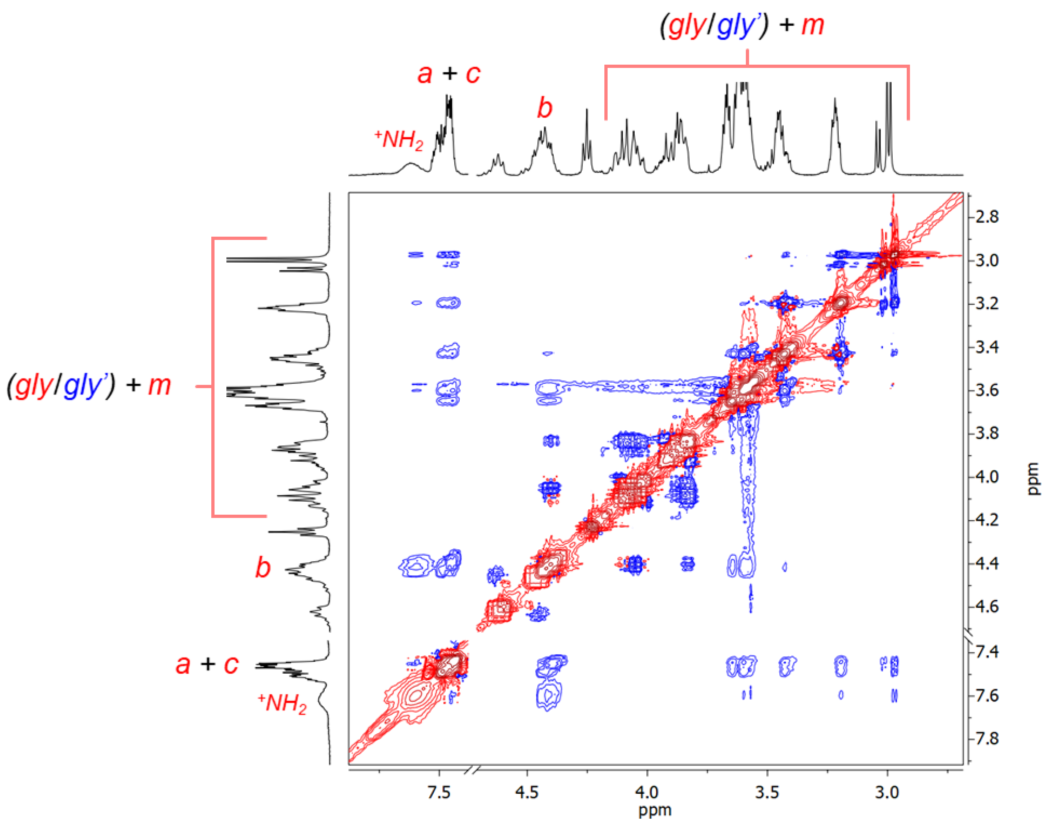

Figure S62. NOESY NMR spectrum of ***t*-H[4]R**; cropped section showing benzyl–glycol through-space couplings.

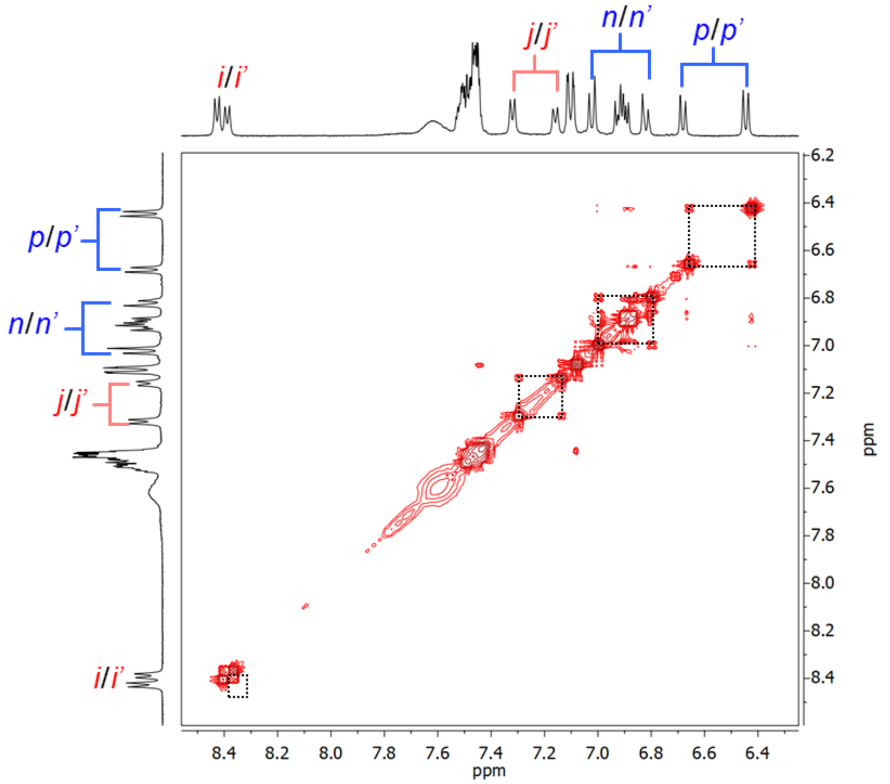

Figure S63. Partial ^{1}H-^{1}H EXSY NMR spectrum (400 MHz, CD_3CN) of ***t*-H[4]R**.

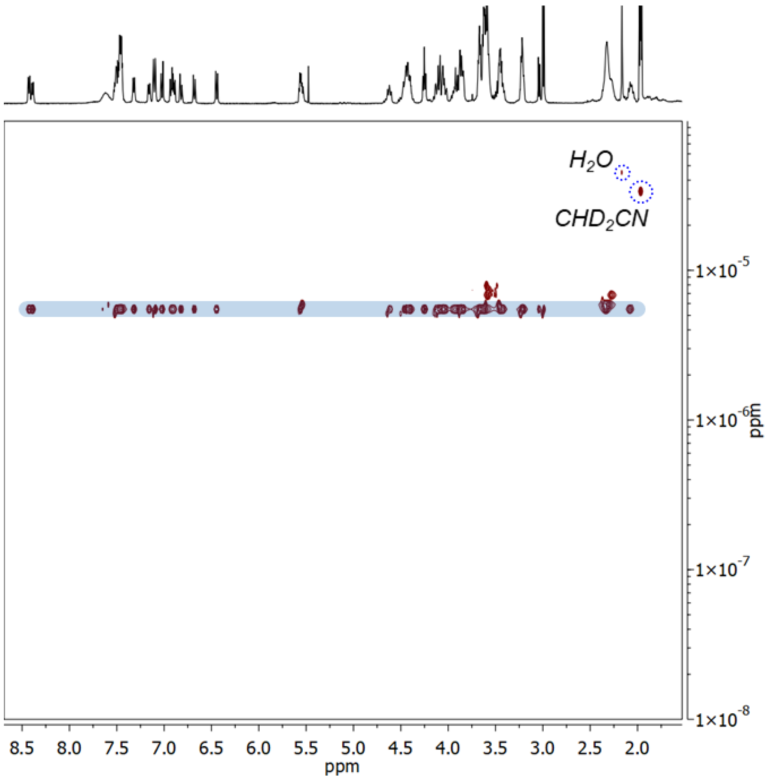

Figure S64 ^{1}H DOSY NMR spectrum (100 MHz, CD_3CN, 25 ºC) of hetero[4]rotaxane ***t*-H[4]R**

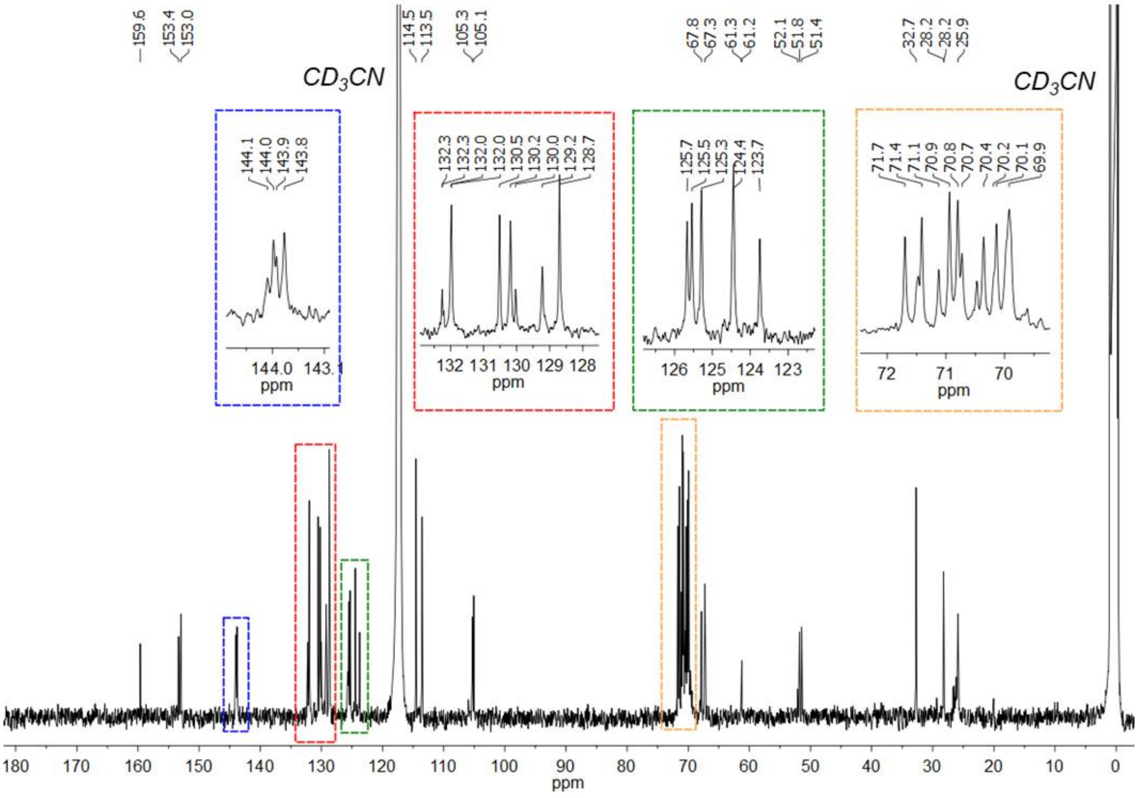

Figure S65. ^{13}C {^{1}H} NMR spectrum (100 MHz, CD_3CN) of hetero[4]rotaxane ***t*-H[4]R**.

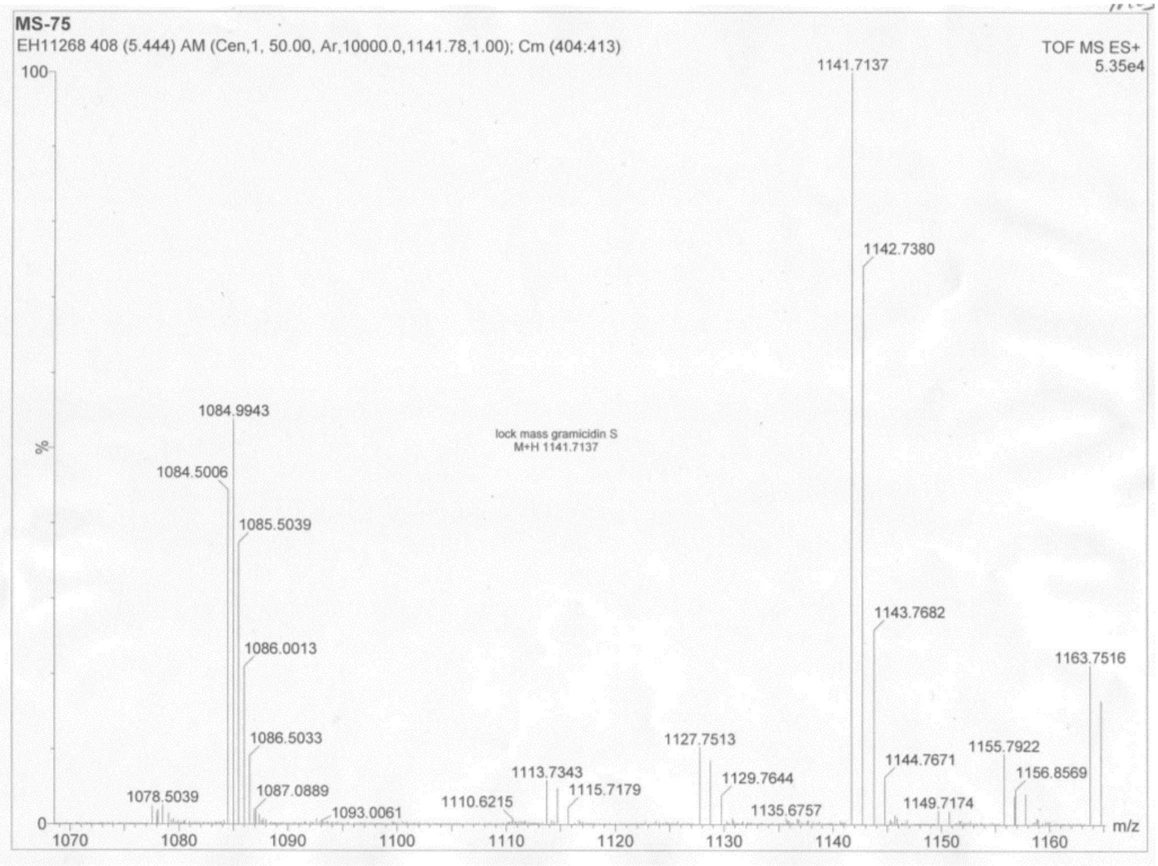

Figure S66. Partial HRMS showing the ion [**1** + **DN32C8** + (2 × **21C6**) + (2 × PF_6) + 2H]$^{2+}$ for rotaxane ***t*-H[4]R**.

References

1 I. R. Fernando, Y. Mo and G. Mezei, *CrystEngComm*, 2014, **16**, 7320–7333.

2 S. Dasgupta and J. Wu, *Chem. Sci.*, 2012, **3**, 425–432.

3 V. N. Vukotic, C. A. O'Keefe, K. Zhu, K. J. Harris, C. To, R. W. Schurko and S. J. Loeb, *J. Am. Chem. Soc.*, 2015, **137**, 9643–9651.

4 H. Shanan-Atidi and K. H. Bar-Eli, *J. Phys. Chem.*, 1970, **74**, 961–963.

5 Gaussian 16, Revision B.01, M. J. Frisch, G. W. Trucks, H. B. Schlegel, G. E. Scuseria, M. A. Robb, J. R. Cheeseman, G. Scalmani, V. Barone, G. A. Petersson, H. Nakatsuji, X. Li, M. Caricato, A. V. Marenich, J. Bloino, B. G. Janesko, R. Gomperts, B. Mennucci, H. P. Hratchian, J. V. Ortiz, A. F. Izmaylov, J. L. Sonnenberg, D. Williams-Young, F. Ding, F. Lipparini, F. Egidi, J. Goings, B. Peng, A. Petrone, T. Henderson, D. Ranasinghe, V. G. Zakrzewski, J. Gao, N. Rega, G. Zheng, W. Liang, M. Hada, M. Ehara, K. Toyota, R. Fukuda, J. Hasegawa, M. Ishida, T. Nakajima, Y. Honda, O. Kitao, H. Nakai, T. Vreven, K. Throssell, J. A. Montgomery, Jr., J. E. Peralta, F. Ogliaro, M. J. Bearpark, J. J. Heyd, E. N. Brothers, K. N. Kudin, V. N. Staroverov, T. A. Keith, R. Kobayashi, J. Normand, K. Raghavachari, A. P. Rendell, J. C. Burant, S. S. Iyengar, J. Tomasi, M. Cossi, J. M. Millam, M. Klene, C. Adamo, R. Cammi, J. W. Ochterski, R. L. Martin, K. Morokuma, O. Farkas, J. B. Foresman, and D. J. Fox, Gaussian, Inc., Wallingford CT, 2016.

6 J.-D. Chai and M. Head-Gordon, *Phys. Chem. Chem. Phys.*, 2008, **10**, 6615–6620.

7 M. M. Francl, W. J. Pietro, W. J. Hehre, J. S. Binkley, M. S. Gordon, D. J. DeFrees and J. A. Pople, *J. Chem. Phys.*, 1982, **77**, 3654–3665.

8 G. Scalmani and M. J. Frisch, *J. Chem. Phys.*, 2010, **132**, 114110.

9 L. Chen, Y. M. Zhang and Y. Liu, *J. Phys. Chem. B*, 2012, **116**, 9500–9506.

10 T. Liu, X. Miao, X. Geng, A. Xing, L. Zhang, Y. Meng and X. Li, *New J. Chem.*, 2016, **40**, 3432–3439.
